# Bicyclic Basic Merbarone Analogues as Antiproliferative Agents

**DOI:** 10.3390/molecules26030557

**Published:** 2021-01-21

**Authors:** Andrea Spallarossa, Matteo Lusardi, Chiara Caneva, Aldo Profumo, Camillo Rosano, Marco Ponassi

**Affiliations:** 1 Department of Pharmacy, University of Genova, Viale Benedetto XV, 3, 16132 Genova, Italy; matteo.lusardi@edu.unige.it (M.L.); chiara.caneva.cc@gmail.com (C.C.); 2 IRCCS Ospedale Policlinico San Martino, Proteomics and Mass Spectrometry Unit, L.go R. Benzi 10, 16132 Genova, Italy; aldo.profumo@hsanmartino.it (A.P.); camillo.rosano@hsanmartino.it (C.R.); marco.ponassi@hsanmartino.it (M.P.)

**Keywords:** pyrimido-pyrimidine derivatives, antiproliferative agents, structure activity relationships (SAR) study, docking simulation

## Abstract

Pyrimido-pyrimidine derivatives have been developed as rigid merbarone analogues. In a previous study, these compounds showed potent antiproliferative activity and efficiently inhibited topoisomerase IIα. To further extend the structure–activity relationships on pyrimido-pyrimidines, a novel series of analogues was synthesized by a two-step procedure. Analogues **3**–**6** bear small alky groups at positions 1 and 3 of the pyrimido-pyrimidine scaffold whereas at position 6a (4-chloro)phenyl substituent was inserted. The basic side chains introduced at position 7 were selected on the basis of the previously developed structure–activity relationships. The antiproliferative activity of the novel compounds proved to be affected by both the nature of the basic side chain and the substituents on the pyrimido-pyrimidine moiety. Derivatives **5d** and **5e** were identified as the most promising molecules still showing reduced antiproliferative activity in comparison with the previously prepared pyrimido-pyrimidine analogues. In topoisomerase IIα-**5d** docking complex, the ligand would poorly interact with the enzyme and assume a different orientation in comparison with **1d** bioactive conformation.

## 1. Introduction

Topoisomerases (Topo) are essential enzymes for DNA replication that catalyze the alteration of DNA topology through transient DNA strand breakage [1,2]. On the basis of their ability to cut one or both strands of a DNA double helix, topoisomerases can be classified into type I (EC 5.99.1.2) and type II (EC 5.99.1.3) enzymes [3,4]. Type II topoisomerase (TopoII) enzymes represent validated molecular targets for clinically used antitumor drugs such as amsacrine, etoposide, doxorubicin and mitoxantrone [5]. However, drug resistance and possible side effects of TopoII-targeting drugs call for novel and safer TopoII agents [5,6,7]. TopoII targeting agents can be classified as poisons or catalytic inhibitors [8,9]. In particular, TopoII poisons (e.g., etoposide, amsacrine, and mitoxantrone) are able to stabilize the cleavage complex inducing DNA double strand breaks whereas the catalytic inhibitors (e.g., aclarubicin, novobiocin, suramin, ICRF-193) block a specific step of TopoII catalytic cycle (e.g., ATP binding) [10,11].

**Figure 1:**
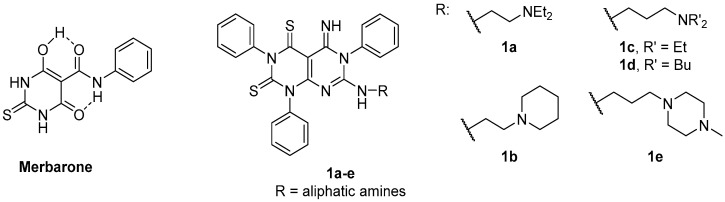
Chemical structures of merbarone and its rigid analogues **1**.

Merbarone (Figure 1) is a thio-barbituric catalytic TopoII inhibitor that blocks the proliferation of several cancer cell lines [12]. In clinical trials, merbarone showed nephrotoxicity issues and poor efficacy potentially ascribable to its high ionizability (and therefore poor bioavailability) at physiological pH [10,13,14]. With the aim of preparing merbarone analogues endowed with improved pharmacodynamic and pharmacokinetic properties, we previously synthesized compounds **1** (Figure 1) as conformationally constrained analogues in which the H-bonded pseudo-bicyclic structure of merbarone has been converted into a pyrimido-pyrimidine moiety [15,16]. In cell-based assays, derivatives **1** showed interesting antiproliferative properties and the nature of the substituent at position 7 of the pyrimido-pyrimidine scaffold was found to deeply affect the biological activity. In particular, compounds **1a**–**e** (Figure 1) were identified as the most promising compounds with (sub)micromolar IC_50_ values against various cell lines (namely, MT-4, HeLa and MCF-7). Furthermore, selected members of this series were able to inhibit TopoIIα enzyme [16].

**Figure 2:**
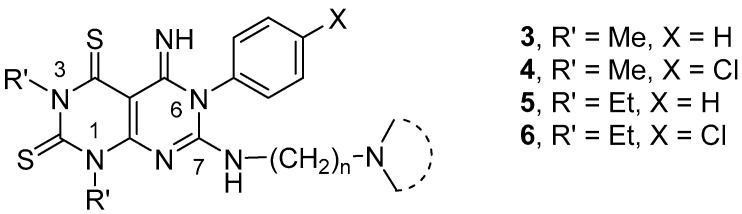
Chemical structures of pyrimido-pyrimidine derivatives **3**–**6**.

Owing to the interest in developing novel merbarone analogues [17,18] and in order to extend the structure activity relationships (SARs) of compounds **1**, derivatives **3**–**6** (Figure 2) were designed and synthesized. The novel derivatives were substituted at position 7 with the most promising side chain identified in the previous studies [15,16], and bear at positions 1 and 3 small alkyl chains (i.e., Me or Et groups). Moreover, the effect on activity of the 4-chloro substitution on the phenyl ring at position 6 was evaluated.

## 2. Results and Discussion

**Scheme 1:**
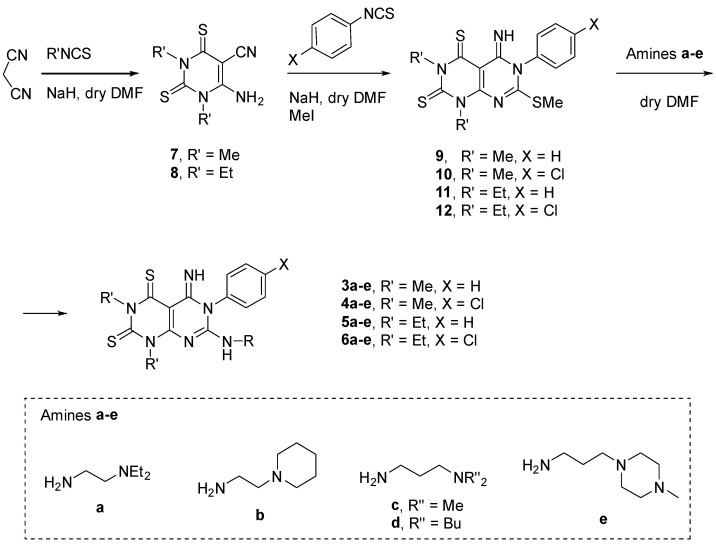
Chemical synthesis of derivatives **3**–**6**.

The synthesis of compounds **3**–**6** was carried out on the basis of previously developed procedures [15,16]. Briefly, the condensation of malononitrile with two equivalents of methyl or ethyl isothiocyanate in basic conditions led to the formation of the pyrimido derivatives **7** and **8** (Scheme 1). Differently from what was observed using phenyl isothiocyanate [15,16], neither the use of different bases (i.e., NaH, t-BuOK) or the use of three equivalents of alkyl heterocumulenes led to the isolation of the pyrimido-pyrimidine analogue. Derivatives **7** and **8** were then reacted, in a one-pot condensation, with phenyl or 4-chlorophenyl isothiocyanate and iodomethane to afford **9**–**12** (Scheme 1). Finally, the S-methylated compounds were converted into the final derivatives **3**–**6** by reacting with the proper amine (1.1. equivalents, Scheme 1). The different reactivity of **9**–**12** intermediates towards amines a–e required different reaction conditions and the desired compounds were obtained in moderate to good yields (Table 1).

The antiproliferative properties of compounds **3**–**6** were assessed in SKOV-3 and MCF-7 cell lines by MTT assay (Table 2). The derivatives were assayed at a fixed concentration (10 µM) and compound **1d** (Figure 1) was considered as reference compound. Overall, the tested derivatives proved to be able to inhibit the proliferation of SKOV-3 and/or MCF-7 cells less efficiently than the reference compound, highlighting that the replacement of the N-phenyl rings at positions 1 and 3 with small alkyl chains was detrimental for the antiproliferative activity. Compounds **3c**,**d**, **5d**,**e** and **6e** showed significant cytotoxicity (i.e., growth inhibition percentage greater than 30%) against SKOV-3 cells, derivative **5d** being the most active molecule (Table 2). Within the 1,3-dimethyl substituted compounds, the 4-chloro substitution of the phenyl ring led to the loss of antiproliferative activity (compare **3c** with **4c** and **3d** with **4d**, Table 2). Conversely, within the 1,3-diethyl substituted series (derivatives **5** and **6**), the effects of the phenyl substitution on activity are related to the nature of the basic chain. Thus, the 3-(4-methylpiperazin-1-yl)propyl derivatives **5e** (N-phenyl) and **6e** (N-4-chlorophenyl) showed similar antiproliferative properties whereas the 4-chloro substitution on the phenyl ring of the 3-(dibutyl-amino)propyl compound 5d led to the loss of activity (compare **5d** with **6d**, Table 2). Compounds **3c**,**d**, **5d** and **6e** also kept their antiproliferative activity (even though reduced) against MCF-7 cells whose proliferation proved to be more affected than that of SKOV-3 cells by derivatives **5e** and **6d** (Table 2).

**Figure 3:**
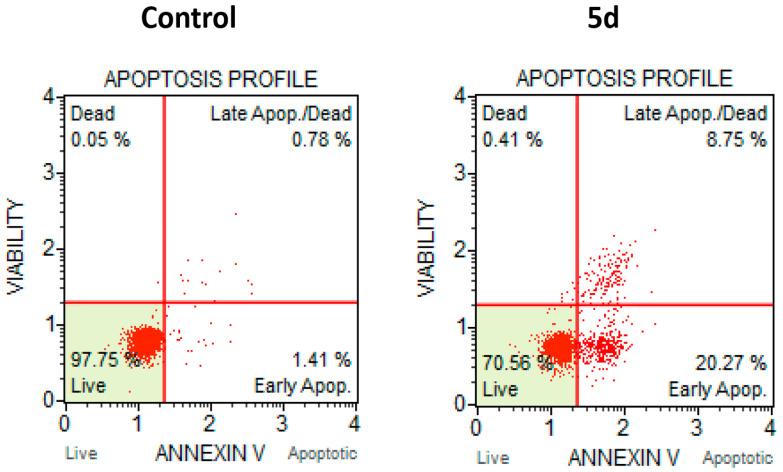
Apoptosis profile performed with Muse™ Annexin V & Dead Cell Assay. SKOV-3 cells were treated for 48 h with compound **5d** at 2.5 µM concentration or with the same amount of DMSO alone (Control).

Moreover, to further investigate the ability of compound **5d** to induce apoptotic cell death in cultured cell lines, as already demonstrated for other TopoIIα inhibitors [19], we performed the Muse™ Annexin V & Dead Cell Assay using the SKOV-3 ovarian adenocarcinoma cell line. As shown in Figure 3, the 48 h treatment with **5d** caused an approximately 30% increase of apoptotic cells when compared to the control (DMSO treated). The obtained profile showed the bigger increase in early apoptotic cells (20%) while the late apoptotic cells showed an increase of about 8%.

**Figure 4:**
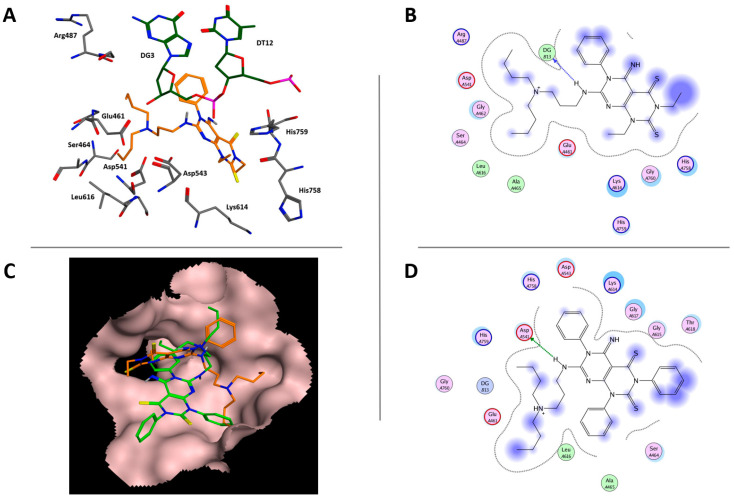
(**A**) Docking pose of **5d** within TopoIIα binding site. (**B**) Ligplot of TopoIIα-DNA-**5b** complex. (**C**) different binding orientations of **1d** (green carbon atoms) and **5d** (orange carbon atoms) in TopoIIα binding site. (**D**) Ligplot of TopoIIα-DNA-**1b** complex.

The TopoIIα inhibitors 1 [16] are chemically related to the apoptosis inducer **5d**. On this basis, to assess whether TopoIIα could represent a biological target for **5d**, a docking simulation (Autodock 4.2) [20] was carried out considering **1d** as template molecule. As the ionization state of compounds could affect the interaction with the biological target [21], the pKa values of compounds **1d** and **5d** were calculated (ACD labs software). Both ligands showed a pKa value of 9.60, thus highlighting that, at physiological pH, the tertiary amine group of the two molecules would be in a protonated form. According to the docking simulation, the ternary complex TopoIIα-DNA-**5d** would be stabilized by a hydrogen bond involving the ligand NH group at position 7 and the phosphate function in the DNA strain. Additional contacts would occur between: (i) the thiocarbonyl group at position 2 and the side chain of His578 and (ii) the 3-(dibutyl-amino)propyl substituent and the side chain of Asp541 and Arg487 (Figure 4A,B). The calculated Ki value of the docking pose was 9.55 mM, thus foreseeing a weak interaction between the ligand and the TopoIIa enzyme. Furthermore, **5d** would assume a different binding orientation in comparison with that calculated for the tri-phenyl substituted derivative **1d** (Figure 4C) which showed a calculated Ki value of 772 µM. The TopoIIα-DNA-**1d** complex would be stabilized mainly by a hydrogen bond between the NH group at position 7 and the side chain of Asp541. Furthermore, a polar interaction between the protonated tertiary amine group of **1d** and the DNA phospho-diesteric group would occur. Additional Van der Waals interactions would involve the phenyl ring at position 6 and Asp543 and Lys614 side chains whereas the dibutyl moiety would be in contact with DG3, Glu461, His758 and Leu616 (Figure 4D).

## 3. Materials and Methods

### 3.1. Chemistry

All building blocks used are commercially available. Amines (**a**–**e**, Scheme 1), isothiocyanates, malononitrile and 60% sodium hydride dispersion in mineral oil were purchased from Chiminord and Aldrich Chemical (Milan, Italy). Solvents were reagent grade. DMF was dried on molecular sieves (5 Å 1/16” inch pellets). Unless otherwise stated, all commercial reagents were used without further purification. Organic solutions were dried over anhydrous sodium sulphate. A thin layer chromatography (TLC) system for routine monitoring the course of reactions and confirming the purity of analytical samples employed aluminum-backed silica gel plates (Merck DC-Alufolien Kieselgel 60 F_254_): CHCl_3_ was used as developing solvent, and detection of spots was made by UV light and/or by iodine vapors. Yields were not optimized. Melting points were determined on a Fisher-Johns apparatus and are uncorrected. IR spectra were recorded on a Perkin Elmer 398 spectrometer as KBr discs. NMR spectra were recorded on a Varian Gemini 200 or Bruker Avance DPX 300 Spectrometer or JEOL JNM-ECZR instrument. Chemical shifts were reported in δ (ppm) units relative to the internal standard tetramethyl-silane, and the splitting patterns were described as follows: s (singlet), t (triplet) and m (multiplet). The first order values reported for coupling constants J were given in Hz. Elemental analyses were performed by an EA1110 Elemental Analyser (Fison-Instruments, Milan, Italy); all compounds were analyzed for C, H, N and S and the analytical results were within ± 0.4% of the theoretical values. For mass spectra collection, each sample was dissolved in DMSO (final concentration: 10 mM) and after further dilution in acetonitrile (final concentration 100 nM), it was analyzed by flow injection mass spectrometry (FIA-MS). Briefly, five microliters of sample were injected into an eluent flow containing 0.1% formic acid in acetonitrile, generated by a Vanquish UHPLC system (Thermo Fisher Scientific, San Jose, CA, USA). The flow rate was 100 µL/min. The eluent was directly sent to a Q-Exactive Plus Orbitrap mass spectrometer (Thermo Fisher Scientific, San Jose, CA, USA) equipped with a heated electrospray ion source (HESI-II). Prior to each series of acquisitions, the mass spectrometer was externally calibrated with Positive Ion Calibration Solution (Thermo Fisher Scientific, San Jose, CA, USA). The following operating parameters were applied: resolution 35,000; sheath and auxiliary gas flow rate were 35 and 10 respectively; spray voltage 3.5 kV; capillary temperature 250 °C; S-lens RF level 100. The autogain control (AGC) was optimized at 1e6 with a maximum injection time (maxIT) of 250 ms. Full scan data were processed with Xcalibur version 4.1 (Thermo Fisher Scientific, San Jose, CA, USA). High resolution mass spectra, ranging from 100 to 600 *m*/*z*, were acquired in positive ion mode and the identity of each analyte was confirmed by comparing the experimental data with both their theoretical molecular weight (+H^+^) and their expected isotopic pattern. IR, NMR and mass spectra are reported in Supplementary Materials, Figures S1–S88.

#### 3.1.1. General Procedure for the Synthesis of Compounds **7** and **8**

To an ice-cooled, dry DMF solution (30 mL) of malononitrile (760 mg, 10 mmol) and the appropriate isothiocyanate (20 mmol), 60% sodium hydride dispersion in mineral oil (870 mg, 20 mmol) was added. The reaction mixture was stirred at room temperature for 15h. After addition of cool water (50 mL) and 2N HCl (pH = 0), a solid precipitated. The crude solid was collected by filtration and purified by crystallization from EtOH/DCM mixture.

6-amino-1,3-dimethyl-2,4-dithioxo-1,2,3,4-tetrahydropyrimidine-5-carbonitrile (**7**). Yield: 26%; m.p.: 268–271 °C (EtOH/DCM). IR (KBr) cm^−1^ 3407, 3315, 3192, 2217. ^1^H NMR (d_6_-DMSO) δ 3.81 (s, 3H, CH_3_); 4.11 (s, 3H, CH_3_); 8.38 (s, 2H, NH_2_). ^13^C NMR (d_6_-DMSO) δ 44.2, 85.2, 116.9, 152.9, 175.7, 181.6. MS *m*/*z* 213.03 [M + H^+^]. Calcd. for C_7_H_8_N_4_S_2_, %: C: 37.87; H: 3.88; N: 24.54; S: 33.70. Found, %: C: 37.94; H: 3.64; N: 24.52; S: 33.52.

6-amino-1,3-diethyl-2,4-dithioxo-1,2,3,4-tetrahydropyrimidine-5-carbonitrile (**8**). Yield: 95%; m.p.: 230–231 °C (EtOH/DCM). IR (KBr) cm^-1^ 3493, 3331, 2218, 1682. ^1^H NMR (d_6_-DMSO) δ 1.10–1.38 (m, 6H, 2 × CH_3_); 3.25–3.50 (m, 4H, 2 × CH_2_); 8.36 (s, 2H, NH_2_). ^13^C NMR (d_6_-DMSO) δ 11.3, 11.5, 46.6, 50.7, 85.3, 117.1, 151.7, 174.2, 180.6. MS *m*/*z* 241.06 [M + H^+^]. Calcd. for C_9_H_12_N_4_S_2_, %: C: 44.97; H: 5.03; N: 23.31; S: 26.68. Found, %: C: 44.70; H: 4.93; N: 23.12; S: 26.43.

#### 3.1.2. General Procedure for the Synthesis of Compounds **9**–**12**

To an ice-cooled, dry DMF solution of **7** or **8** (10 mmol), the appropriate isothiocyanate (11 mmol) and 60% sodium hydride dispersion in mineral oil (870 mg, 20 mmol) were added. The reaction mixture was stirred at room temperature for 12 h and then methyl iodide (0.685 mL, 11 mmol) was added. The resulting mixture was stirred for 12 h and cool water (50 mL) was added. A solid precipitated and the crude material was collected by filtration, dissolved in DCM (15 mL), dried with anhydrous Na_2_SO_4_ and purified by crystallization from the suitable solvent or solvent mixture.

5-imino-1,3-dimethyl-7-(methylthio)-6-phenyl-5,6-dihydropyrimido[4,5-d]pyrimidine-2,4(1H,3H)-dithione (**9**). Yield: 78%; m.p.: 249–251 °C (DCM/Et_2_O). IR (KBr) cm^−1^ 3177, 1614. ^1^H NMR (CDCl_3_) δ 2.53 (s, 3H, SCH_3_); 4.16 (s, 3H, NCH_3_); 4.36 (s, 3H, NCH_3_); 7.23–7.39 and 7.48–7.68 (m, 5H, arom. H); 11.75 (bs, 1H, NH). ^13^C NMR (CDCl_3_) δ 16.2, 38.8, 44.1, 102.1, 128.8, 130.9, 131.3, 135.4, 148.7, 155.8, 169.8, 175.3, 182.3. MS *m*/*z* 362.06 [M + H^+^]. Calcd. for C_15_H_15_N_5_S_3_, %: C: 49.84; H: 4.18; N: 19.37; S: 26.61. Found, %: C: 49.86; H: 4.42; N: 19.20; S: 26.48.

6-(4-chlorophenyl)-5-imino-1,3-dimethyl-7-(methylthio)-5,6-dihydro-pyrimido(4,5-d)pyrimidine-2,4(1H,3H)-dithione (**10**). Yield: 48%; m.p.: 230–232 °C (DCM/Et_2_O). IR (KBr) cm^−1^ 3167, 1673, 1615. ^1^H NMR (CDCl_3_) δ 2.59 (s, 3H, SCH_3_); 4.18 (s, 3H, NCH_3_); 4.35 (s, 3H, NCH_3_); 7.21–7.65 (m, 5H, arom. H); 12.38 (bs, 1H, NH). ^13^C NMR (CDCl_3_) δ 16.3, 38.9, 44.2, 101.5, 130.3, 131.5, 133.4, 137.9, 148.8, 155.7, 169.6, 175.2, 182.4. MS *m*/*z* 396.02 [M + H^+^]. Calcd. for C_15_H_14_ClN_5_S_3_, %: C: 45.50; H: 3.56; N: 17.69; S: 24.29. Found, %: C: 45.32; H: 3.53; N: 17.99; S: 24.31.

1,3-diethyl-5-imino-7-(methylthio)-6-phenyl-5,6-dihydropyrimido[4,5-d]pyrimidine-2,4(1H,3H)-dithione (**11**). Yield: 64%; m.p.: 189–190 °C (Et_2_O). IR (KBr) cm^−1^ 3433, 3207, 1621. ^1^H NMR (d_6_-DMSO) δ 1.31–1.38 (m, 6H, 2×CH_3_); 2.49 (s, 3H, SCH_3_); 4.84–4.86 (m, 2H, CH_2_); 4.88–5.17 (m, 2H, CH_2_); 7.33–7.34 e 7.49–7.58 (m, 5H, arom. H); 11.46 (s, 1H, NH). ^13^C NMR (d_6_-DMSO) δ 10.3, 11.4, 46.0, 49.4, 50.0, 103.4, 128.9, 129.6, 137.2, 148.0, 151.1, 154.5, 169.2, 173.4, 180.6. MS *m*/*z* 390.09 [M + H^+^]. Calcd. for C_17_H_19_N_5_S_3_, %: C: 52.41; H: 4.92; N: 17.98; S: 24.69. Found, %: C: 52.20; H: 4.64; N: 17.91; S: 25.05.

6-(4-chlorophenyl)-1,3-diethyl-5-imino-7-(methylthio)-5,6-dihydropyrimido[4,5-d]pyrimidine-2,4(1H,3H)-dithione (**12**). Yield: 90%; m.p.: 247–249 °C (Et_2_O). IR (KBr) cm^−1^ 3151, 1619. ^1^H NMR (CDCl_3_) δ 1.27–1.53 (m, 6H, 2 × CH_3_); 2.53 (s, 3H, NCH_3_); 4.86–5.07 (m, 2H, CH_2_); 5.18–5.51 (m, 2H, CH_2_); 7.26–7.40 and 7.52–7.64 (m, 4H, arom. H); 11.88 (bs, 1H, NH). ^13^C NMR (CDCl_3_) δ 10.0, 12.0, 16.5, 47.5, 51.3, 98.7, 130.4, 131.2, 132.7, 140.0, 149.0, 156.6, 170.4, 173.3, 181.3. MS *m*/*z* 424.05 [M + H^+^]. Calcd. for C_17_H_18_ClN_5_S_3_, %: C: 48.16; H: 4.28; N: 16.52; S: 22.69. Found, %: C: 48.47; H: 4.10; N: 16.44; S: 22.45.

#### 3.1.3. General Procedure for the Synthesis of Compounds **3–6**

To a dry DMF (10 mL) solution of the suitable derivative 9–12 (1 mmol), the appropriate amine (1.2 mmol) was added. The reaction mixture was stirred at variable time and temperature as detailed in Table 1. After the addition of water (10 mL), a solid separated. The crude precipitate was collected by filtration and purified by crystallization from DCM/MeOH mixture.

7-((2-(diethylamino)ethyl)amino)-5-imino-1,3-dimethyl-6-phenyl-5,6-dihydro-pyrimido[4,5-d]pyrimidine-2,4(1H,3H)-dithione (**3a**). IR (KBr) cm^−1^ 3175, 2968, 1626. ^1^H NMR (CDCl_3_) δ 0.77–0.94 (m, 6H, 2×CH_3_); 2.38–2.71 (m, 6H, 3×CH_2_N); 3.41–3.59 (m, 2H, CH_2_N); 4.01–4.10 (m, 3H, CH_3_); 4.29–4.40 (m, 3H, CH_3_); 7.21–7.36 (m, 3H, 2 arom. H and NH); 7.52–7.70 (m, 3H, arom. H); 11.99 (bs, H, NH). ^13^C NMR (CDCl_3_) δ 11.0, 38.4, 39.9, 43.6, 46.8, 50.3, 128.8, 130.1, 131.1, 135.1, 151.4, 151.7, 156.7, 175.9, 181.1. MS *m*/*z* 430.18 [M + H^+^]. Calcd. for C_20_H_27_N_7_S_2_, %: C: 55.92; H: 6.33; N: 22.82; S: 14.93. Found, %: C: 55.69; H: 6.96; N: 22.57; S: 14.77.

5-imino-1,3-dimethyl-6-phenyl-7-((2-(piperidin-1-yl)ethyl)amino)-5,6-dihydropyrimido[4,5-d]pyrimidine-2,4(1H,3H)-dithione (**3b**). IR (KBr) cm^−1^ 3174, 2928, 1626. ^1^H NMR (CDCl_3_) δ 1.28–1.47 (m, 6H, 3×CH_2_); 2.30–2.51 (m, 6H, 3×CH_2_N); 3.42–3.59 (m, 2H, CH_2_N); 4.01–4.11 (m, 3H, CH_3_); 4.30–4.41 (m, 3H, CH_3_); 7.23–7.38 (m, 3H, 2 arom. H and NH); 7.54–7.74 (m, 3H, arom. H); 11.91 (bs, H, NH). ^13^C NMR (CDCl_3_) δ 22.5, 23.2, 38.5, 39.9, 43.8, 53.7, 55.6, 97.1, 128.8, 132.0, 151.8, 158.3, 175.9, 181.9. Calcd. for C_21_H_27_N_7_S_2_, %: C: 57.11; H: 6.16; N: 22.20; S: 14.52. Found, %: C: 57.25; H: 6.00; N: 22.03; S: 14.27.

7-((3-(dimethylamino)propyl)amino)-5-imino-1,3-dimethyl-6-phenyl-5,6-dihydropyrimido[4,5-d]pyrimidine-2,4(1H,3H)-dithione (**3c**). IR (KBr) cm^−1^ 3173, 3052, 1621. ^1^H NMR (CDCl_3_) δ 1.62–1.89 (m, 8H, 2×CH_3_ and CH_2_); 2.29–2.51 (m, 2H, CH_2_N); 3.49–3.65 (m, 2H, CH_2_N); 3.95–4.05 (m, 3H, CH_3_N); 4.23–4.40 (m, 3H, CH_3_N); 7.20–7.35 (m, 3H, 2 arom. H and NH); 7.52–7.70 (m, 3H, arom. H); 11.64 (bs, H, NH). ^13^C NMR (CDCl_3_) δ 23.5, 38.6, 43.8, 44.0, 99.1, 128.9, 131.2, 131.9, 152.1, 152.5, 157.0, 175.6, 181.4. Calcd. for C_19_H_25_N_7_S_2_, %: C: 54.91; H: 6.06; N: 23.59; S: 15.43. Found, %: C: 55.25; H: 5.88; N: 23.38; S: 15.27.

7-((3-(dibutylamino)propyl)amino)-5-imino-1,3-dimethyl-6-phenyl-5,6-dihydropyrimido[4,5-d]pyrimidine-2,4(1H,3H)-dithione (**3d**). IR (KBr) cm^−1^ 3202, 3055, 1622. ^1^H NMR (CDCl_3_) δ 0.81–1.04 (m, 6H, 2×CH_3_);1.15–1.40 (m, 8H, 4×CH_2_);1.65–1.82 (m, 2H, CH_2_); 2.14–2.36 (m, 4H, 2×CH_2_N); 2.48–2.63 (m, 2H, CH_2_N); 3.53–3.67 (m, 2H, CH_2_N); 3.97–4.15 (m, 3H, CH_3_N); 4.30–4.42 (m, 3H, CH_3_N); 7.23–7.42 (m, 3H, 2 arom. H and NH); 7.51–7.70 (m, 3H, arom. H); 11.76 (bs, H, NH). ^13^C NMR (CDCl_3_) δ 13.8, 20.1, 23.5, 38.4, 43.6, 44.7, 59.2, 101.1, 129.1, 129.9, 131.0, 135.9, 151.8, 153.0, 156.4, 175.7, 181.1. Calcd. for C_25_H_37_N_7_S_2_, %: C: 60.08; H: 7.64; N: 19.62; S: 12.83. Found, %: C: 60.21; H: 7.76; N: 19.53; S: 12.51.

5-imino-1,3-dimethyl-7-((3-(4-methylpiperazin-1-yl)propyl)amino)-6-phenyl-5,6-dihydropyrimido[4,5-d]pyrimidine-2,4(1H,3H)-dithione (**3e**). IR (KBr) cm^−1^ 3186, 3032, 1627. ^1^H NMR (CDCl_3_) δ 1.70–1.87 (m, 2H, CH_2_); 2.28–2.60 (m, 13H, CH_3_, 5×CH_2_); 3.49–3.61 (m, 2H, CH_2_N); 4.00–4.14 (s, 3H, CH_3_); 4.31–4.41 (s, 3H, CH_3_); 7.25–7.39 (m, 3H, 2 arom. H and NH); 7.56–7.72 (m, 3H, arom. H); 11.76 (bs, H, NH). ^13^C NMR (CDCl_3_) δ 25.1, 38.5, 40.9, 43.7, 45.4, 52.2, 53.8, 55.2, 129.1, 130.5, 131.3, 151.7, 152.9, 156.1, 175.6, 181.3. Calcd. for C_22_H_30_N_8_S_2_, %: C: 56.14; H: 6.42; N: 23.81; S: 13.63. Found, %: C: 56.02; H: 6.66; N: 23.75; S: 13.37.

6-(4-chlorophenyl)-7-((2-(diethylamino)ethyl)amino)-5-imino-1,3-dimethyl-5,6-dihydropyrimido(4,5-d)pyrimidine-2,4(1H,3H)-dithione (**4a**). IR (KBr) cm^−1^ 3181, 2966, 1626. ^1^H NMR (CDCl_3_) δ 0.78–0.89 (m, 6H, 2×CH_3_); 2.31–2.46 (m, 4H, 2×CH_2_N); 2.53–2.60 (m, 2H, CH_2_N); 3.42–3.52 (m, 2H, CH_2_N); 4.01–4.10 (m, 3H, CH_3_); 4.35–4.40 (m, 3H, CH_3_); 7.21–7.30 (m, 3H, 2 arom. H and NH); 7.52–7.61 (m, 2H, arom. H); 11.71 (bs, H, NH). ^13^C NMR (CDCl_3_) δ 11.0, 38.4, 39.7, 43.6, 46.8, 50.5, 99.8, 130.4, 131.3, 133.7, 136.2, 151.4, 151.9, 156.5, 175.8, 181.1. Calcd. for C_20_H_26_ClN_7_S_2_, %: C: 51.76, H: 5.65, N: 21.13, S: 13.82. Found, %: C: 51.75, H: 5.62, N: 20.97, S: 13.47.

6-(4-chlorophenyl)-5-imino-1,3-dimethyl-7-((2-(piperidin-1-yl)ethyl)amino)-5,6-dihydropyrimido(4,5-d)pyrimidine-2,4(1H,3H)-dithione (**4b**). IR (KBr) cm^−1^ 3169, 2935, 1626-. ^1^H NMR (CDCl_3_) δ 1.39–1.51 (m, 6H, 3×CH_2_); 2.30–2.60 (m, 6H, 3×CH_2_N); 3.44–3.60 (m, 2H, CH_2_N); 4.00–4.10 (s, 3H, CH_3_); 4.35–4.40 (s, 3H, CH_3_); 7.25–7.34 (m, 3H, 2 arom. H and NH); 7.58–7.66 (m, 3H, H arom.); 11.80 (bs, H, NH). ^13^C NMR (CDCl_3_) δ 23.6, 24.7, 35.1, 37.2, 38.4, 43.7, 53.7, 55.7, 99.6, 130.4, 131.4, 151.5, 175.8, 181.2. Calcd. for C_21_H_26_ClN_7_S_2_, %: C: 52.98, H: 5.50, N: 20.60, S: 13.47. Found, %: C: 53.19, H: 5.73, N: 20.60, S: 13.27.

6-(4-chlorophenyl)-7-((3-(dimethylamino)propyl)amino)-5-imino-1,3-dimethyl-5,6-dihydropyrimido[4,5-d]pyrimidine-2,4(1H,3H)-dithione (**4c**). IR (KBr) cm^−1^ 3179, 3048, 1627. ^1^H NMR (CDCl_3_) δ 1.58–1.95 (m, 8H, 2×CH_3_ and CH_2_); 2.31–2.41 (m, 2H, CH_2_N); 3.53–3.63 (m, 2H, CH_2_N); 3.98–4.12 (m, 3H, CH_3_N); 4.13–4.40 (m, 3H, CH_3_N); 7.12–7.32 (m, 3H, 2 arom. H and NH); 7.51–7.67 (m, 2H, H arom.); 11.63 (bs, H, NH). ^13^C NMR (CDCl_3_) δ 26.7, 36.1, 37.1, 43.2, 47.0, 58.7, 98.6, 125.6, 128.7, 138.9, 153.2, 159.0, 172.3, 175.5, 182.7. Calcd. for C_19_H_24_ClN_7_S_2_, %: C: 50.71, H: 5.38, N: 21.79, S: 14.25. Found, %: C: 50.51, H: 5.13, N: 21.70, S: 14.27.

6-(4-chlorophenyl)-7-((3-(dibutylamino)propyl)amino)-5-imino-1,3-dimethyl-5,6-dihydropyrimido[4,5-d]pyrimidine-2,4(1H,3H)-dithione (**4d**). IR (KBr) cm^−1^ 3174, 3053, 1621. ^1^H NMR (CDCl_3_) δ 0.81–1.0 (m, 6H, 2×CH_3_); 1.13–1.35 (m, 8H, 4×CH_2_); 1.61–1.79 (m, 2H, CH_2_); 2.08–2.31 (m, 4H, 2×CH_2_N); 2.47–2.65 (m, 2H, CH_2_N); 3.51–3.66 (m, 2H, CH_2_N); 3.98–4.14 (m, 3H, CH_3_N); 4.20–4.39 (m, 3H, CH_3_N); 7.15–7.29 (m, 3H, 2 arom. H and NH); 7.48–7.61 (m, 2H, H arom.); 11.67 (bs, H, NH). ^13^C NMR (CDCl_3_) δ 14.1, 20.7, 24.4, 27.0, 38.4, 43.6, 51.3, 54.0, 101.1, 130.7, 131.2, 134.2, 136.0, 151.6, 152.6, 156.3, 175.6, 181.1. Calcd. for C_25_H_36_ClN_7_S_2_, %: C: 56.21, H: 6.79, N: 18.35, S: 12.01. Found, %: C: 55.93, H: 6.62, N: 18.27, S: 11.68.

6-(4-chlorophenyl)-5-imino-1,3-dimethyl-7-((3-(4-methylpiperazin-1-yl)propyl)amino)-5,6-dihydropyrimido[4,5-d]pyrimidine-2,4(1H,3H)-dithione (**4e**). IR (KBr) cm^−1^ 3159, 2938, 1624-. ^1^H NMR (CDCl_3_) δ 1.81–1.94 (m, 2H, CH_2_); 2.45–2.81 (m, 13H, 5×CH_2_N, CH_3_N); 3.57–3.69 (m, 2H, CH_2_N); 4.03–4.11 (s, 3H, CH_3_); 4.32–4.40 (s, 3H, CH_3_); 7.25–7.40 (m, 3H, 2 arom. H and NH); 7.59–7.70 (m, 3H, H arom.); 11.91 (bs, H, NH). ^13^C NMR (CDCl_3_) δ 24.8, 38.5, 41.4, 43.7, 45.4, 52.5, 54.0, 55.9, 101.3, 130.7, 131.5, 133.8, 136.3, 151.6, 152.8, 156.0, 175.6, 181.2. MS *m*/*z* 505.17 [M + H^+^]. Calcd. for C_22_H_29_ClN_8_S_2_, %: C: 52.31, H: 5.79, N: 22.18, S: 12.70. Found, %: C: 52.23, H: 6.16, N: 21.92, S: 12.47.

7-((2-(diethylamino)ethyl)amino)-1,3-diethyl-5-imino-6-phenyl-5,6-dihydropyrimido[4,5-d]pyrimidine-2,4(1H,3H)-dithione (**5a**). IR (KBr) cm^−1^ 3259, 3163, 1624. ^1^H NMR (CDCl_3_) δ 0.78–0.99 (m, 6H, 2×CH_3_); 1.32–1.58 (m, 6H, 2×CH_3_); 2.37–2.53 (m, 4H, 2×CH_2_N); 2.58–2.76 (m, 2H, CH_2_N); 3.40–3.50 (m, 2H, CH_2_N); 4.78–5.06 (m, 2H, CH_2_N); 5.15–45.59 (m, 2H, CH_2_N); 7.25–7.41 (m, 3H, 2 arom. H and NH); 7.50–7.70 (m, 3H, H arom.); 12.00 (bs, H, NH). ^13^C NMR (CDCl_3_) δ 11.1, 11.9, 39.1, 46.4, 49.9, 50.2, 101.3, 128.8, 129.8, 130.9, 135.7, 151.1, 152.6, 156.2, 174.4, 180.3. Calcd. for C_22_H_31_N_7_S_2_, %: C: 57.74, H: 6.83, N: 21.42, S: 14.01. Found, %: C: 57.60, H: 6.58, N: 21.28, S: 14.19.

1,3-diethyl-5-imino-6-phenyl-7-((2-(piperidin-1-yl)ethyl)amino)-5,6-dihydropyrimido[4,5-d]pyrimidine-2,4(1H,3H)-dithione (**5b**). IR (KBr) cm^−1^ 3177, 2933, 1627. ^1^H NMR (CDCl_3_) δ 1.25–1.52 (m, 12H, 2×CH_3_, 3×CH_2_); 2.20–2.35 (m, 2H, CH_2_N); 2.31–2.40 (m, 2H, CH_2_N); 3.40–3.50 (m, 2H, CH_2_N); 4.86–4.99 (m, 2H, CH_2_N); 5.19–5.52 (m, 2H, CH_2_N); 7.24–7.37 (m, 3H, 2 arom. H and NH); 7.51–7.70 (m, 3H, H arom.); 11.77 (bs, H, NH). ^13^C NMR (CDCl_3_) δ 11.1, 11.8, 23.7, 25.2, 38.7, 46.3, 49.9, 53.6, 55.6, 128.8, 130.0, 131.1, 135.8, 151.0, 152.2, 156.3, 174.4, 180.4. Calcd. for C_23_H_31_N_7_S_2_, %: C: 58.82, H: 6.65, N: 20.88, S: 13.65. Found, %: C: 58.61, H: 6.85, N: 20.88, S: 13.80.

7-((3-(dimethylamino)propyl)amino)-1,3-diethyl-5-imino-6-phenyl-5,6-dihydropyrimido[4,5-d]pyrimidine-2,4(1H,3H)-dithione (**5c**). IR (KBr) cm^−1^ 3188, 3038, 1618. ^1^H NMR (CDCl_3_) δ 1.37–1.51 (m, 6H, 2×CH_3_); 1.60–1.80 (m, 8H, 2×NCH_3_ and CH_2_); 2.19–2.40 (m, 2H, CH_2_); 3.42–3.61 (m, 2H, CH_2_); 4.79–5.00 (m, 2H, CH_2_); 5.09–5.50 (m, 2H, CH_2_); 7.15–7.33 (m, 3H, 2 arom. H and NH); 7.48–7.63 (m, 3H, H arom.); 11.73 (bs, H, NH). ^13^C NMR (CDCl_3_) δ 11.1, 11.8, 23.5, 31.1, 44.1, 44.9, 46.3, 49.7, 59.7, 101.7, 129.5, 129.7, 130.7, 136.4, 151.2, 153.2, 156.4, 174.3, 180.2. Calcd. for C_21_H_29_N_7_S_2_, %: C: 56.85, H: 6.59, N: 22.10, S: 14.46. Found, %: C: 57.19, H: 6.70, N: 22.28, S: 14.56.

7-((3-(dibutylamino)propyl)amino)-1,3-diethyl-5-imino-6-phenyl-5,6-dihydropyrimido[4,5-d]pyrimidine-2,4(1H,3H)-dithione (**5d**). IR (KBr) cm^−1^ 3187, 3062, 1621. ^1^H NMR (CDCl_3_) δ 0.82–1.0 (m, 6H, 2×CH_3_); 1.17–1.49 (m, 14H, 2×CH_3_ and 4×CH_2_); 1.61–1.74 (m, 2H, CH_2_); 2.05–2.22 (m, 4H, 2×CH_2_N); 2.45–2.58 (m, 2H, CH_2_N); 3.50–3.67 (m, 2H, CH_2_N); 4.82–4.99 (m, 2H, CH_2_N); 5.11–5.58 (m, 2H, CH_2_N); 7.20–7.36 (m, 3H, 2 arom. H and NH); 7.50–7.68 (m, 2H, H arom.); 11.85 (bs, H, NH). ^13^C NMR (CDCl_3_) δ 11.1, 11.9, 14.0, 20.6, 24.7, 26.9, 46.3, 50.0, 51.0, 53.7, 101.1, 129.1, 130.1, 131.1, 135.6, 151.1, 152.6, 156.7, 174.4, 180.4. MS *m*/*z* 528.29 [M + H^+^]. Calcd. for C_27_H_41_N_7_S_2_, %: C: 61.44, H: 7.83, N: 18.58, S: 12.15. Found, %: C: 61.59, H: 8.02, N: 18.35, S: 11.50.

1,3-diethyl-5-imino-7-((3-(4-methylpiperazin-1-yl)propyl)amino)-6-phenyl-5,6-dihydropyrimido[4,5-d]pyrimidine-2,4(1H,3H)-dithione (**5e**). IR (KBr) cm^−1^ 3365, 3187, 1674. ^1^H NMR (CDCl_3_) δ 1.30–1.58 (m, 6H, 2×CH_3_); 1.65–1.84 (m, 2H, CH_2_); 2.20–2.62 (m, 13H, 5×CH_2_N and CH_3_N); 3.43–3.62 (m, 2H, CH_2_N); 4.82–5.00 (m, 2H, CH_2_N); 5.12–5.55 (m, 2H, CH_2_N); 7.12–7.40 (m, 2H arom.); 7.50–7.75 (m, 4H, H arom and 1×NH); 11.80 (bs, H, NH). ^13^C NMR (CDCl_3_) δ 11.0, 12.0, 25.3, 40.9, 45.9, 46.3, 49.9, 52.9, 54.4, 55.5, 101.8, 129.1, 130.2, 131.1, 135.6, 151.1, 153.1, 156.1, 174.2, 180.4. Calcd. for C_24_H_34_N_8_S_2_, %: C: 57.80, H: 6.87, N: 22.47, S: 12.86. Found, %: C: 57.78, H: 6.50, N: 22.39, S: 12.73.

6-(4-chlorophenyl)-7-((2-(diethylamino)ethyl)amino)-1,3-diethyl-5-imino-5,6-dihydropyrimido[4,5-d]pyrimidine-2,4(1H,3H)-dithione (**6a**). IR (KBr) cm^−1^ 3132, 2965, 1620. ^1^H NMR (CDCl_3_) δ 0.71–0.90 (m, 6H, 2×CH_3_); 1.36–1.51 (m, 6H, 2×CH_3_); 2.29–2.43 (m, 4H, 2×CH_2_N); 2.51–2.59 (m, 2H, CH_2_N); 3.37–3.48 (m, 2H, CH_2_N); 4.86–4.99 (m, 2H, CH_2_N); 5.19–5.52 (m, 2H, CH_2_N); 7.19–7.30 (m, 3H, 2 arom. H and NH); 7.52–7.63 (m, 3H, H arom.); 11.77 (bs, H, NH). ^13^C NMR (CDCl_3_) δ 11.0, 11.9, 12.1, 38.9, 46.3, 49.9, 50.2, 101.7, 130.4, 131.1, 134.2, 135.9, 151.2, 152.7, 155.9, 174.3, 180.5. Calcd. for C_22_H_30_ClN_7_S_2_, %: C: 53.70, H: 6.14, N: 19.92, S: 13.03. Found, %: C: 53.86, H: 6.17, N: 19.72, S: 13.35.

6-(4-chlorophenyl)-1,3-diethyl-5-imino-7-((2-(piperidin-1-yl)ethyl)amino)-5,6-dihydropyrimido[4,5-d]pyrimidine-2,4(1H,3H)-dithione (**6b**). IR (KBr) cm^−1^ 3166, 2977, 1626. ^1^H NMR (CDCl_3_) δ 1.30–1.52 (m, 12H, 2×CH_3_, 3×CH_2_); 2.23–2.39 (m, 2H, CH_2_N); 2.42–2.53 (m, 2H, CH_2_N); 3.40–3.51 (m, 2H, CH_2_N); 4.85–4.99 (m, 2H, CH_2_N); 5.18–5.49 (m, 2H, CH_2_N); 7.22–7.33 (m, 3H, 2 arom. H and NH); 7.57–7.67 (m, 3H, H arom.); 11.79 (bs, H, NH). ^13^C NMR (CDCl_3_) δ 11.0, 11.9, 23.8, 25.4, 38.5, 46.3, 49.9, 53.7, 55.4, 130.4, 131.2, 134.1, 136.1, 151.3, 156.1, 174.2, 180.4. Calcd. for C_23_H_30_ClN_7_S_2_, %: C: 54.80, H: 6.00, N: 19.45, S: 12.72. Found, %: C: 55.18, H: 6.07, N: 19.31, S: 13.01.

6-(4-chlorophenyl)-7-((3-(dimethylamino)propyl)amino)-1,3-diethyl-5-imino-5,6-dihydropyrimido[4,5-d]pyrimidine-2,4(1H,3H)-dithione (**6c**). IR (KBr) cm^−1^ 3442, 3181, 1618. ^1^H NMR (CDCl_3_) δ 1.38–1.49 (m, 6H, 2×CH_3_); 1.62–1.84 (m, 8H, 2×NCH_3_ and CH_2_); 2.32–2.41 (m, 2H, CH_2_N); 3.51–3.60 (m, 2H, CH_2_N); 4.83–4.92 (m, 2H, CH_2_N); 5.09–5.60 (m, 2H, CH_2_N); 7.20–7.30 (m, 3H, 2 arom. H and NH); 7.52–7.61 (m, 2H, H arom.); 11.70 (bs, H, NH).^13^C NMR (CDCl_3_) δ 11.0, 11.8, 23.4, 44.1, 45.1, 46.3, 49.9, 59.8, 101.6, 130.7, 131.0, 134.9, 135.6, 151.2, 153.0, 156.3, 174.3, 180.3. MS *m*/*z* 478.2 [M + H^+^]. Calcd. for C_21_H_28_ClN_7_S_2_, %: C: 52.76, H: 5.90, N: 20.51, S: 13.41. Found, %: C: 52.49, H: 6.12, N: 20.50, S: 13.09.

6-(4-chlorophenyl)-7-((3-(dibutylamino)propyl)amino)-1,3-diethyl-5-imino-5,6-dihydropyrimido[4,5-d]pyrimidine-2,4(1H,3H)-dithione (**6d**). IR (KBr) cm^−1^ 3186, 3051, 1620. ^1^H NMR (CDCl_3_) δ 0.88–1.02 (m, 6H, 2×CH_3_); 1.21–1.48 (m, 14H, 2×CH_3_ and 4×CH_2_); 1.65–1.81 (m, 2H, CH_2_); 2.20–238 (m, 4H, 2×CH_2_N); 2.53–2.67 (m, 2H, CH_2_N); 3.54–3.62 (m, 2H, CH_2_N); 4.79–4.98 (m, 2H, CH_2_N); 5.10–5.54 (m, 2H, CH_2_N); 7.16–7.24 (m, 3H, 2 arom. H and NH); 7.51–7.63 (m, 2H, H arom.); 11.82 (bs, H, NH). ^13^C NMR (CDCl_3_) δ 11.0, 11.9, 14.0, 20.7, 24.5, 26.9, 46.3, 49.9, 51.2, 53.8, 101.6, 130.7, 131.2, 134.3, 136.1, 151.0, 152.5, 156.5, 174.3, 180.3. Calcd. for C_27_H_40_ClN_7_S_2_, %: C: 57.68, H: 7.17, N: 17.44, S: 11.41. Found, %: C: 57.75, H: 7.26, N: 17.36, S: 11.01.

6-(4-chlorophenyl)-1,3-diethyl-5-imino-7-((3-(4-methylpiperazin-1-yl)propyl)amino)-5,6-dihydropyrimido[4,5-d]pyrimidine-2,4(1H,3H)-dithione (**6e**). IR (KBr) cm^−1^ 3166, 3060, 1622. ^1^H NMR (CDCl_3_) δ 1.19–1.50 (m, 6H, 2×CH_3_); 1.66–1.82 (m, 2H, CH_2_); 2.28–2.58 (m, 13H, CH_3_, 5×CH_2_); 3.47–3.60 (m, 2H, CH_2_N); 4.82–4.95 (m, 2H, CH_2_); 5.18–5.60 (m, 2H, CH_2_); 7.21–7.34 (m, 3H, 2 arom. H and NH); 7.57–7.67 (m, 3H, H arom.); 11.74 (bs, H, NH). ^13^C NMR (CDCl_3_) δ 10.9, 12.0, 25.0, 41.3, 45.4, 46.4, 50.0, 52.5, 54.0, 55.8, 101.7, 130.7, 131.5, 133.8, 136.4, 151.0, 152.8, 156.2, 174.2, 180.5. Calcd. for C_24_H_33_ClN_8_S_2_, %: C: 54.07, H: 6.24, N: 21.02, S: 12.03. Found, %: C: 54.25, H: 6.45, N: 21.20, S: 11.83.

### 3.2. Biology

#### 3.2.1. MTT Assay

MTT assay was performed using SKOV-3 (ovarian adenocarcinoma, ATCC, Manassas, VA, USA) and MCF-7 (breast adenocarcinoma, Biologic Bank and Cell Factory, IRCCS Policlinico San Martino, Genoa, Italy) cell lines. Both cell lines were grown in DMEM (with 10% FBS, 2 mM Glutamine and 1% penstrep. All reagents were purchased from EuroClone, Milan, Italy) and incubated at 37 °C in 5% CO_2_ in a humidified environment. Briefly, the neoplastic cells were plated in 96 well plates at an adequate number to reach 80–85% of confluence at the end of the assay. 16 h after plating, compounds were dissolved in DMSO to give a 10 mM stock solution, diluted in growth medium and added at a final concentration of 10 μM. After 48 h of incubation, 30 μL of MTT (3-(4,5-dimethyl-2-thiazolyl)-2,5-diphenyl-2H-tetrazolium bromide) at a concentration of 2 mg/mL in PBS, were added in each well. Then, after further 4 h of incubation, the sur-natant was removed and 100 μL/well of DMSO were used to dissolve the Formazan precipitate that can be found in vital cells. After 20 min, the results were read at 570 nm by means of a spectrophotometer. The results are expressed as percentage of the control samples in which the cells were incubated with the same amount of DMSO but without compounds. The assays were repeated three times. In each set, every single compound was tested six times. Means and standard deviations were calculated.

#### 3.2.2. Annexin V & Dead Cell Assay

Muse™ Annexin V & Dead Cell Assay was performed following the manufacturer instruction (Millipore, Billerica, MA, USA). Briefly, cultured cells were treated for 48 h with 2.5 μM compound **5d** (final concentration in growth medium). Once dissociated with trypsin, the cells were resuspended in DMEM containing 10% FBS, 2 mM Glutamine and 1% pen-strep to achieve a final cell concentration of 1 × 10^5^–1 × 10^7^ cell/mL. Then, 100 μL of the cell suspension were mixed with 100 μL of Muse™ Annexin V & Dead Cell reagent. After a 20 min incubation in the dark, the sample was read using the Muse™ Cell Analyser. The control sample was treated in the same way as the **5d** compound treated sample.

### 3.3. Docking Simulation

The molecular structures of compounds **1d** and **5d** were built by MOE2009.10 (builder module), parametrized by MMFF94x forcefield and their docking poses within TopoIIα were calculated by Autodock 4.2 [20]. After the removal of water molecules from the crystal structure of the TopoIIα-DNA complex (PDB code 4FM9) [22], polar hydrogen and Gasteiger-Huckel charges were added. The missing residues in the pdb file (i.e., 431–432, 1093–1123 and 1191–1193) were located in protein regions far from the etoposide binding site (vide infra) and therefore were not processed. The ligands root was defined automatically. The tertiary amine groups of the two ligands were considered in the protonated form as suggested by the calculated pKa values. A 60 × 60 × 60 Å grid (grid spacing 0.375 Å) was centered in the binding site of etoposide (as defined in the pdb file 3QX3) [23] and electrostatic and affinity maps for each atom type of the ligand were calculated. The docking search was performed over 100 conformers using the Genetic Algorithm Local Search protocol as implemented in Autodock (population size: 50; rate of gene mutation: 0.02; rate of crossover: 0.8). The docking poses were clustered (rmsd: 2.0 Å) and the best conformation of the low energy highest populated cluster was selected as the binding conformation. Models analysis was carried out using the CCP4 program suite [24]. The calculations were run on a Linux PC (Intel^®^ processor Core™ i7-2600 CPU@3.40 GHz).

## 4. Conclusions

**Figure 5:**
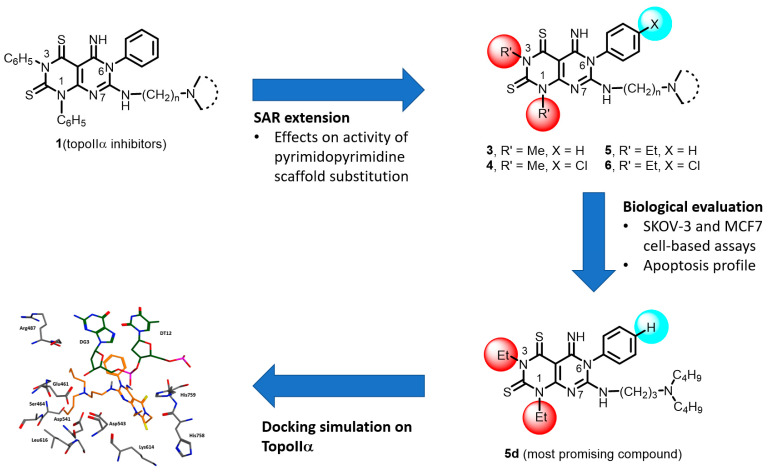
Design, synthesis, biological evaluation and docking simulation of new pyrimido-pyrimidine **3**–**6**.

Figure 5 summarizes the rationale of the current work, highlighting the key steps described in the manuscript. In particular, the synthesis of compounds **3**–**6** represents an efficient strategy for the preparation of pyrimido-pyrimidine derivatives bearing different substituents at position 1, 3 and 6. Chemical accessibility to such compounds allowed the extension of the SARs on this series of rigid analogues of merbarone. The cytotoxic effect of compounds **3–6** tested on SKOV-3 and MCF-7 cell line emerged to be influenced by both the nature of the basic chain and the substituents at positions 1, 3 and 6 of the pyrimido-pyrimidine scaffold. Compounds **5d**,**e** proved to be the most effective derivatives still showing a reduced potency in comparison with the 1,3,6-triphenyl substituted compound **1d**. Furthermore, as other DNA Topoisomerase II inhibitors, **5d** compound induced programmed cell death in cultured cell lines. According to docking simulations, **5d** would bind TopoIIα in the etoposide binding site, inhibiting the enzyme activity and leading to SKOV-3 apoptosis.

## Figures and Tables

**Table 1:** Reaction conditions, yields and melting points of compounds **3**–**6**.

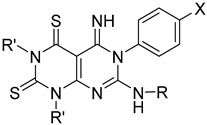						
Cpd	R′	X	R	Reaction Conditions	Yields (%)	mp (°C)
**3a**	Me	H	–CH_2_CH_2_NEt_2_	80 °C, 4 h	86	204–205
**3b**	Me	H	–CH_2_CH_2_-Pip	80 °C, 4 h	54	211–213
**3c**	Me	H	–CH_2_CH_2_CH_2_NMe_2_	rt, 16 h	90	214–215
**3d**	Me	H	–CH_2_CH_2_CH_2_N(nBu)_2_	rt, 16 h	51	184–185
**3e**	Me	H	–CH_2_CH_2_CH_2_N(CH_2_CH_2_)_2_NMe	rt, 16 h	51	175–177
**4a**	Me	Cl	–CH_2_CH_2_NEt_2_	80 °C, 4 h	54	229–231
**4b**	Me	Cl	–CH_2_CH_2_-Pip	80 °C, 4 h	38	237–239
**4c**	Me	Cl	–CH_2_CH_2_CH_2_NMe_2_	rt, 16 h	69	232–234
**4d**	Me	Cl	–CH_2_CH_2_CH_2_N(nBu)_2_	rt, 16 h	53	184–185
**4e**	Me	Cl	–CH_2_CH_2_CH_2_N(CH_2_CH_2_)_2_NMe	80 °C, 4 h	38	125–126
**5a**	Et	H	–CH_2_CH_2_NEt_2_	rt, 16 h	66	186–188
**5b**	Et	H	–CH_2_CH_2_-Pip	80 °C, 2 h	79	196–198
**5c**	Et	H	–CH_2_CH_2_CH_2_NMe_2_	rt, 16 h	59	191–193
**5d**	Et	H	–CH_2_CH_2_CH_2_N(nBu)_2_	rt, 16 h	23	88–90
**5e**	Et	H	–CH_2_CH_2_CH_2_N(CH_2_CH_2_)_2_NMe	60 °C, 2 h	28	163–165
**6a**	Et	Cl	–CH_2_CH_2_NEt_2_	100 °C, 5 min μwave	51	167–169
**6b**	Et	Cl	–CH_2_CH_2_-Pip	80 °C, 4 h	60	210–212
**6c**	Et	Cl	–CH_2_CH_2_CH_2_NMe_2_	rt, 16 h	90	209–211
**6d**	Et	Cl	–CH_2_CH_2_CH_2_N(nBu)_2_	rt, 16 h	68	150–152
**6e**	Et	Cl	–CH_2_CH_2_CH_2_N(CH_2_CH_2_)_2_NMe	100 °C, 5 min μwave	70	183–184

**Table 2:** Growth percent values of SKOV-3 and MCF-7 cells in the presence of derivatives **3**–**6** at the concentration of 10 μM.

	Growth Percent			Growth Percent	
Cpd	SKOV-3	MCF-7	Cpd	SKOV-3	MCF-7
**3a**	60.65	14.08	5a	4.15	3.13
**3b**	63.28	13.58	5b	24.89	−0.40
**3c**	−37.72	−7.26	5c	−7.94	2.40
**3d**	−32.17	−12.78	5d	−76.50	−33.72
**3e**	8.84	12.45	5e	−32.58	−55.88
**4a**	48.52	19.77	6a	39.32	8.06
**4b**	58.62	21.47	6b	28.02	3.26
**4c**	34.70	20.94	6c	−4.85	−10.89
**4d**	11.96	6.29	6d	1.13	−24.00
**4e**	17.12	10.25	6e	−39.38	−27.93
**1d**	−86.70	−83.51	1d	−86.70	−83.51

## Data Availability

The data presented in this study are available in this article.

## Supplementary Materials

Figure S1: ^1^H-NMR (200 MHz, d_6_-DMSO) spectrum of compound **7**, Figure S2: ^13^C-NMR (101 MHz, d_6_-DMSO) spectrum of compound **7**, **Figure S3**: IR (KBr) spectrum of compound **7**, Figure S4: Mass spectrum of compound **7**, Figure S5: ^1^H-NMR (200 MHz, d_6_-DMSO) spectrum of compound **8**, Figure S6: ^13^C-NMR (101 MHz, d_6_-DMSO) spectrum of compound **8**, Figure S7: IR (KBr) spectrum of compound **8**, Figure S8: Mass spectrum of compound **8**, Figure S9: ^1^H-NMR (200 MHz, CDCl_3_) spectrum of compound **9**, Figure S10: ^13^C-NMR (101 MHz, CDCl_3_) spectrum of compound **9**, Figure S11: IR (KBr) spectrum of compound **9**, Figure S12: Mass spectrum of compound **9**, Figure S13: ^1^H-NMR (200 MHz, CDCl_3_) spectrum of compound **10**, Figure S14: ^13^C-NMR (101 MHz, CDCl_3_) spectrum of compound **10**, Figure S15: IR (KBr) spectrum of compound **10**, Figure S16: Mass spectrum of compound **10**, Figure S17: ^1^H-NMR (300 MHz, d_6_-DMSO) spectrum of compound **11**, Figure S18: ^13^C-NMR (75 MHz, d_6_-DMSO) spectrum of compound **11**, Figure S19: IR (KBr) spectrum of compound **11**, Figure S20: Mass spectrum of compound **11**, Figure S21: ^1^H-NMR (200 MHz, CDCl_3_) spectrum of compound **12**, Figure S22: ^13^C-NMR (101 MHz, CDCl_3_) spectrum of compound **12**, Figure S23: IR (KBr) spectrum of compound **12**, Figure S24: Mass spectrum of compound **12**, Figure S25: ^1^H-NMR (200 MHz, CDCl_3_) spectrum of compound **3a**, Figure S26: ^13^C-NMR (101 MHz, CDCl_3_) spectrum of compound **3a**, Figure S27: IR (KBr) spectrum of compound **3a**, Figure S28: Mass spectrum of compound **3a**, Figure S29: ^1^H-NMR (200 MHz, CDCl_3_) spectrum of compound **3b**, Figure S30: ^13^C-NMR (101 MHz, CDCl_3_) spectrum of compound **3b**, Figure S31: IR (KBr) spectrum of compound **3b**, Figure S32: ^1^H-NMR (200 MHz, CDCl_3_) spectrum of compound **3c**, Figure S33: ^13^C-NMR (101 MHz, CDCl_3_) spectrum of compound **3c**, Figure S34: IR (KBr) spectrum of compound **3c**, Figure S35: ^1^H-NMR (200 MHz, CDCl_3_) spectrum of compound **3d**, Figure S36: ^13^C-NMR (101 MHz, CDCl_3_) spectrum of compound **3d**, Figure S37: IR (KBr) spectrum of compound **3d**, Figure S38: ^1^H-NMR (200 MHz, CDCl_3_) spectrum of compound **3e**, Figure S39: ^13^C-NMR (101 MHz, CDCl_3_) spectrum of compound **3e**, Figure S40: IR (KBr) spectrum of compound **3e**, Figure S41: ^1^H-NMR (200 MHz, CDCl_3_) spectrum of compound **4a**, Figure S42: ^13^C-NMR (101 MHz, CDCl_3_) spectrum of compound **4a**, Figure S43: IR (KBr) spectrum of compound **4a**, Figure S44: ^1^H-NMR (200 MHz, CDCl_3_) spectrum of compound **4b**, Figure S45: ^13^C-NMR (101 MHz, CDCl_3_) spectrum of compound **4b**, Figure S46: IR (KBr) spectrum of compound **4b**, Figure S47: ^1^H-NMR (200 MHz, CDCl_3_) spectrum of compound **4c**, Figure S48: ^13^C-NMR (101 MHz, CDCl_3_) spectrum of compound **4c,** Figure S49: IR (KBr) spectrum of compound **4c**, Figure S50: ^1^H-NMR (200 MHz, CDCl_3_) spectrum of compound **4d**, Figure S51: ^13^C-NMR (101 MHz, CDCl_3_) spectrum of compound **4d**, Figure S52: IR (KBr) spectrum of compound **4d**, Figure S53: ^1^H-NMR (200 MHz, CDCl_3_) spectrum of compound **4e**, Figure S54: ^13^C-NMR (101 MHz, CDCl_3_) spectrum of compound **4e**, Figure S55: IR (KBr) spectrum of compound **4e**, Figure S56: Mass spectrum of compound **4e**, Figure S57: ^1^H-NMR (200 MHz, CDCl_3_) spectrum of compound **5a**, Figure S58: ^13^C-NMR (101 MHz, CDCl_3_) spectrum of compound **5a**, Figure S59: IR (KBr) spectrum of compound **5a**, Figure S60: ^1^H-NMR (200 MHz, CDCl_3_) spectrum of compound **5b**, Figure S61: ^13^C-NMR (101 MHz, CDCl_3_) spectrum of compound **5b**, Figure S62: IR (KBr) spectrum of compound **5b**, Figure S63: ^1^H-NMR (200 MHz, CDCl_3_) spectrum of compound **5c**, Figure S64: ^13^C-NMR (101 MHz, CDCl_3_) spectrum of compound **5c**, Figure S65: IR (KBr) spectrum of compound **5c**, Figure S66: ^1^H-NMR (200 MHz, CDCl_3_) spectrum of compound **5d**, Figure S67: ^13^C-NMR (101 MHz, CDCl_3_) spectrum of compound **5d**, Figure S68: IR (KBr) spectrum of compound **5d**, Figure S69: Mass spectrum of compound **5d**, Figure S70: ^1^H-NMR (200 MHz, CDCl_3_) spectrum of compound **5e**, S71: ^13^C-NMR (101 MHz, CDCl_3_) spectrum of compound **5e**, Figure S72: IR (KBr) spectrum of compound **5e**, Figure S73: ^1^H-NMR (200 MHz, CDCl_3_) spectrum of compound **6a**, Figure S74: ^13^C-NMR (101 MHz, CDCl_3_) spectrum of compound **6a**, Figure S75: IR (KBr) spectrum of compound **6a**, Figure S76: ^1^H-NMR (200 MHz, CDCl_3_) spectrum of compound **6b**, Figure S77: ^13^C-NMR (101 MHz, CDCl_3_) spectrum of compound **6b**, Figure S78: IR (KBr) spectrum of compound **6b**, Figure S79: ^1^H-NMR (200 MHz, CDCl_3_) spectrum of compound **6c**, Figure S80: ^13^C-NMR (101 MHz, CDCl_3_) spectrum of compound **6c**, Figure S81: IR (KBr) spectrum of compound **6c**, Figure S82: Mass spectrum of compound **6c**, Figure S83: ^1^H-NMR (200 MHz, CDCl_3_) spectrum of compound **6d**, Figure S84: ^13^C-NMR (101 MHz, CDCl_3_) spectrum of compound **6d**, Figure S85: IR (KBr) spectrum of compound **6d**, Figure S86: ^1^H-NMR (200 MHz, CDCl_3_) spectrum of compound **6e**, Figure S87: ^13^C-NMR (101 MHz, CDCl_3_) spectrum of compound **6e**, Figure S88. IR (KBr) spectrum of compound **6e**.

## Sample Availability

Samples of the synthesized compounds are available from the authors.

## References

[1] Wang J.C. (1996). DNA topoisomerases. Annu. Rev. Biochem..

[2] Champoux J.J. (2001). DNA topoisomerases: Structure, function, and mechanism. Annu. Rev. Biochem..

[3] Stewart L., Redinbo M.R., Qiu X., Hol W.G.J., Champoux J.J. (1998). A model for the mechanism of human topoisomerase I. Science.

[4] Wang J.C. (1998). Moving one DNA double helix through another by a type II DNA topoisomerase: The story of a simple molecular machine. Q. Rev. Biophys..

[5] Hu W., Huang X.S., Wu J.F., Yang L., Zheng Y.T., Shen Y.M., Li Z.Y., Li X. (2018). Discovery of Novel Topoisomerase II Inhibitors by Medicinal Chemistry Approaches. J. Med. Chem..

[6] Liang X., Wu Q., Luan S., Yin Z., He C., Yin L., Zou Y., Yuan Z., Li L., Song X. (2019). A Comprehensive Review of Topoisomerase Inhibitors as Anticancer Agents in the Past Decade. Eur. J. Med. Chem..

[7] Wang W., Tse-Dinh Y.C. (2019). Recent Advances in Use of Topoisomerase Inhibitors in Combination Cancer Therapy. Curr. Top. Med. Chem..

[8] Pogorelcnik B., Perdih A., Solmajer T. (2013). Recent Advances in the Development of Catalytic Inhibitors of Human DNA Topoisomerase IIalpha as Novel Anticancer Agents. Curr. Med. Chem..

[9] Riddell I.A., Agama K., Park G.Y., Pommier Y., Lippard S.J. (2016). Phenanthriplatin Acts as a Covalent Poison of Topoisomerase II Cleavage Complexes. ACS Chem. Biol..

[10] Larsen A.K., Escargueil A.E., Skladanowski A. (2003). Catalytic Topoisomerase II Inhibitors in Cancer Therapy. Pharmacol. Ther..

[11] Andoh T., Ishida R. (1998). Catalytic inhibitors of DNA topoisomerase II. Biochim. Biophys. Acta.

[12] Fortune J.M., Osheroff N. (1998). Merbarone Inhibits the Catalytic Activity of Human Topoisomerase IIalpha by Blocking DNA Cleavage. J. Biol. Chem..

[13] Dimaggio J.J., Warrell R.P., Muindi J., Stevens Y.W., Lee S.J., Lowenthal D.A., Haines I., Walsh T.D., Baltzer L., Yaldaei S. (1990). Phase I Clinical and Pharmacological Study of Merbarone. Cancer Res..

[14] Malik U.R., Dutcher J.P., Caliendo G., Lasala P., Mitnick R., Wiernik P.H. (1997). Phase II Trial of Merbarone in Patients with Malignant Brain Tumors. Med. Oncol..

[15] Ranise A., Spallarossa A., Schenone S., Bruno O., Bondavalli F., Pani A., Marongiu M.E., Mascia V., La Colla P., Loddo R. (2003). Synthesis and antiproliferative activity of basic thioanalogues of merbarone. Bioorg. Med. Chem..

[16] Spallarossa A., Rotolo C., Sissi C., Marson G., Greco M.L., Ranise A., La Colla P., Busonera B., Loddo R. (2013). Further SAR studies on bicyclic basic merbarone analogues as potent antiproliferative agents. Bioorg. Med. Chem..

[17] Arencibia J.M., Brindani N., Franco-Ulloa S., Nigro M., Kuriappan J.A., Ottonello G., Bertozzi S.M., Summa M., Girotto S., Bertorelli R. (2020). Design, synthesis, dynamic docking, biochemical characterization, and in vivo pharmacokinetics studies of novel topoisomerase II poisons with promising antiproliferative activity. J. Med. Chem..

[18] Ortega J.A., Riccardi L., Minniti E., Borgogno M., Arencibia J.M., Greco M.L., Minarini A., Sissi C., De Vivo M. (2018). Pharmacophore Hybridization to Discover Novel Topoisomerase II Poisons with Promising Antiproliferative Activity. J. Med. Chem..

[19] Negri C., Bemardi R., Donzelli M., Scovassi A.I. (1995). Induction of apoptotic cell death by DNA topoisomerase II inhibitors. Biochimie.

[20] Morris G.M., Huey R., Lindstrom W., Sanner M.F., Belew R.K., Goodsell D.S., Olson A.J. (2009). Autodock4 and AutoDockTools4: Automated docking with selective receptor flexibility. J. Comput. Chem..

[21] Tshepelevitsh S., Kütt A., Lõkov M., Kaljurand I., Saame J., Heering A., Plieger P.G., Vianello R., Leito I. (2019). On the basicity of organic bases in different media. Eur. J. Org. Chem..

[22] Wendorff T.J., Schmidt B.H., Heslop P., Austin C.A., Berger J.M. (2012). The Structure of DNA-Bound Human Topoisomerase II Alpha: Conformational Mechanisms for Coordinating Inter-Subunit Interactions with DNA Cleavage. J. Mol. Biol..

[23] Wu C.C., Li T.K., Farh L., Lin L.Y., Lin T.S., Yu Y.J., Yen T.J., Chiang C.W., Chan N.L. (2011). Structural basis of type II topoisomerase inhibition by the anticancer drug etoposide. Science.

[24] Winn M.D., Ballard C.C., Cowtan K.D., Dodson E.J., Emsley P., Evans P.R., Keegan R.M., Krissinel E.B., Leslie A.G.W., McCoy A. (2011). Overview of the CCP4 suite and current developments. Acta Cryst..

