# Supplementary material for: Bicyclic Basic Merbarone Analogues as Antiproliferative Agents
Source: Molecules. 2021 Jan 21;26(3):557. doi: 10.3390/molecules26030557 (PMC7866144; doi:10.3390/molecules26030557)

Supplementary information

Bicyclic basic merbarone analogues as antiproliferative agents

Andrea Spallarossa[1,*], Matteo Lusardi[1], Chiara Caneva[1], Aldo Profumo[2], Camillo Rosano[2] and Marco Ponassi[2]

[1]Department of Pharmacy, University of Genova, Genova, viale Benedetto XV, 3, 16132, Italy; andrea.spallarossa@ unige.it (AS); matteo.lusardi@edu.unige.it (ML); chiara.caneva.cc@gmail.com (CC).

[2]IRCCS Ospedale Policlinico San Martino, Proteomics and Mass Spectrometry Unit, L.go. R. Benzi, 10, Genova, 16132, Italy; aldo.profumo@hsanmartino.it (AP); camillo.rosano@hsanmartino.it (CR); marco.ponassi@hsanmartino.it (MP).

Contents

Figure S1. ^{1}H-NMR (200 MHz, d_6-DMSO) spectrum of compound **7**
Figure S2. ^{13}C-NMR (101 MHz, d_6-DMSO) spectrum of compound **7**
Figure S3. IR (KBr) spectrum of compound **7**
Figure S4. Mass spectrum of compound **7**
Figure S5. ^{1}H-NMR (200 MHz, d_6-DMSO) spectrum of compound **8**
Figure S6. ^{13}C-NMR (101 MHz, d_6-DMSO) spectrum of compound **8**
Figure S7. IR (KBr) spectrum of compound **8**
Figure S8. Mass Spectrum of Compound **8**
Figure S9. ^{1}H-NMR (200 MHz, $CDCl_3$) spectrum of compound **9**
Figure S10. ^{13}C-NMR (101 MHz, $CDCl_3$) spectrum of compound **9**
Figure S11. IR (KBr) spectrum of compound **9**
Figure S12. Mass spectrum of compound **9**
Figure S13. ^{1}H-NMR (200 MHz, $CDCl_3$) spectrum of compound **10**
Figure S14. ^{13}C-NMR (101 MHz, $CDCl_3$) spectrum of compound **10**
Figure S15. IR (KBr) spectrum of compound **10**
Figure S16. Mass spectrum of compound **10**
Figure S17. ^{1}H-NMR (300 MHz, d_6-DMSO) spectrum of compound **11**
Figure S18. ^{13}C-NMR (75 MHz, d_6-DMSO) spectrum of compound **11**
Figure S19. IR (KBr) spectrum of compound **11**
Figure S20. Mass spectrum of compound **11**
Figure S21. ^{1}H-NMR (200 MHz, $CDCl_3$) spectrum of compound **12**
Figure S22. ^{13}C-NMR (101 MHz, $CDCl_3$) spectrum of compound **12**
Figure S23. IR (KBr) spectrum of compound **12**
Figure S24. Mass spectrum of compound **12**

Figure S25. ^{1}H-NMR (200 MHz, $CDCl_3$) spectrum of compound **3a**
Figure S26. ^{13}C-NMR (101 MHz, $CDCl_3$) spectrum of compound **3a**
Figure S27. IR spectrum of compound **3a**
Figure S28. Mass spectrum of compound **3a**
Figure S29. ^{1}H-NMR (200 MHz, $CDCl_3$) spectrum of compound **3b**
Figure S30. ^{13}C-NMR (101 MHz, $CDCl_3$) spectrum of compound **3b**
Figure S31. IR (KBr) spectrum of compound **3b**
Figure S32. ^{1}H-NMR (200 MHz, $CDCl_3$) spectrum of compound **3c**
Figure S33. ^{13}C-NMR (101 MHz, $CDCl_3$) spectrum of compound **3c**
Figure S34. IR (KBr) spectrum of compound **3c**
Figure S35. ^{1}H-NMR (200 MHz, $CDCl_3$) spectrum of compound **3d**
Figure S36. ^{13}C-NMR (101 MHz, $CDCl_3$) spectrum of compound **3d**
Figure S37. IR (KBr) spectrum of compound **3d**
Figure S38. ^{1}H-NMR (200 MHz, $CDCl_3$) spectrum of compound **3e**
Figure S39. ^{13}C-NMR (101 MHz, $CDCl_3$) spectrum of compound **3e**
Figure S40. IR (KBr) spectrum of compound **3e**
Figure S41. ^{1}H-NMR (200 MHz, $CDCl_3$) spectrum of compound **4a**
Figure S42. ^{13}C-NMR (101 MHz, $CDCl_3$) spectrum of compound **4a**
Figure S43. IR (KBr) spectrum of compound **4a**
Figure S44. ^{1}H-NMR (200 MHz, $CDCl_3$) spectrum of compound **4b**
Figure S45. ^{13}C-NMR (101 MHz, $CDCl_3$) spectrum of compound **4b**
Figure S46. IR (KBr) spectrum of compound **4b**
Figure S47. ^{1}H-NMR (200 MHz, $CDCl_3$) spectrum of compound **4c**
Figure S48. ^{13}C-NMR (101 MHz, $CDCl_3$) spectrum of compound **4c**
Figure S49. IR (KBr) spectrum of compound **4c**
Figure S50. ^{1}H-NMR (200 MHz, $CDCl_3$) spectrum of compound **4d**
Figure S51. ^{13}C-NMR (101 MHz, $CDCl_3$) spectrum of compound **4d**
Figure S52. IR (KBr) spectrum of compound **4d**
Figure S53. ^{1}H-NMR (200 MHz, $CDCl_3$) spectrum of compound **4e**
Figure S54. ^{13}C-NMR (101 MHz, $CDCl_3$) spectrum of compound **4e**
Figure S55. IR (KBr) spectrum of compound **4e**
Figure S56. Mass spectrum of compound **4e**
Figure S57. ^{1}H-NMR (200 MHz, $CDCl_3$) spectrum of compound **5a**
Figure S58. ^{13}C-NMR (101 MHz, $CDCl_3$) spectrum of compound **5a**
Figure S59. IR (KBr) spectrum of compound **5a**
Figure S60. ^{1}H-NMR (200 MHz, $CDCl_3$) spectrum of compound **5b**
Figure S61. ^{13}C-NMR (101 MHz, $CDCl_3$) spectrum of compound **5b**
Figure S62. IR (KBr) spectrum of compound **5b**
Figure S63. ^{1}H-NMR (200 MHz, $CDCl_3$) spectrum of compound **5c**
Figure S64. ^{13}C-NMR (101 MHz, $CDCl_3$) spectrum of compound **5c**
Figure S65. IR (KBr) spectrum of compound **5c**
Figure S66. ^{1}H-NMR (200 MHz, $CDCl_3$) spectrum of compound **5d**
Figure S67. ^{13}C-NMR (101 MHz, $CDCl_3$) spectrum of compound **5d**
Figure S68. IR (KBr) spectrum of compound **5d**
Figure S69. Mass spectrum of compound **5d**
Figure S70. ^{1}H-NMR (200 MHz, $CDCl_3$) spectrum of compound **5e**
Figure S71. ^{13}C-NMR (101 MHz, $CDCl_3$) spectrum of compound **5e**
Figure S72. IR (KBr) spectrum of compound **5e**
Figure S73. ^{1}H-NMR (200 MHz, $CDCl_3$) spectrum of compound **6a**
Figure S74. ^{13}C-NMR (101 MHz, $CDCl_3$) spectrum of compound **6a**
Figure S75. IR (KBr) spectrum of compound **6a**

Figure S76. ^{1}H-NMR (200 MHz, $CDCl_3$) spectrum of compound **6b**
Figure S77. ^{13}C-NMR (101 MHz, $CDCl_3$) spectrum of compound **6b**
Figure S78. IR (KBr) spectrum of compound **6b**
Figure S79. ^{1}H-NMR (200 MHz, $CDCl_3$) spectrum of compound **6c**
Figure S80. ^{13}C-NMR (101 MHz, $CDCl_3$) spectrum of compound **6c**
Figure S81. IR (KBr) spectrum of compound **6c**
Figure S82. Mass spectrum of compound **6c**
Figure S83. ^{1}H-NMR (200 MHz, $CDCl_3$) spectrum of compound **6d**
Figure S84. ^{13}C-NMR (101 MHz, $CDCl_3$) spectrum of compound **6d**
Figure S85. IR (KBr) spectrum of compound **6d**
Figure S86. ^{1}H-NMR (200 MHz, $CDCl_3$) spectrum of compound **6e**
Figure S87. ^{13}C-NMR (101 MHz, $CDCl_3$) spectrum of compound **6e**
Figure S88. IR (KBr) spectrum of compound **6e**

Figure S1. ^{1}H-NMR (200 MHz, d_6-DMSO) spectrum of compound **7**

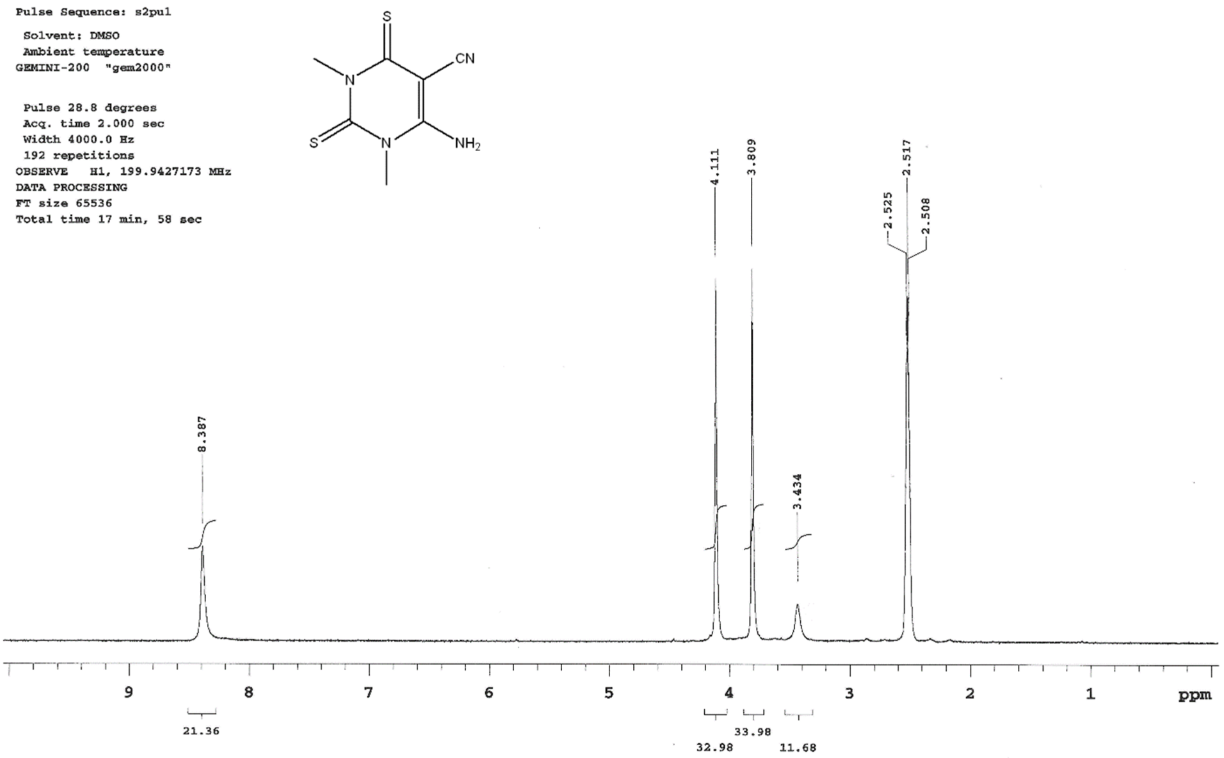

Figure S2. ^{13}C-NMR (101 MHz, d_6-DMSO) spectrum of compound **7**

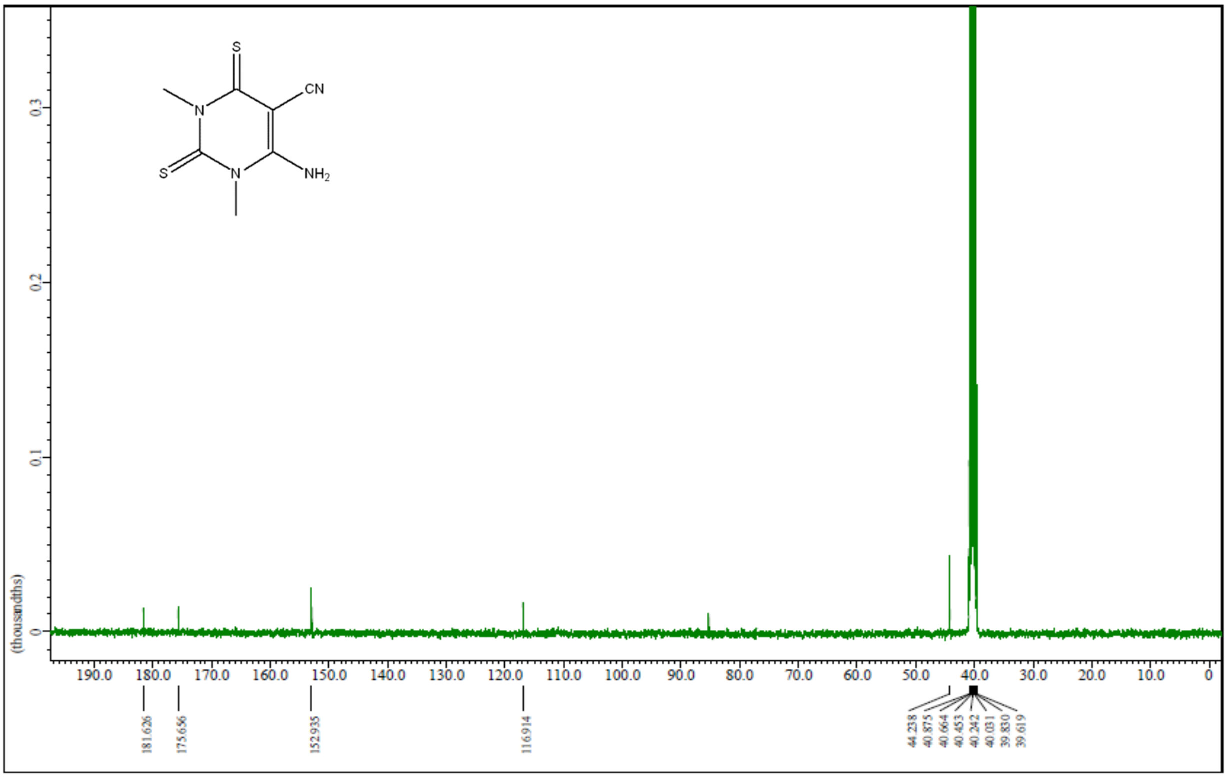

Figure S3. IR (KBr) spectrum of compound **7**

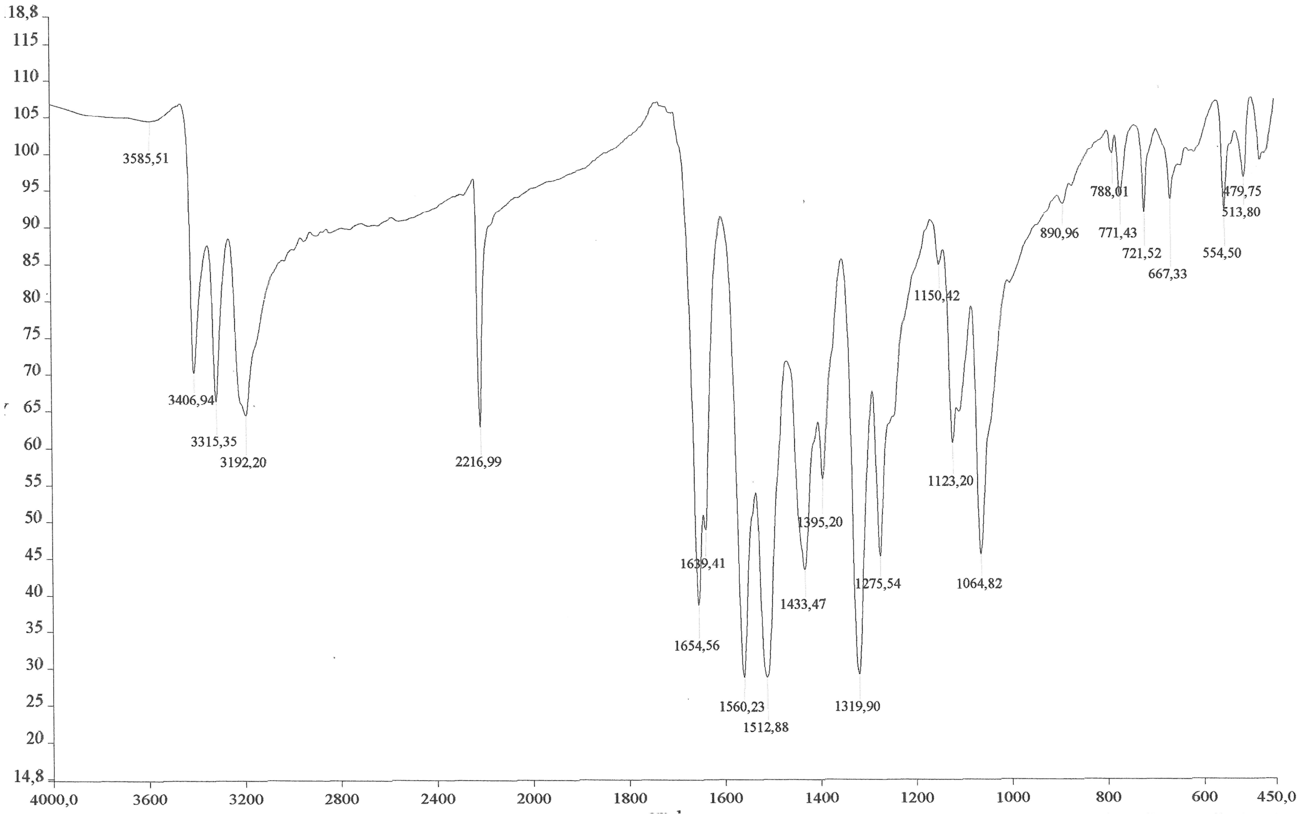

Figure S4. Mass spectrum of compound **7**

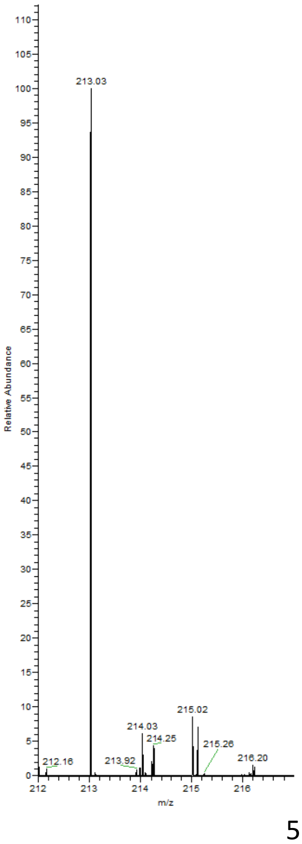

Figure S5. ^{1}H-NMR (200 MHz, d_6-DMSO) spectrum of compound **8**

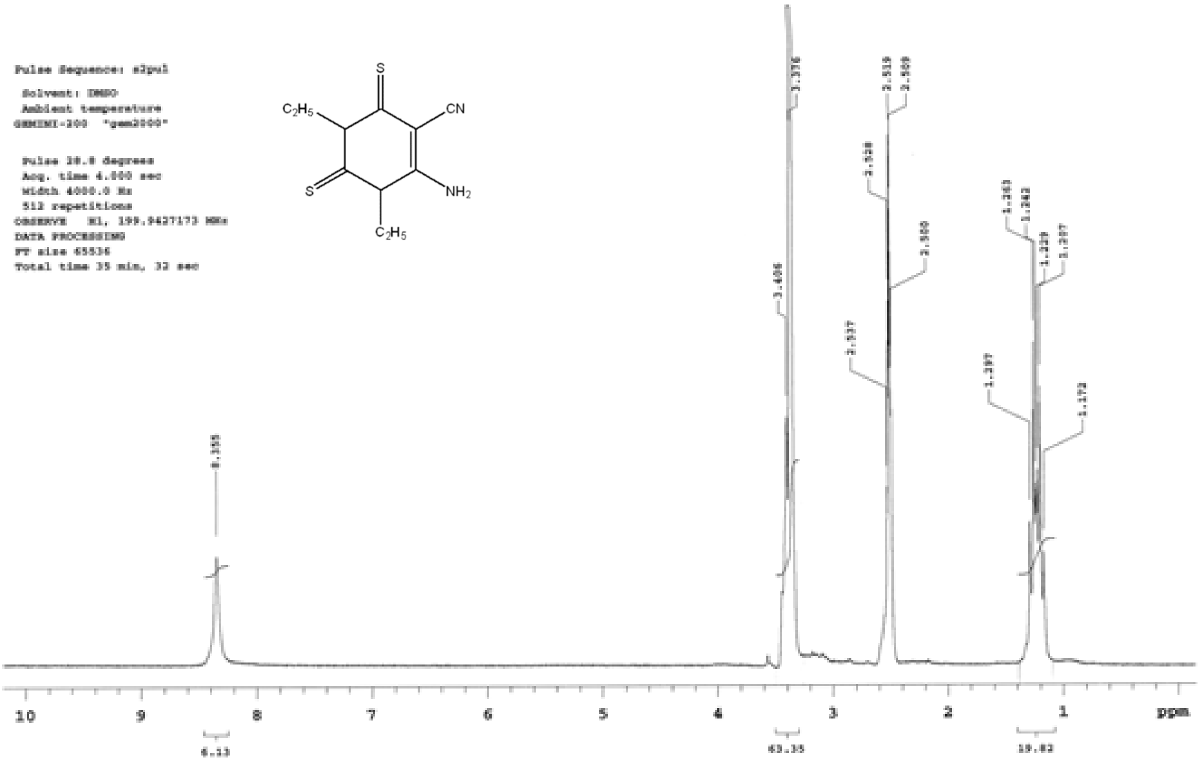

Figure S6. ^{13}C-NMR (101 MHz, d_6-DMSO) spectrum of compound **8**

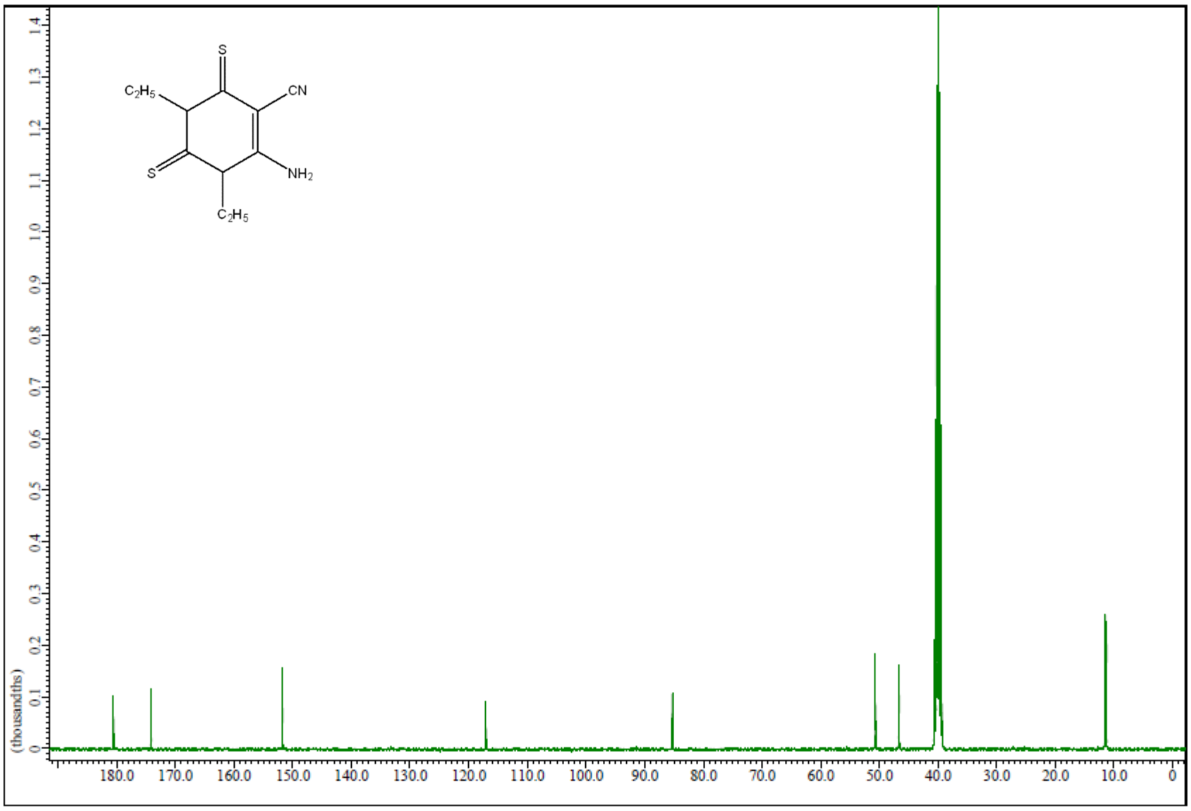

Figure S7. IR (KBr) spectrum of compound **8**

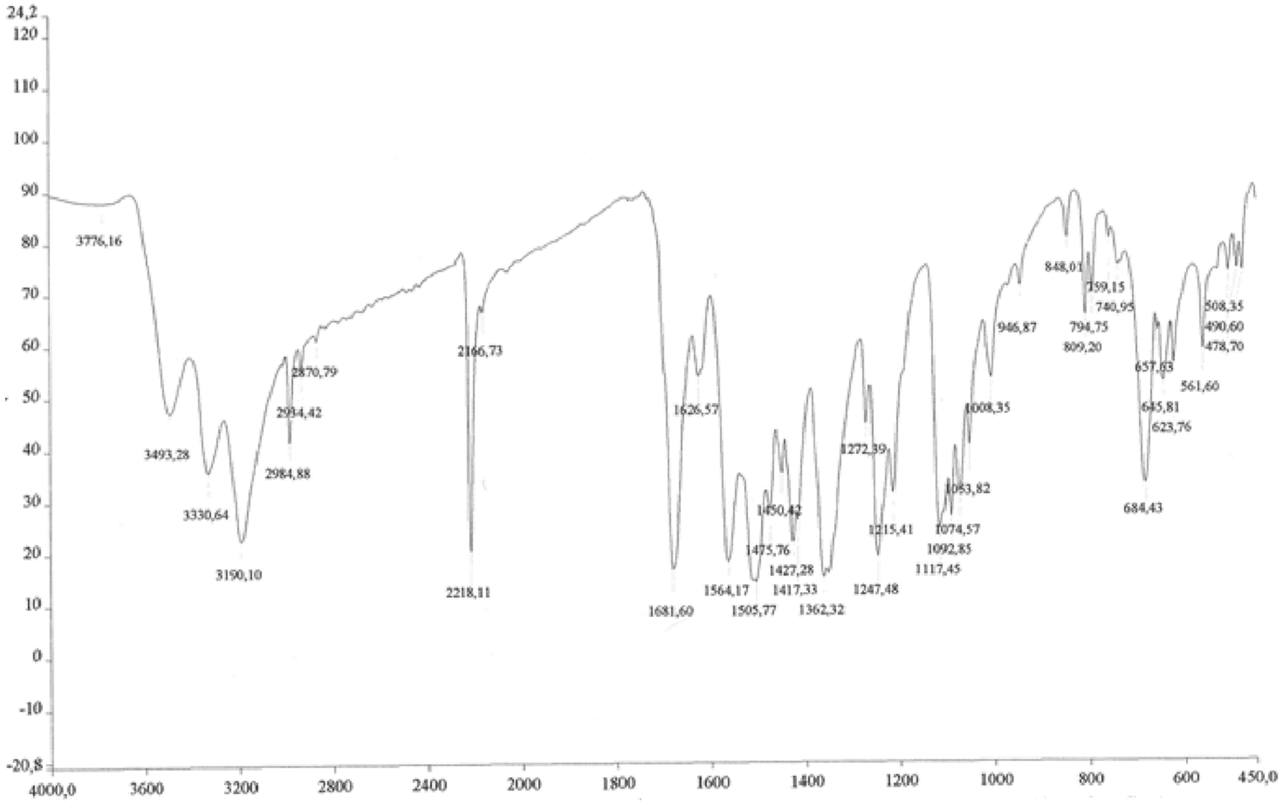

Figure S8. Mass spectrum of compound **8**

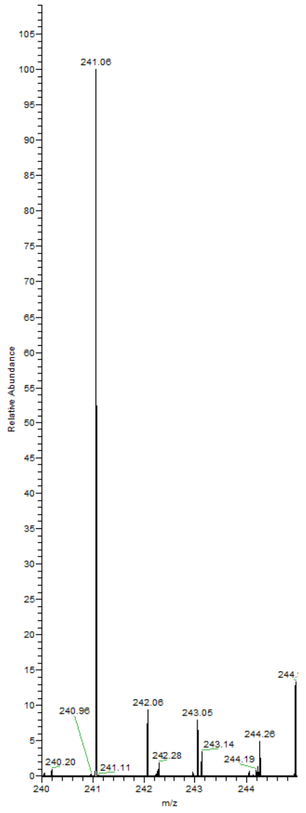

Figure S9. ^{1}H-NMR (200 MHz, $CDCl_3$) spectrum of compound **9**

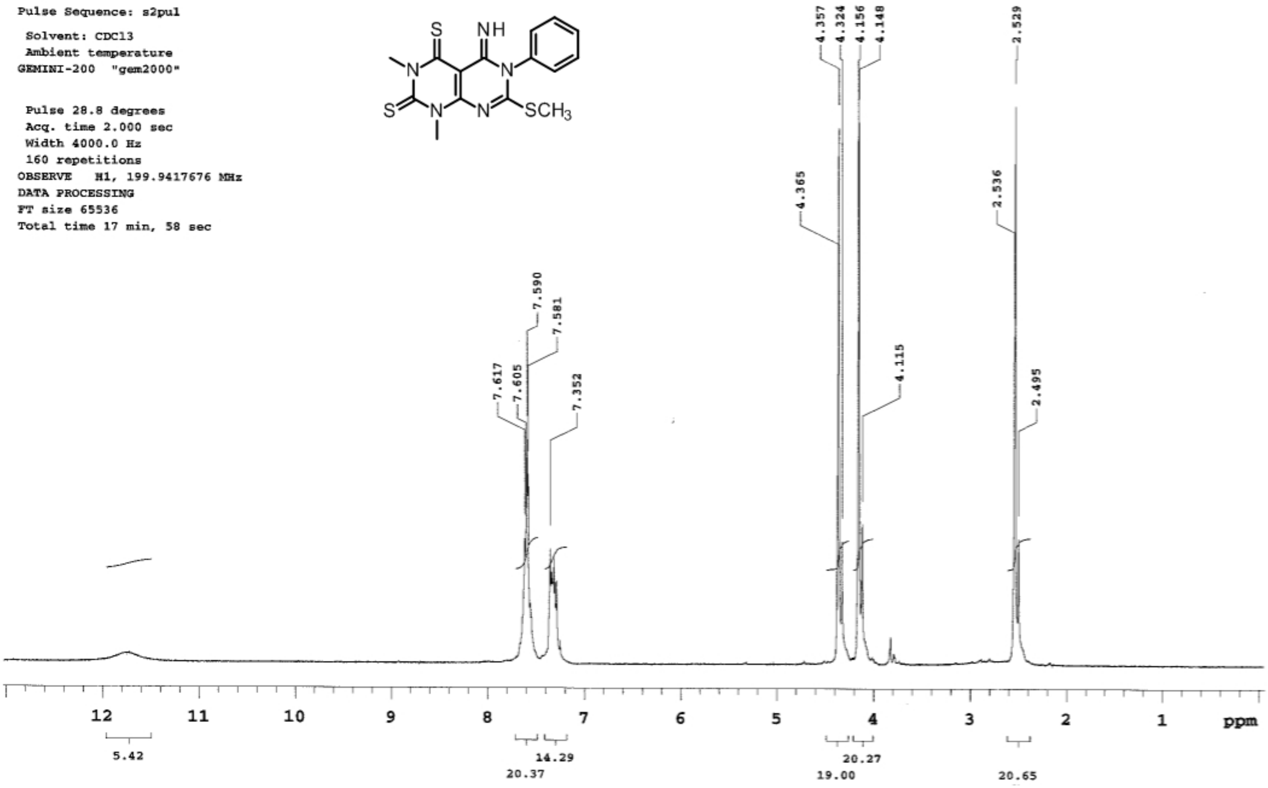

Figure S10. ^{13}C-NMR (101 MHz, $CDCl_3$) spectrum of compound **9**

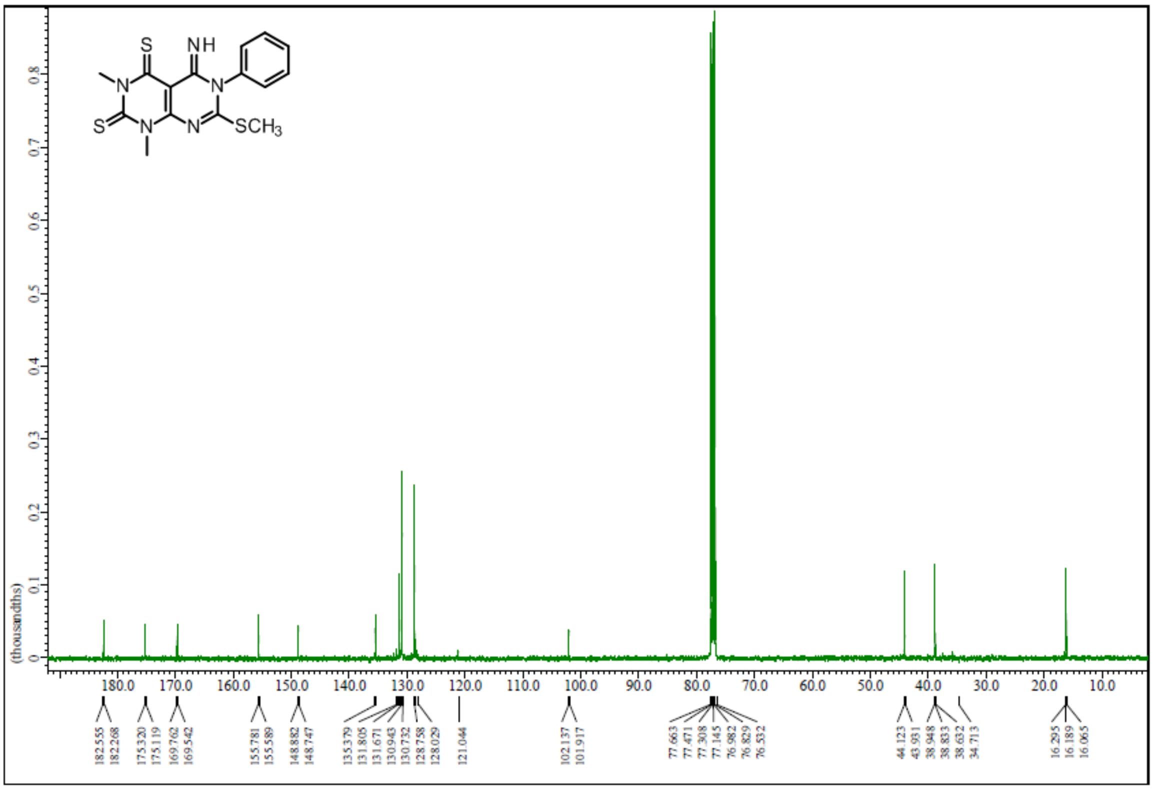

Figure S11. IR (KBr) spectrum of compound **9**

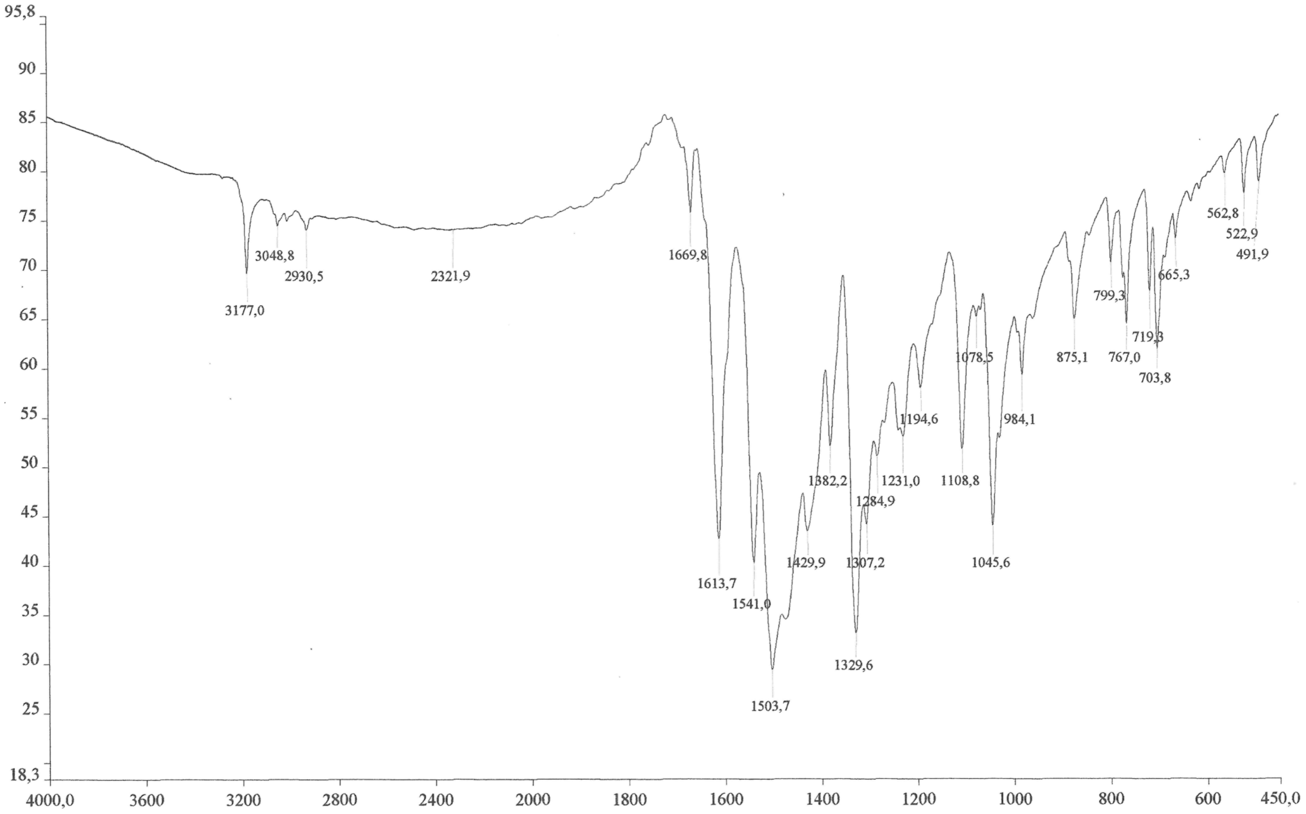

Figure S12. Mass spectrum of compound **9**

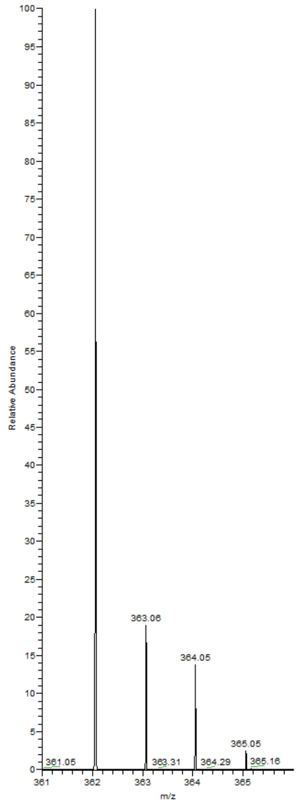

Figure S13. ^{1}H-NMR (200 MHz, $CDCl_3$) spectrum of compound **10**

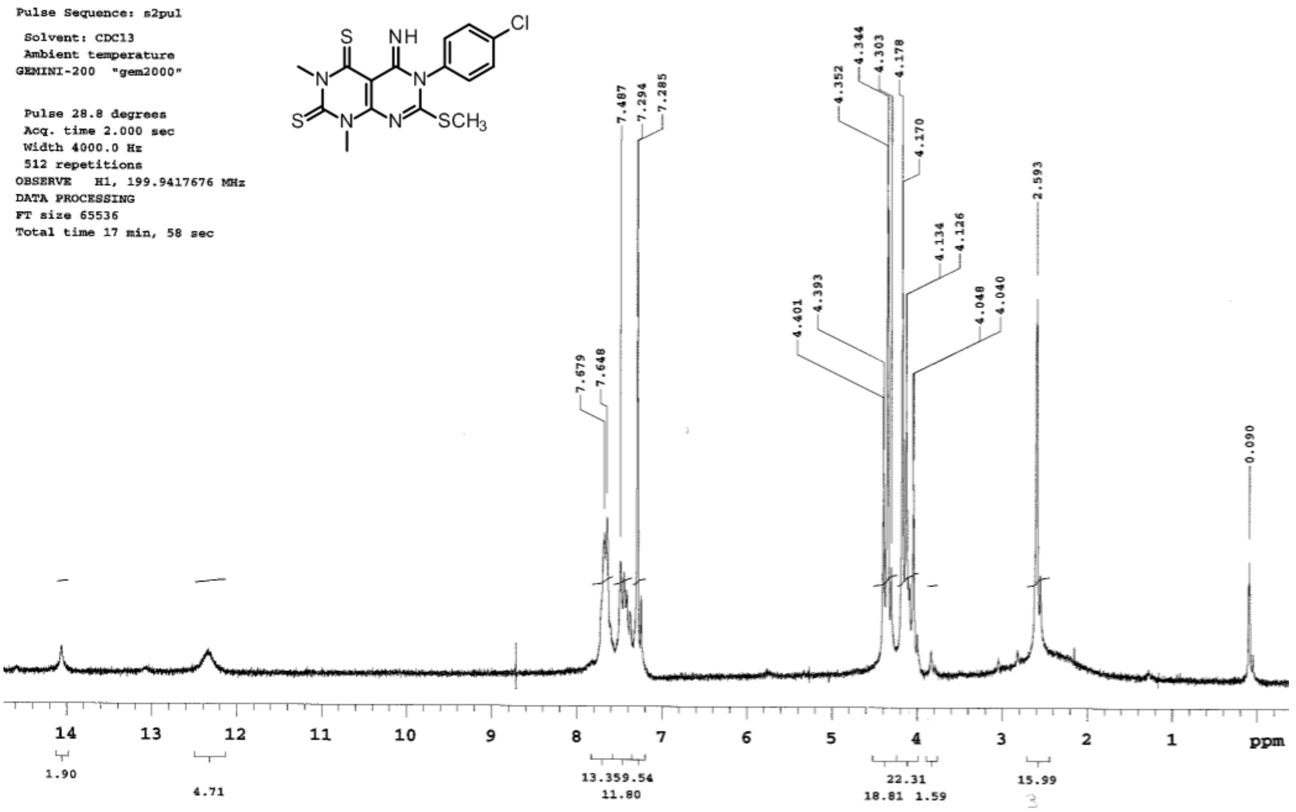

Figure S14. ^{13}C-NMR (101 MHz, $CDCl_3$) spectrum of compound **10**

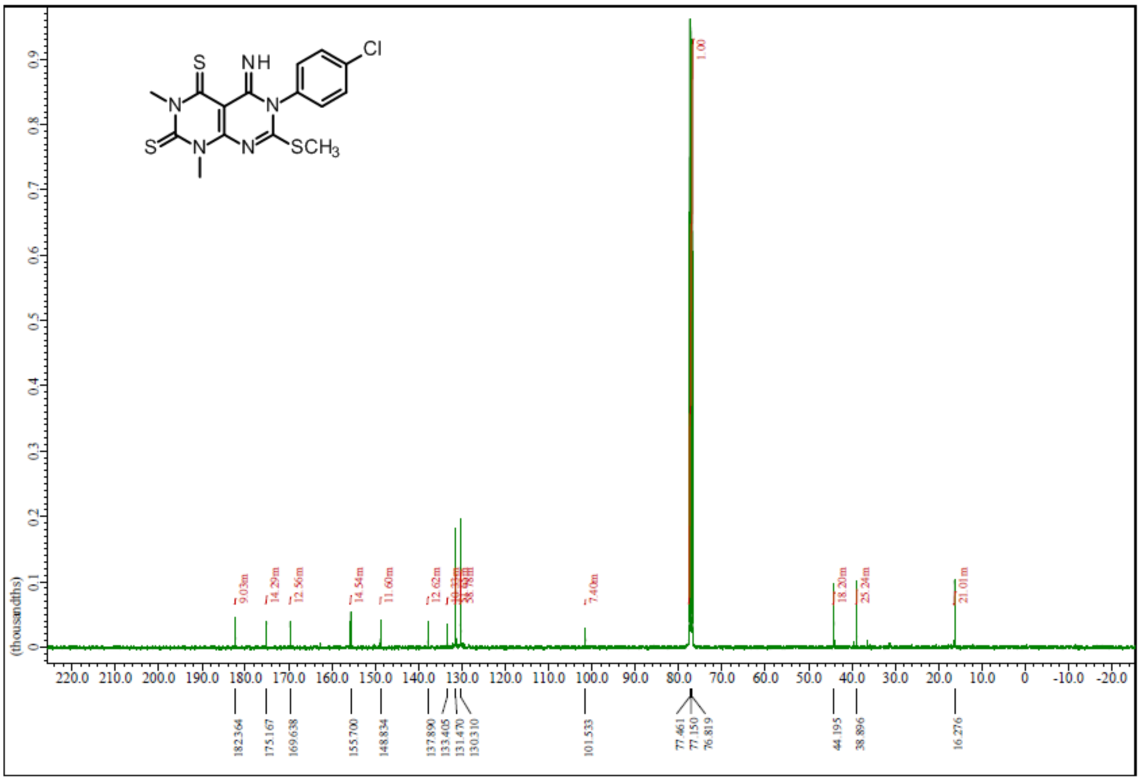

Figure S15. IR (KBr) spectrum of compound **10**

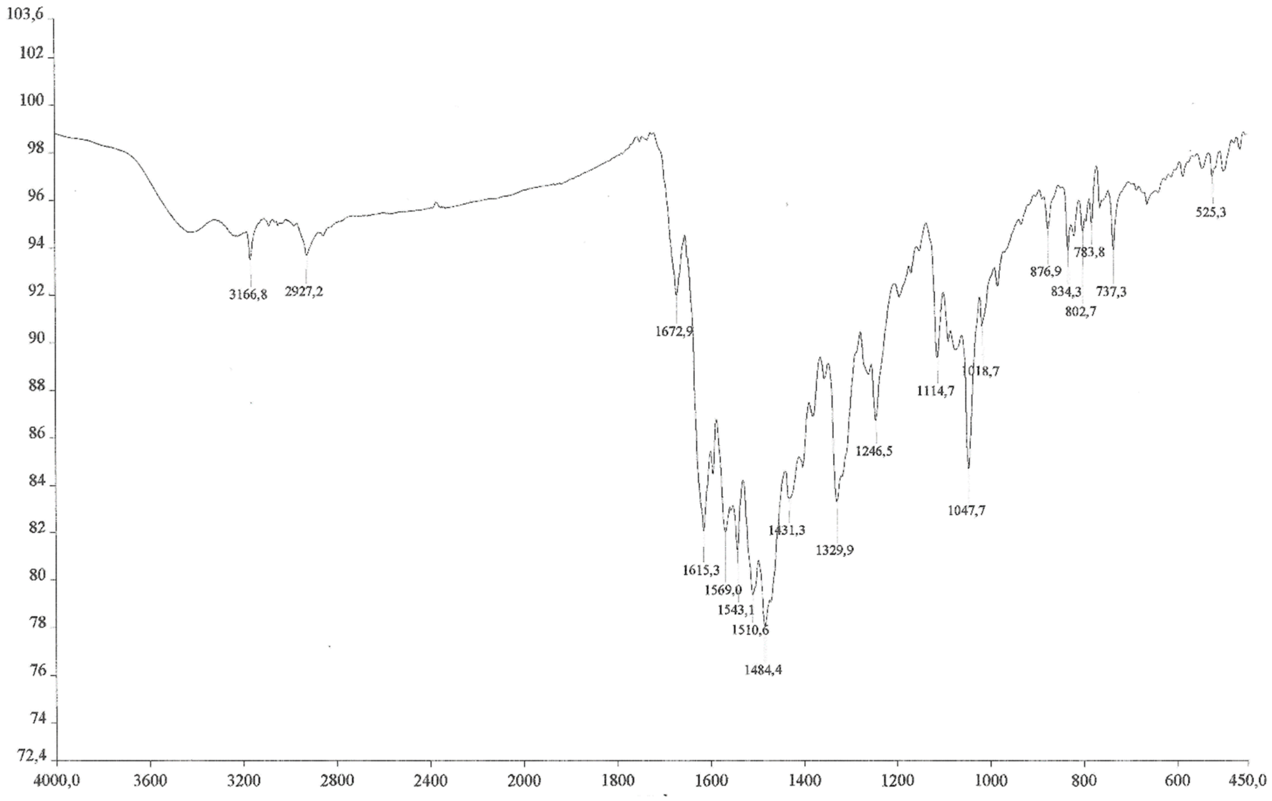

Figure S16. Mass spectrum of compound **10**

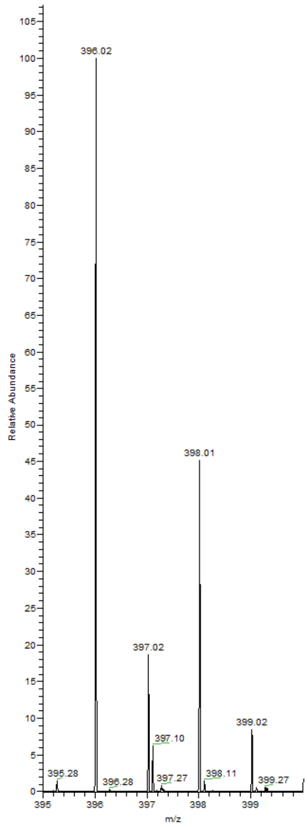

Figure S17. ^{1}H-NMR (300 MHz, d_6-DMSO) spectrum of compound **11**

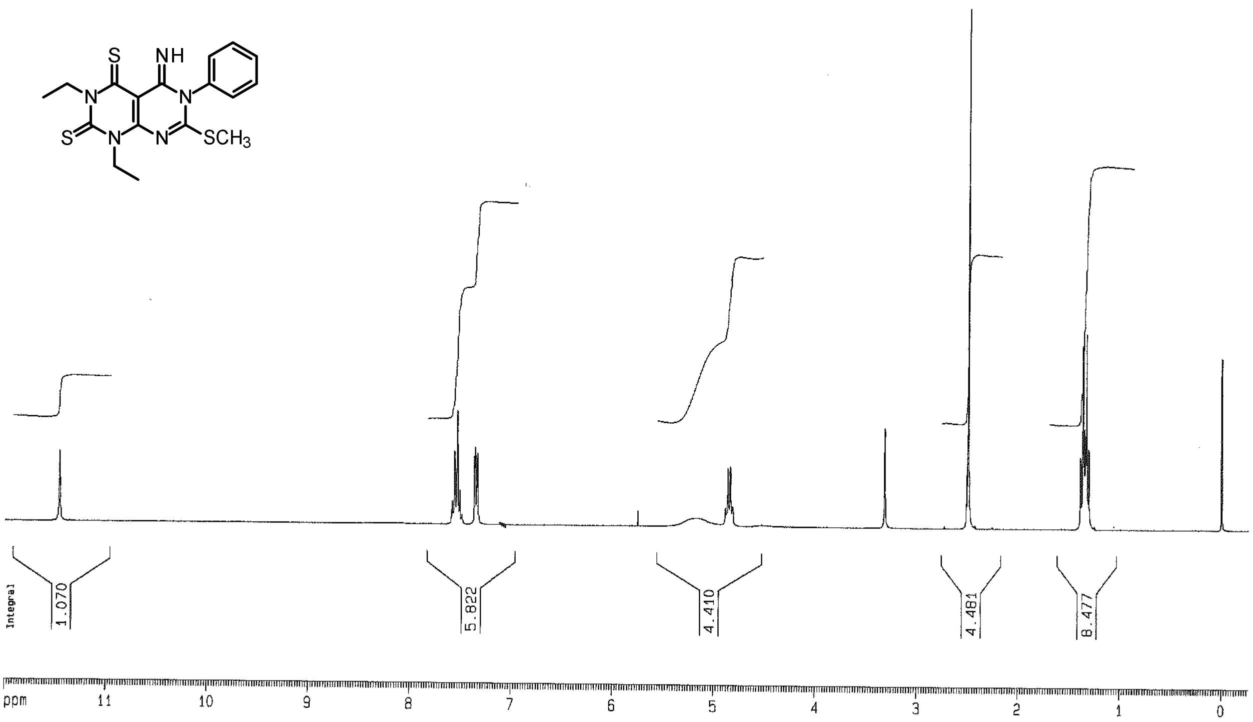

Figure S18. ^{13}C-NMR (75 MHz, d_6-DMSO) spectrum of compound **11**

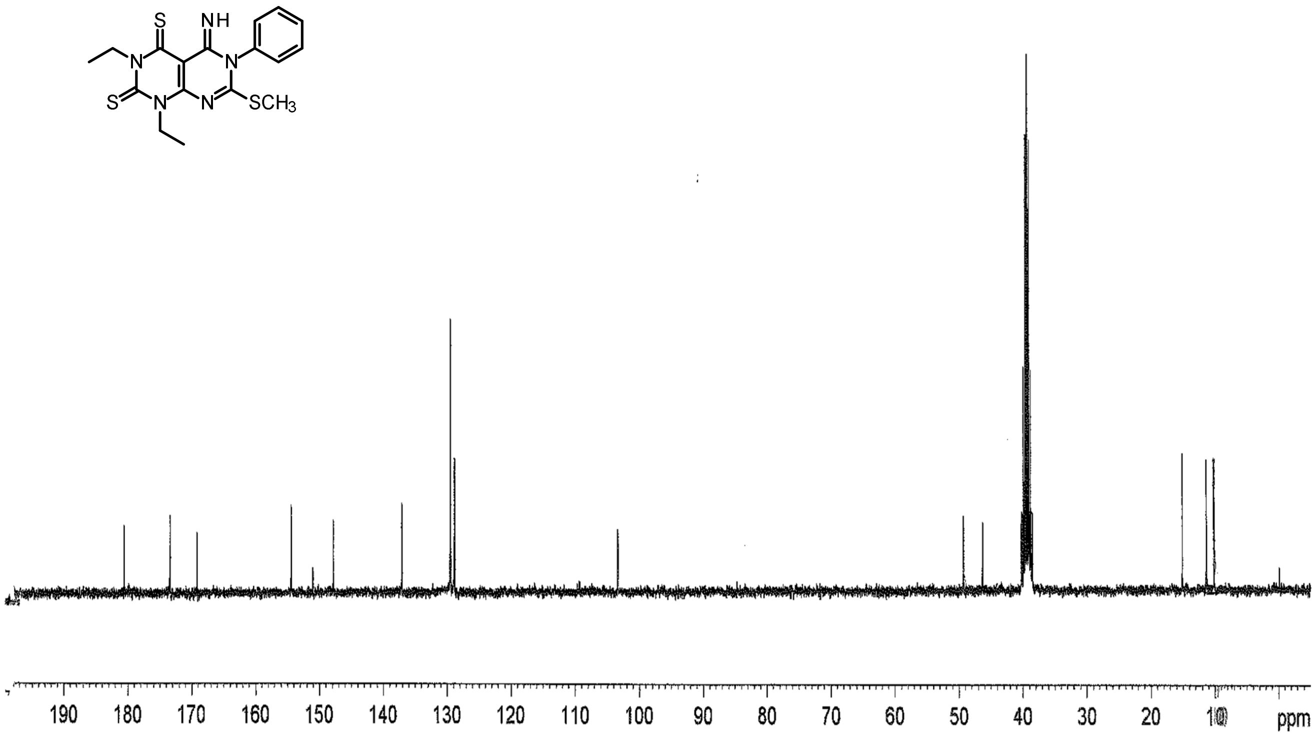

Figure S19. IR (KBr) spectrum of compound **11**

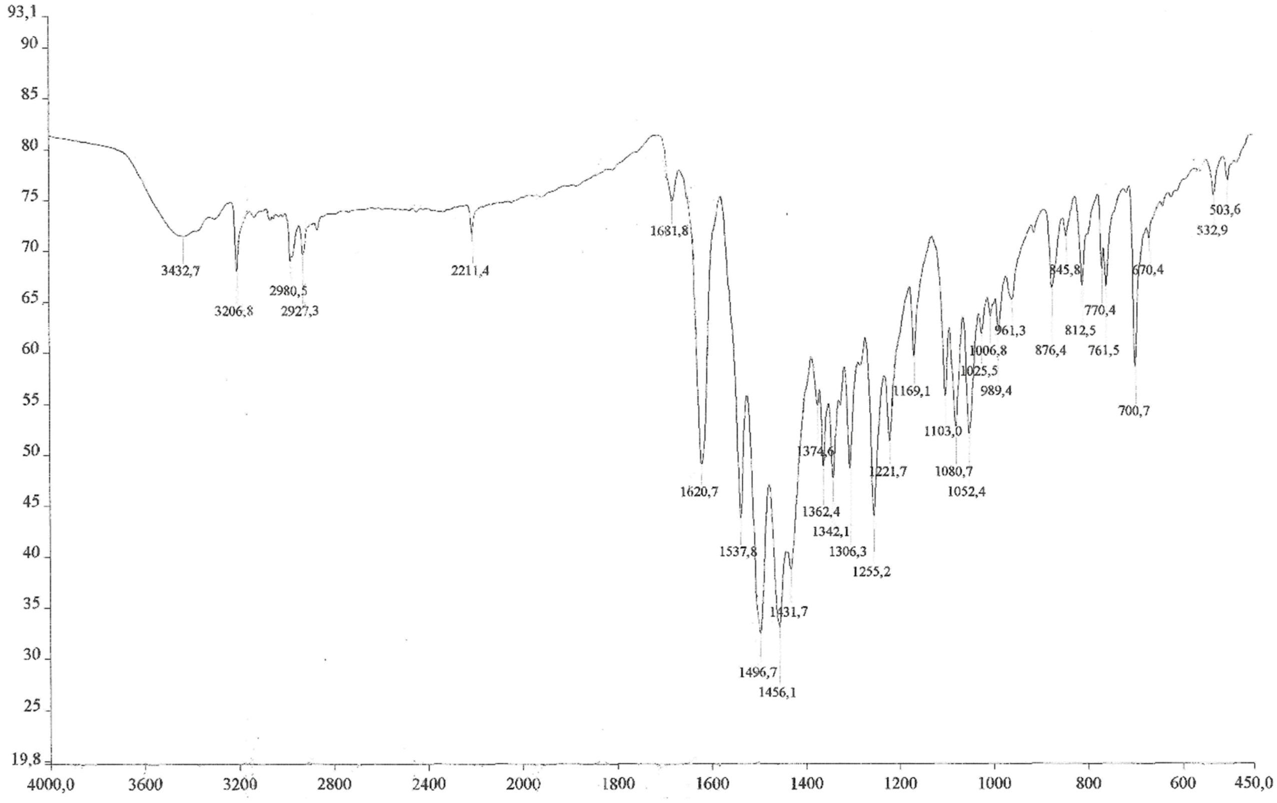

Figure S20. Mass spectrum of compound **11**

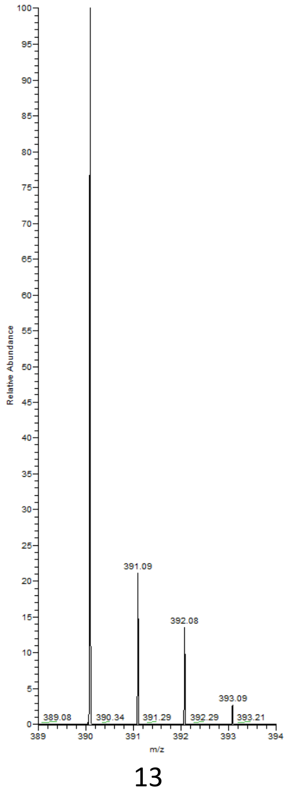

Figure S21. ^{1}H-NMR (200 MHz, $CDCl_3$) spectrum of compound **12**

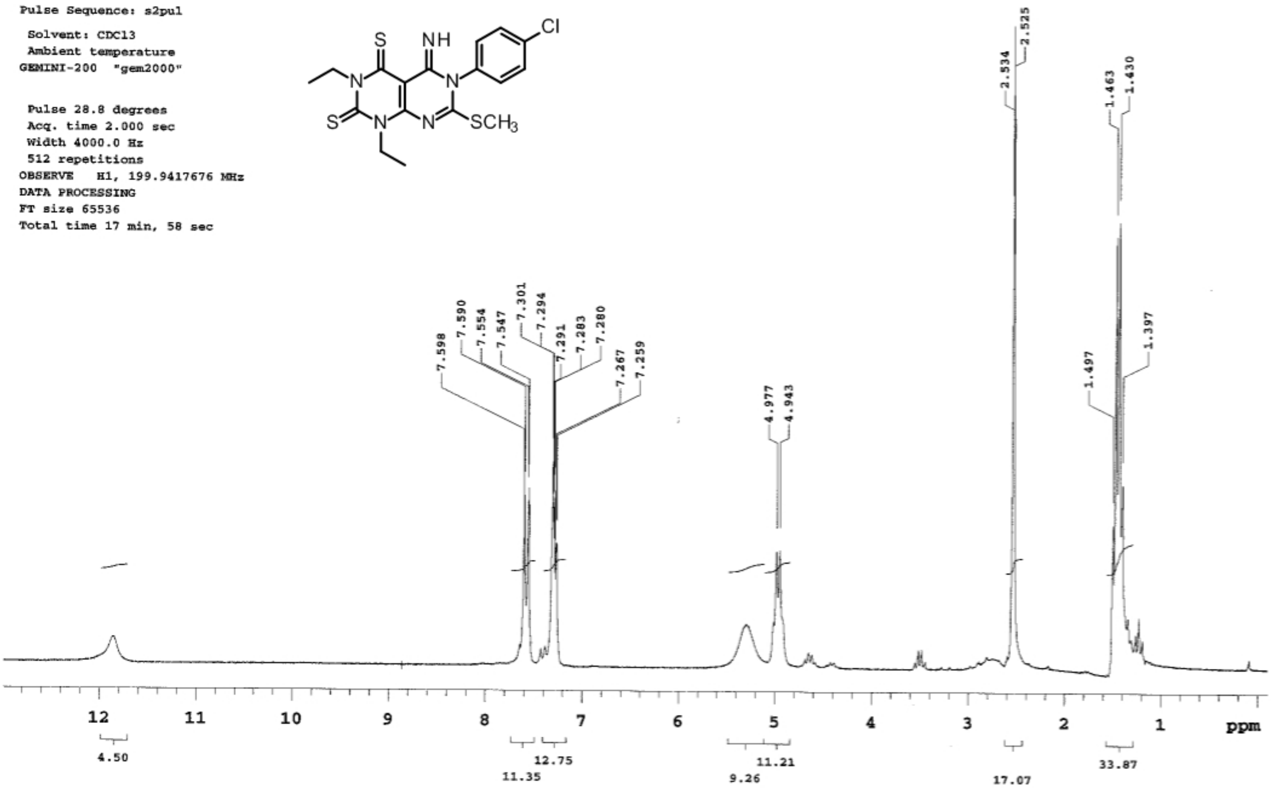

Figure S22. ^{13}C-NMR (101 MHz, $CDCl_3$) spectrum of compound **12**

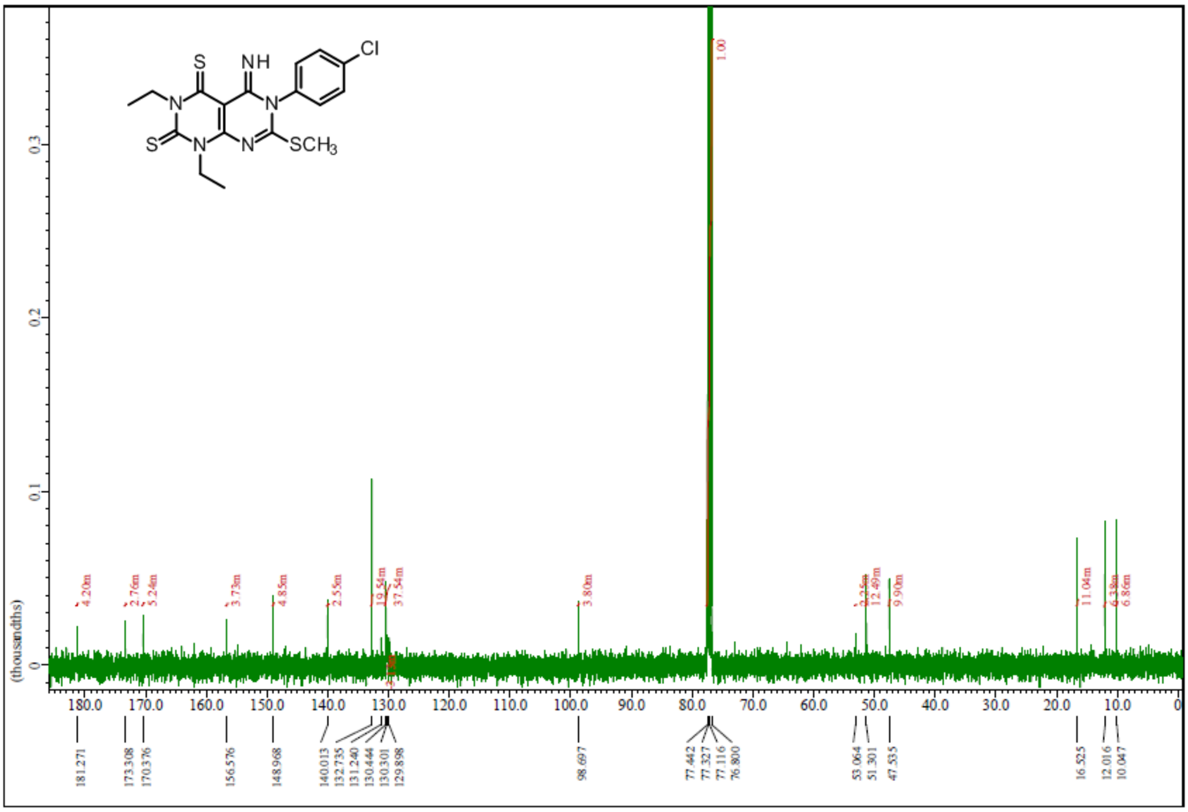

Figure S23. IR (KBr) spectrum of compound **12**

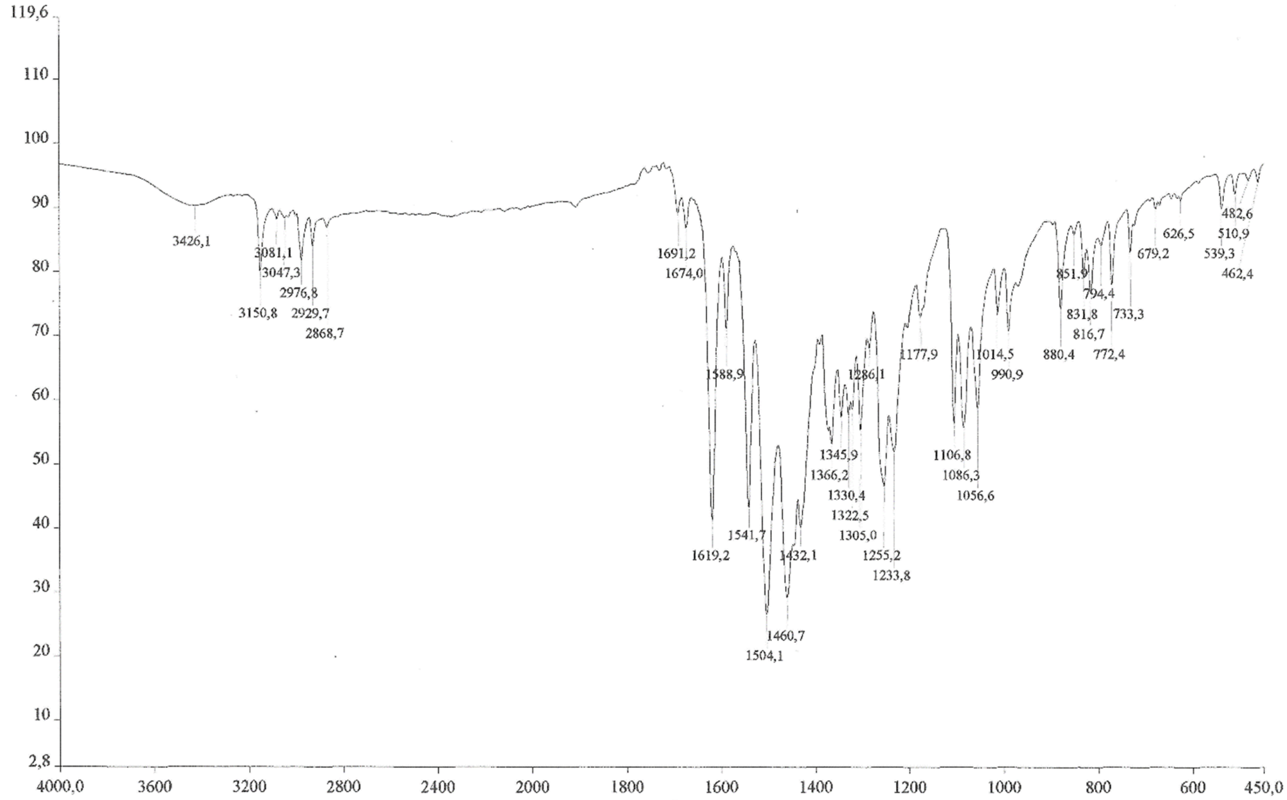

Figure S24. Mass spectrum of compound **12**

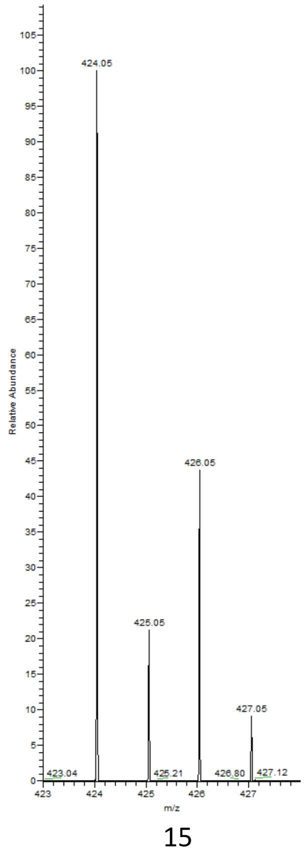

Figure S25. ^{1}H-NMR (200 MHz, $CDCl_3$) spectrum of compound **3a**

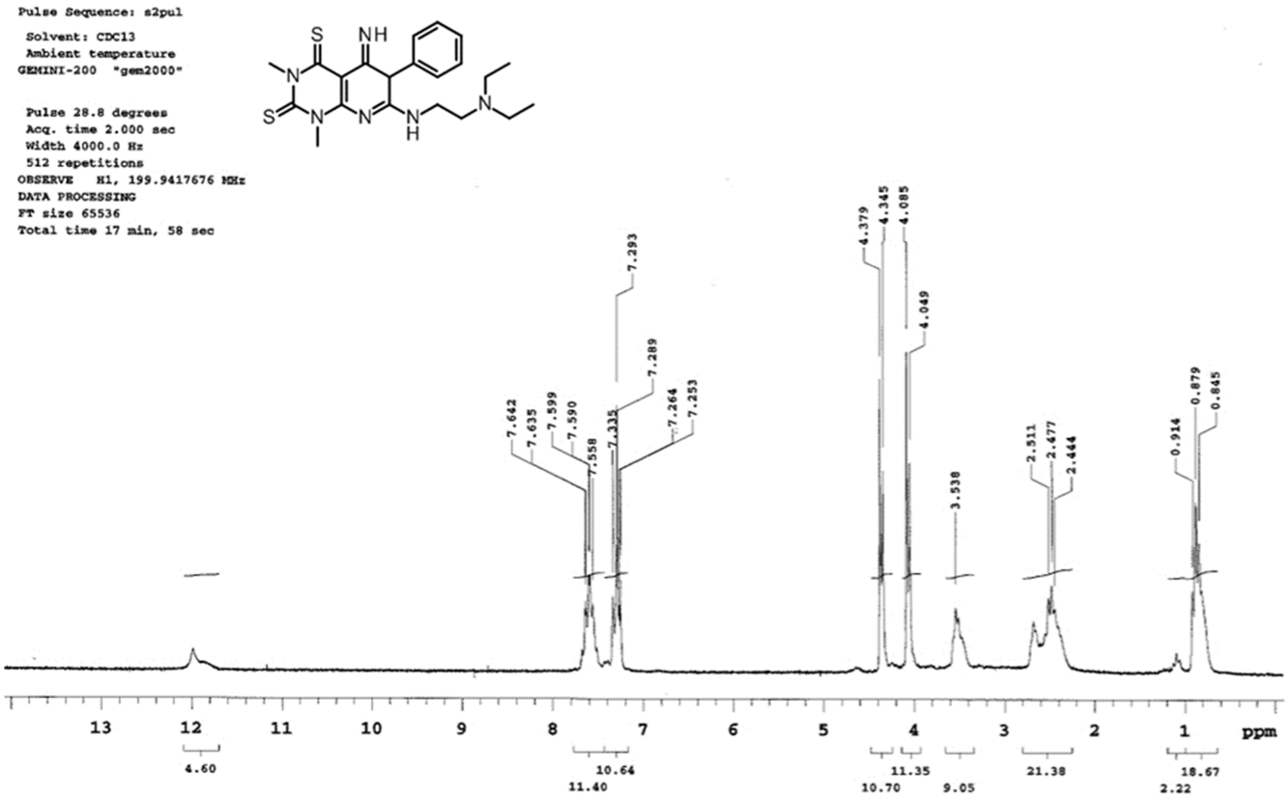

Figure S26. ^{13}C-NMR (101 MHz, $CDCl_3$) spectrum of compound **3a**

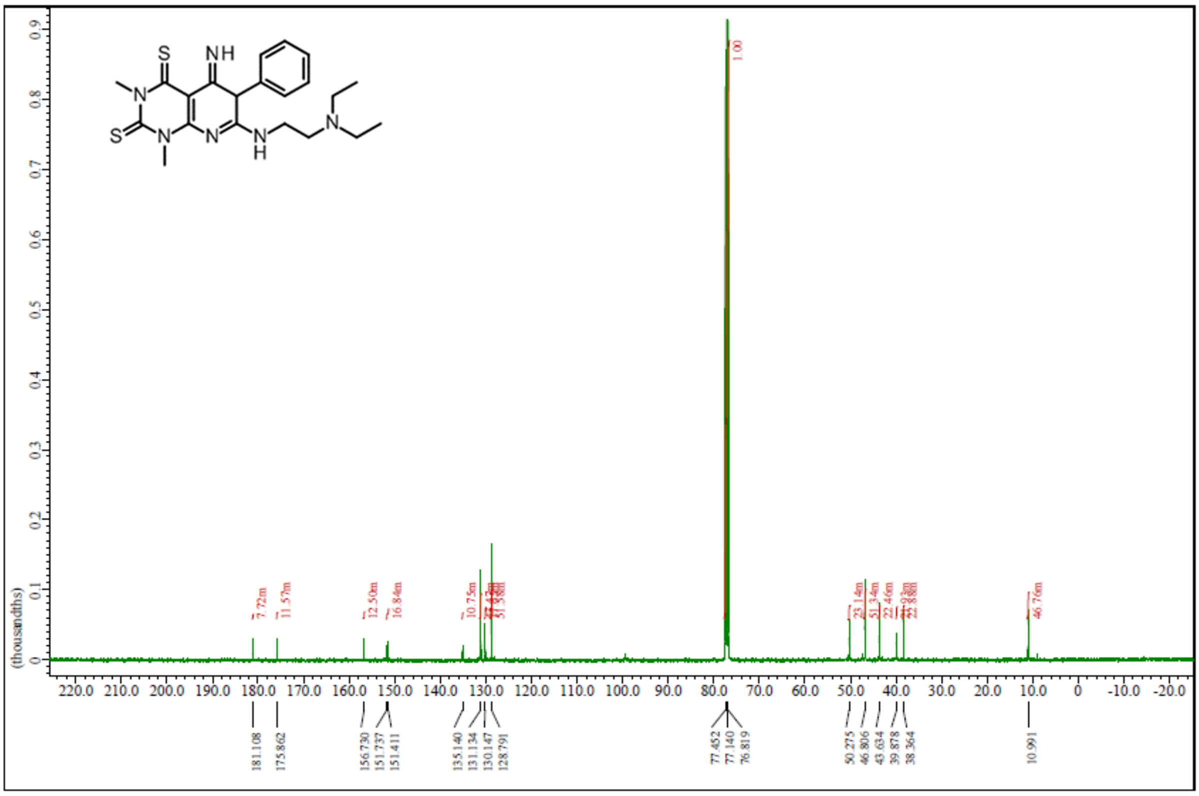

Figure S27. IR (KBr) spectrum of compound **3a**

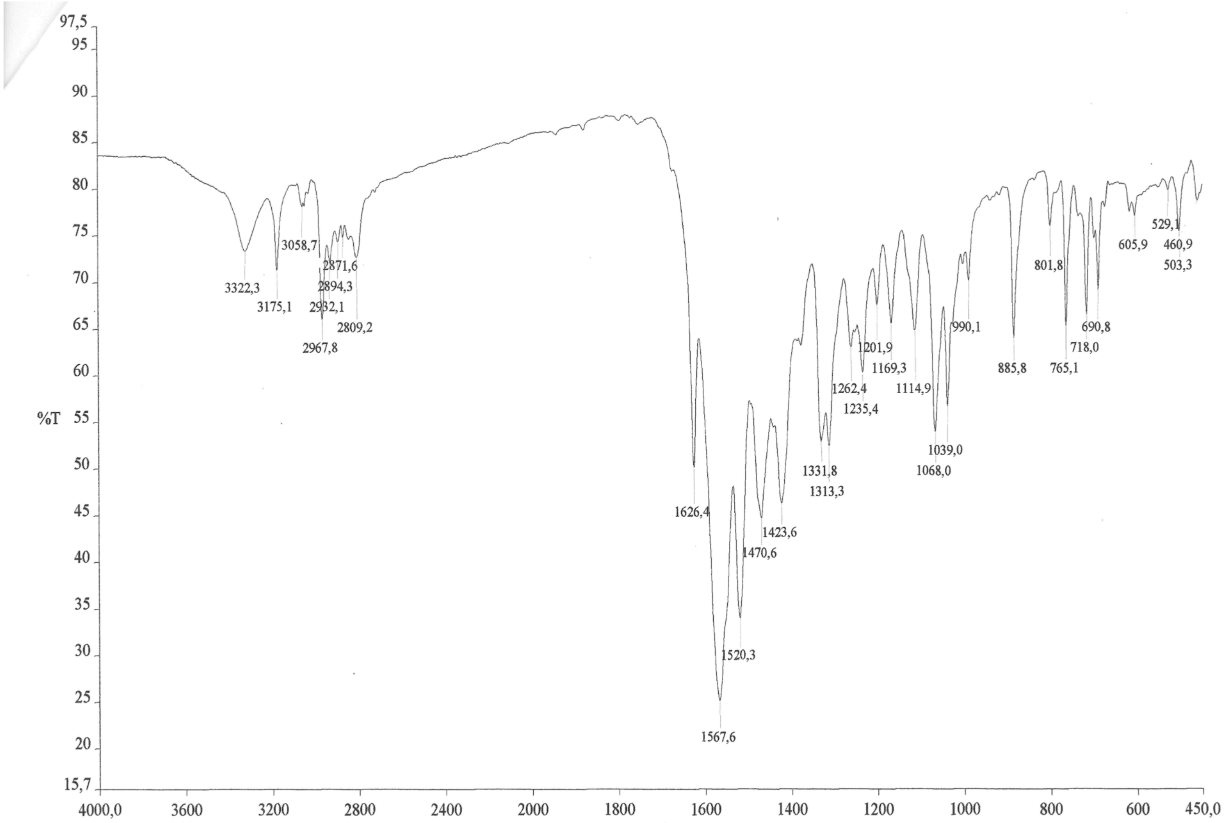

Figure S28. Mass spectrum of compound **3a**

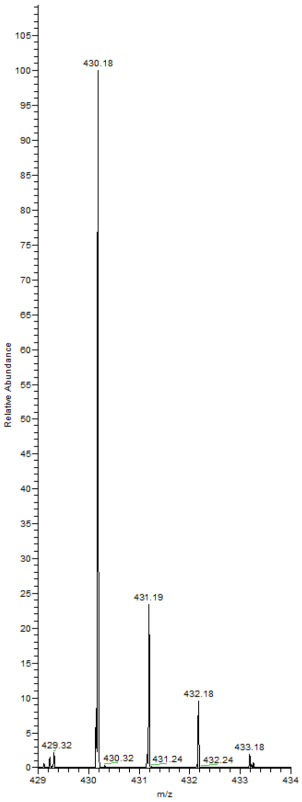

Figure S29. ^{1}H-NMR (200 MHz, $CDCl_3$) spectrum of compound **3b**

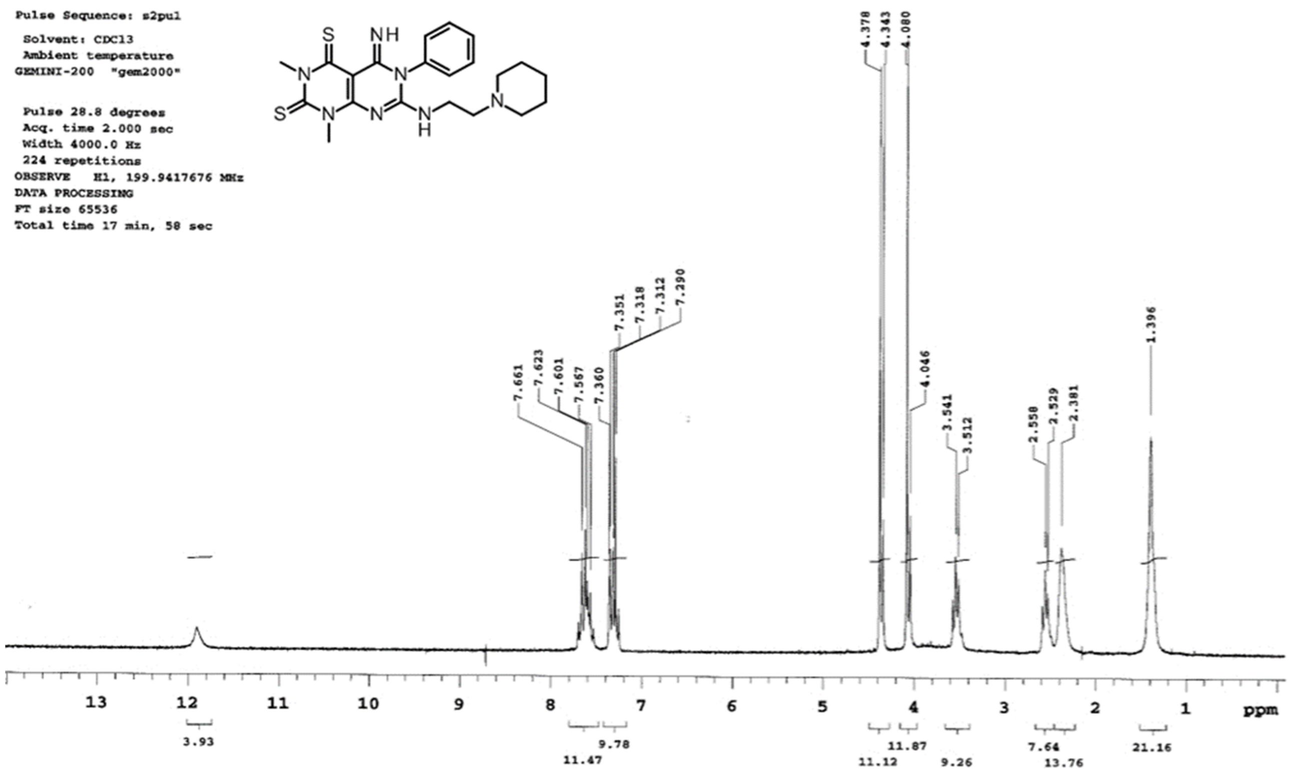

Figure S30. ^{13}C-NMR (101 MHz, $CDCl_3$) spectrum of compound **3b**

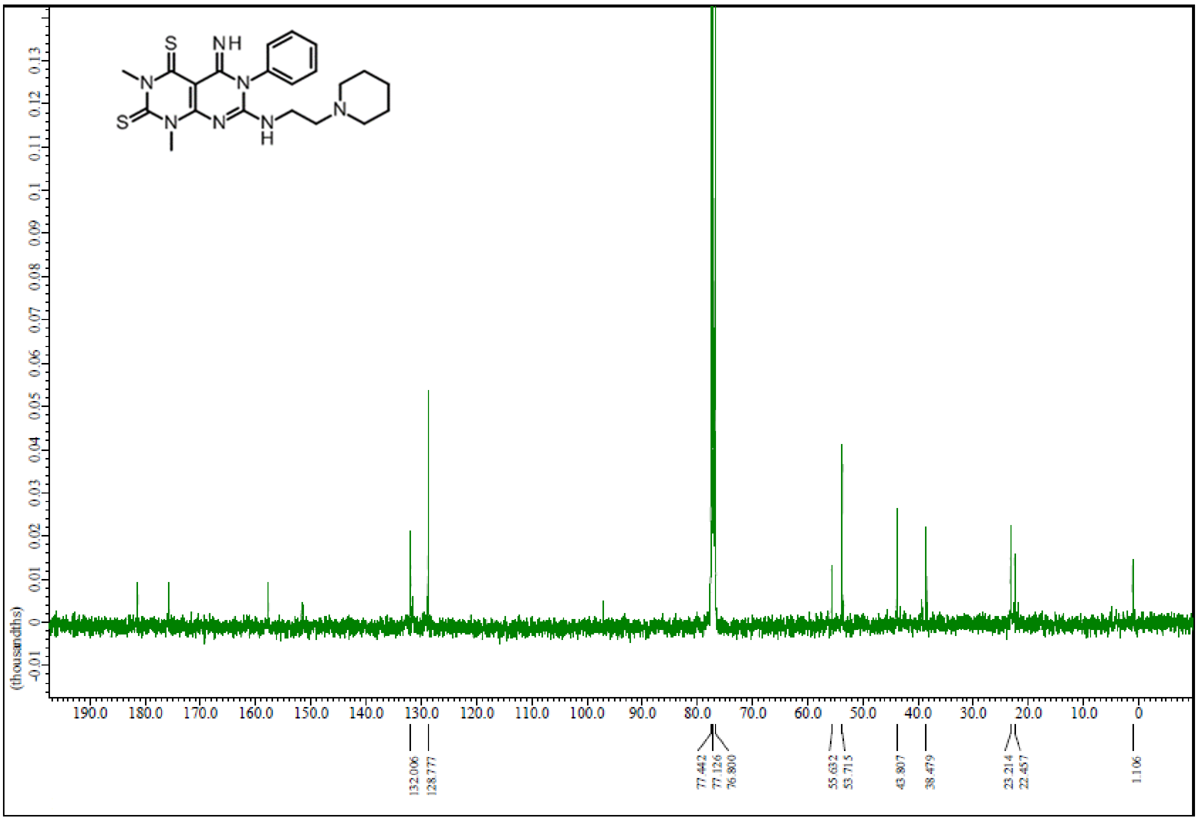

Figure S31. IR (KBr) spectrum of compound **3b**

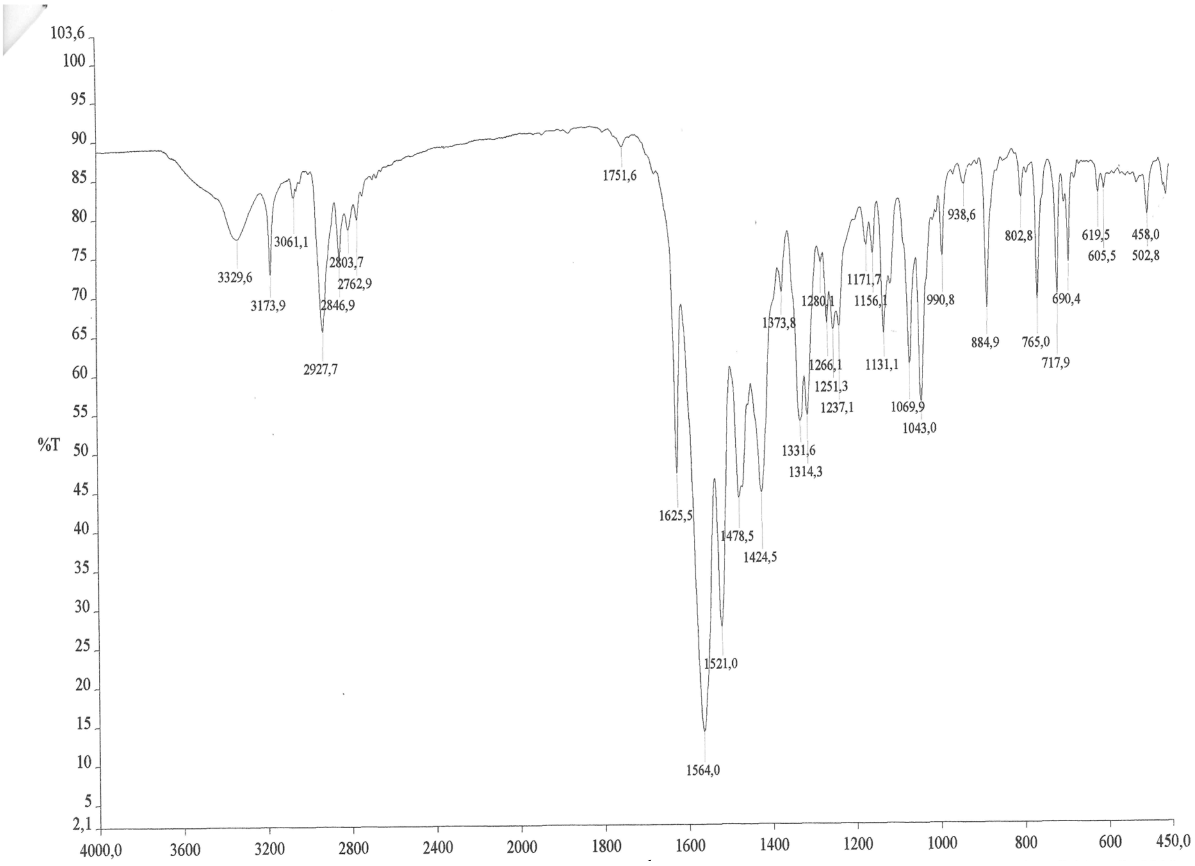

Figure S32. ^{1}H-NMR (200 MHz, $CDCl_3$) spectrum of compound **3c**

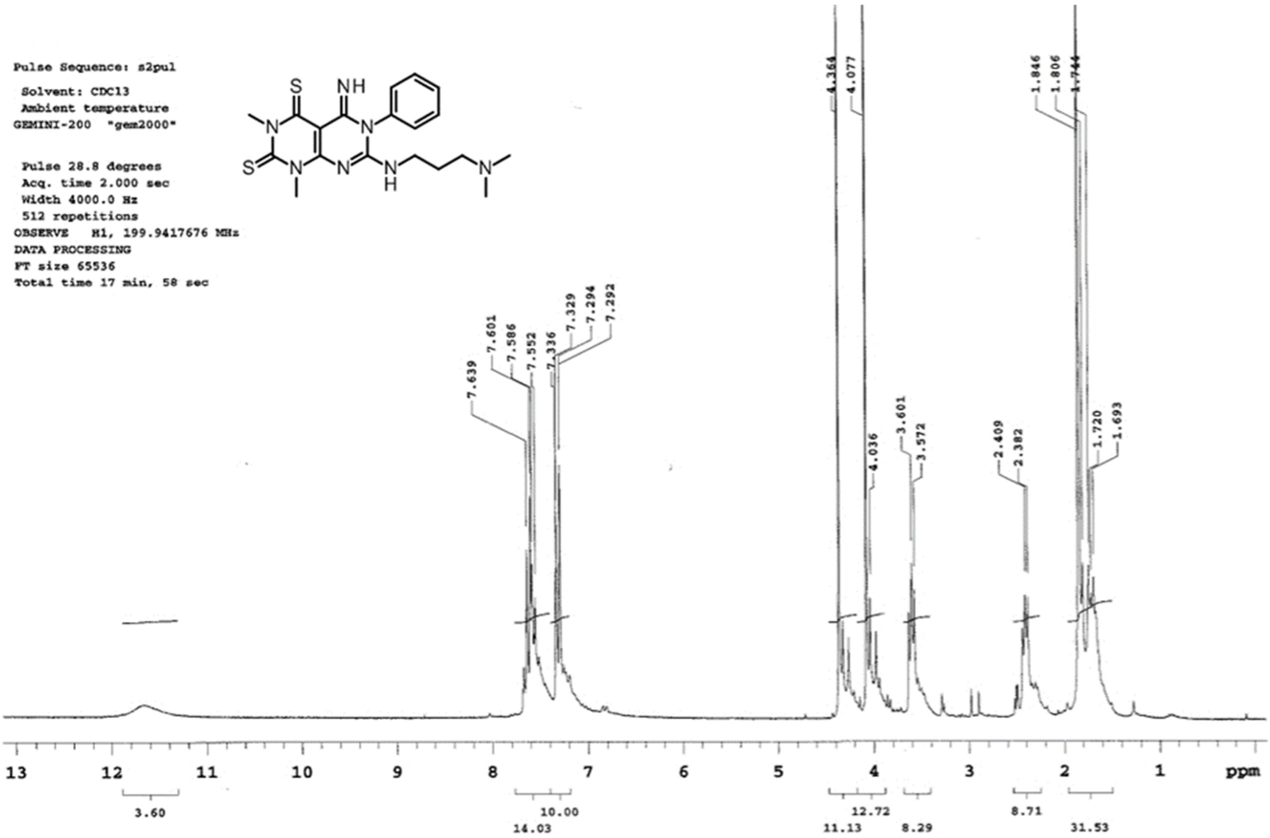

Figure S33. ^{13}C-NMR (101 MHz, $CDCl_3$) spectrum of compound **3c**

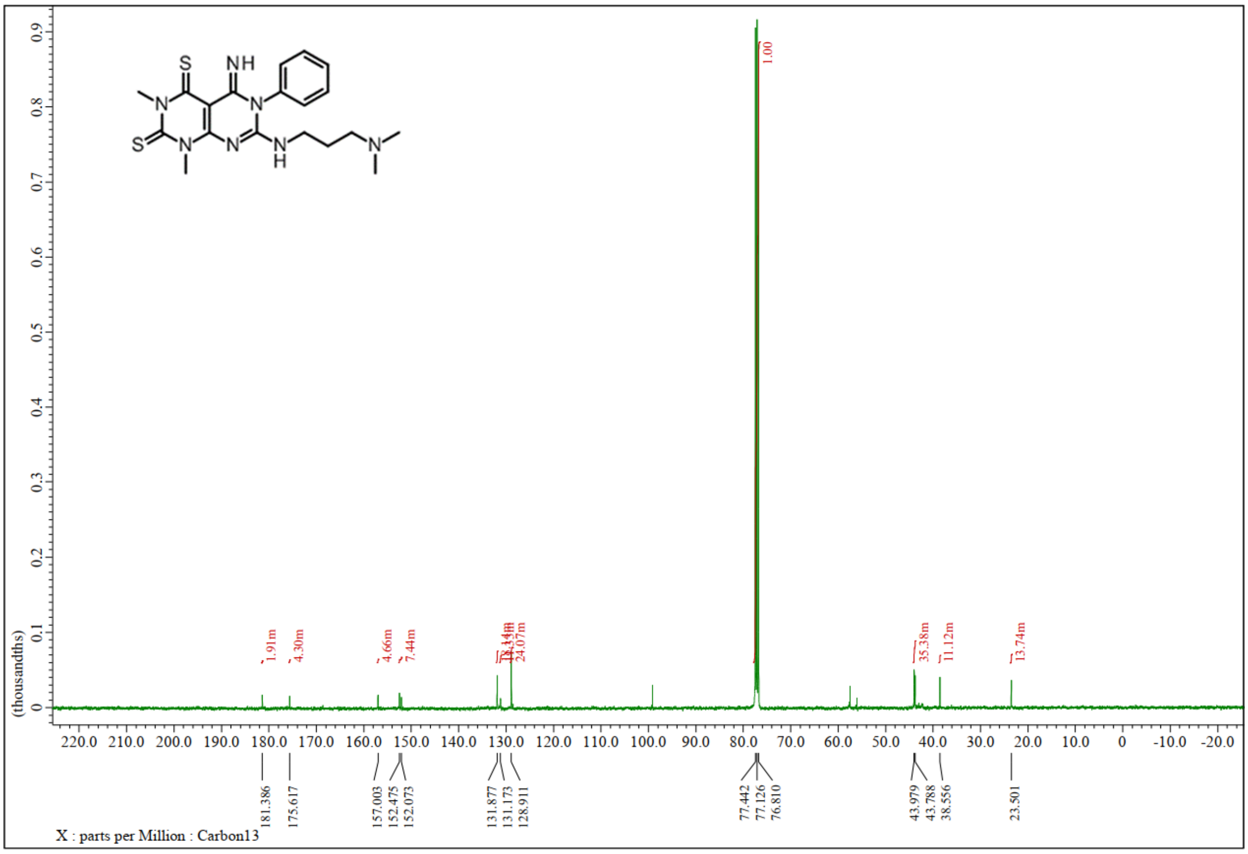

Figure S34. IR (KBr) spectrum of compound **3c**

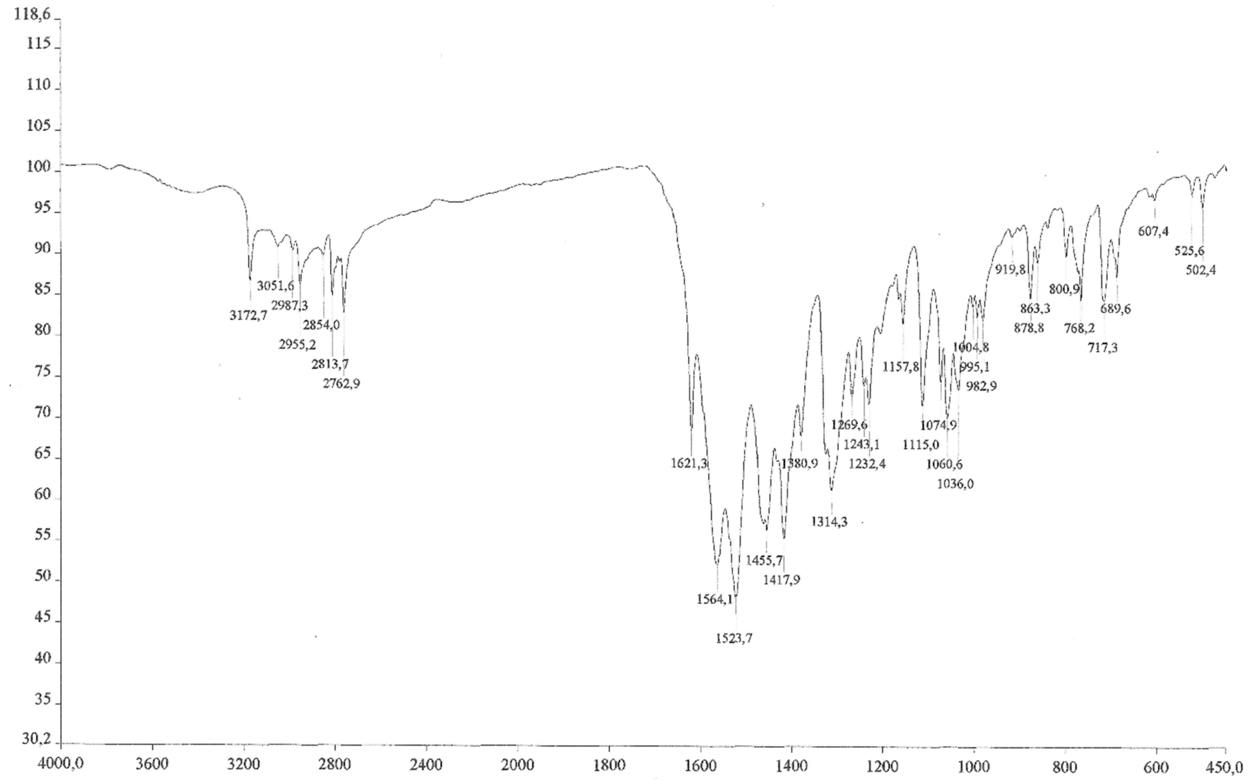

Figure S35. ^{1}H-NMR (200 MHz, $CDCl_3$) spectrum of compound **3d**

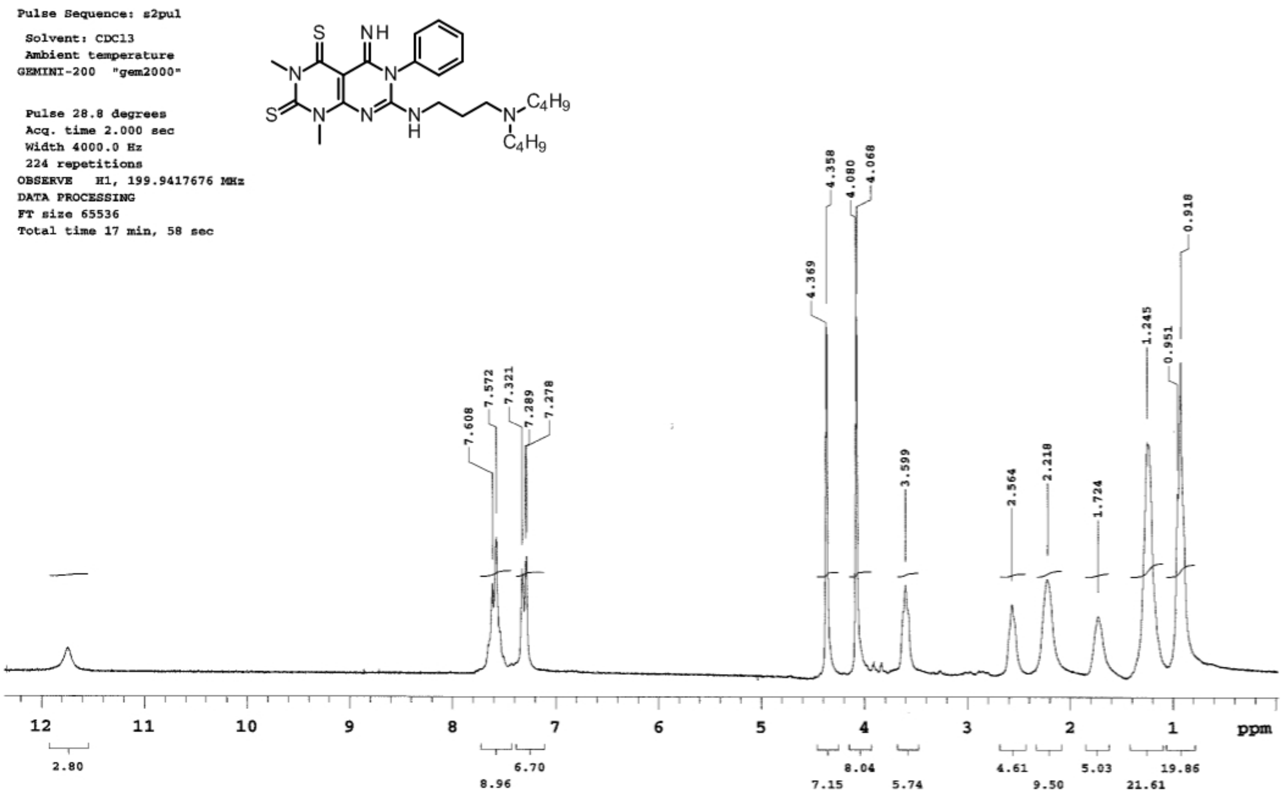

Figure S36. ^{13}C-NMR (101 MHz, $CDCl_3$) spectrum of compound **3d**

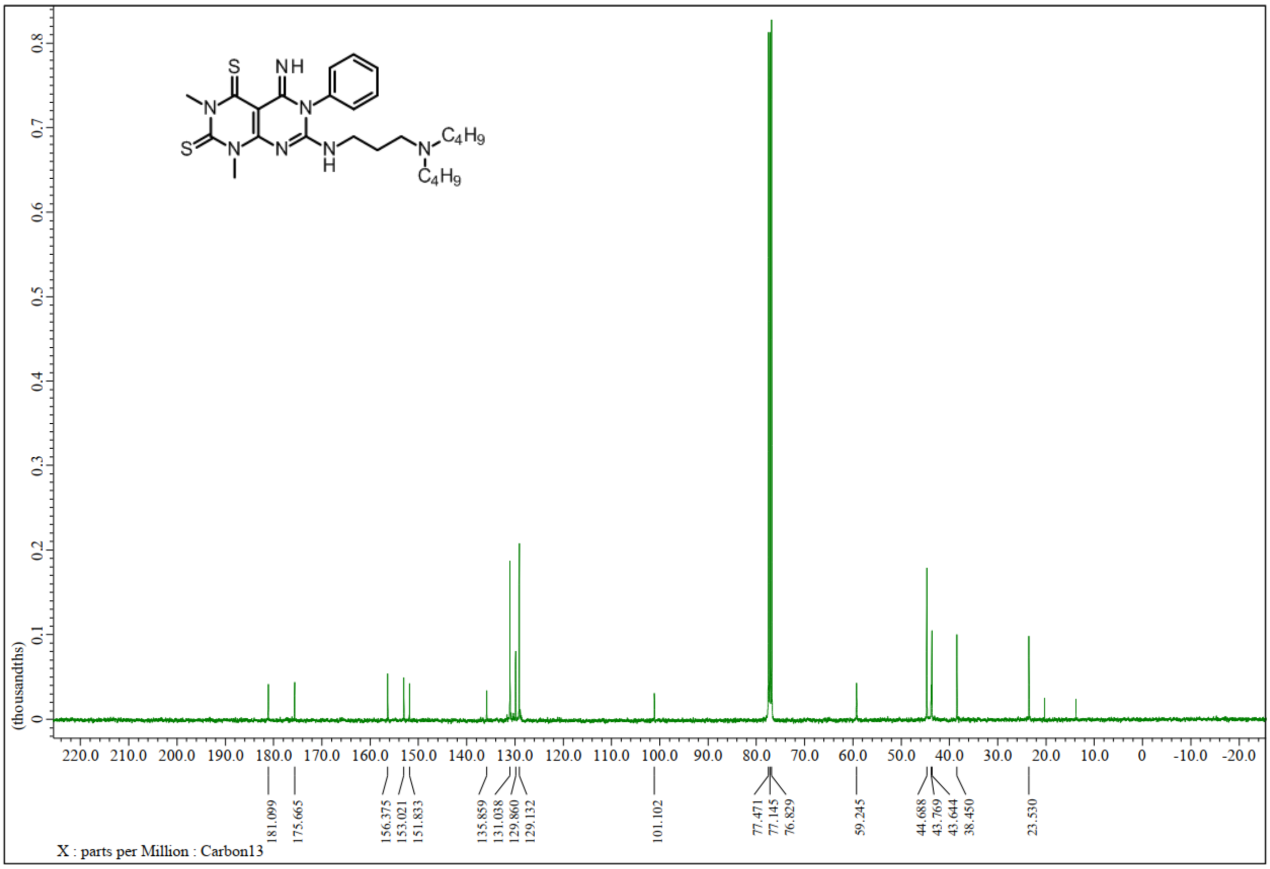

Figure S37. IR (KBr) spectrum of compound **3d**

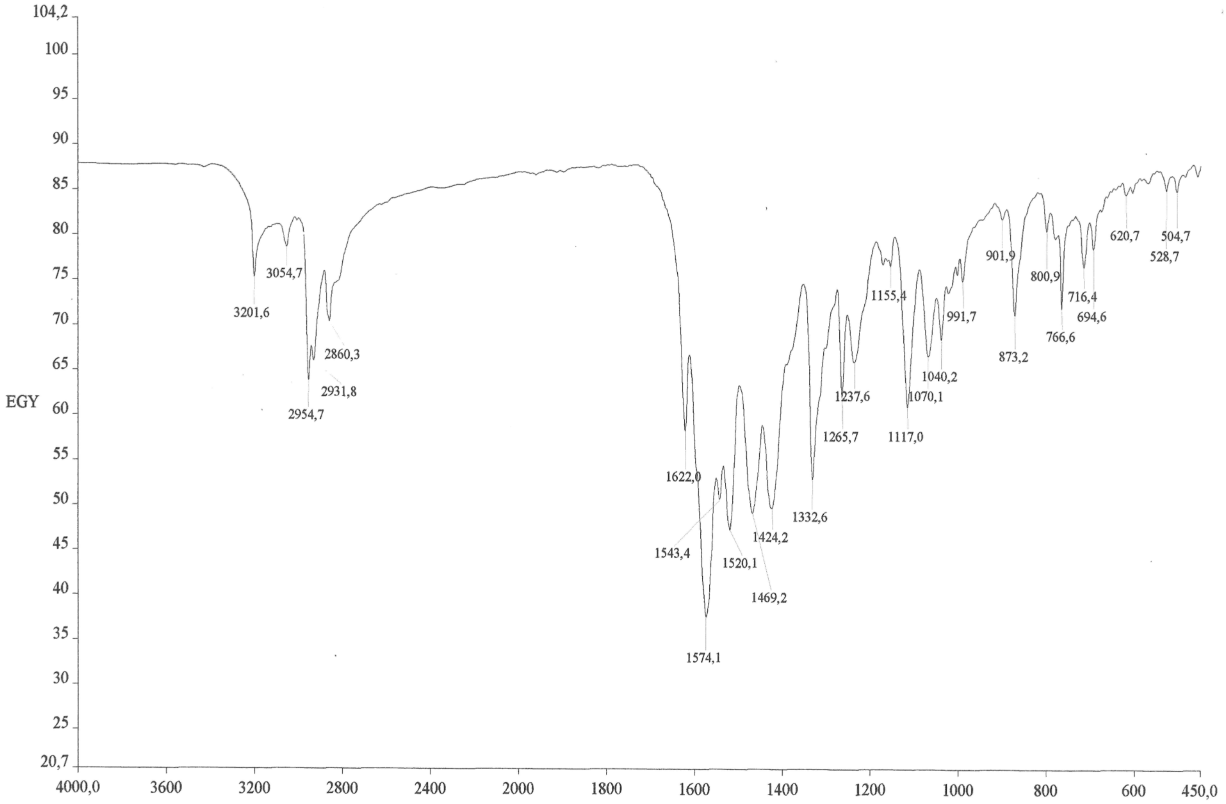

Figure S38. ^{1}H-NMR (200 MHz, $CDCl_3$) spectrum of compound **3e**

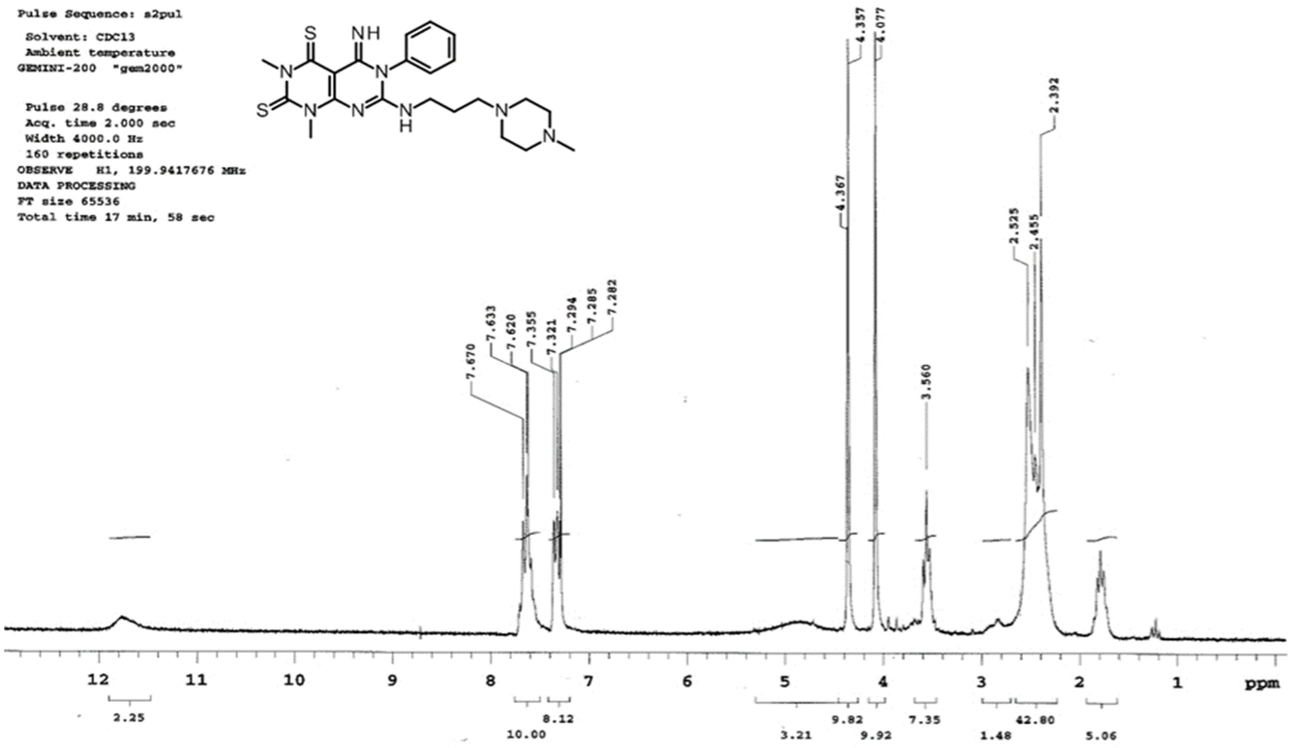

Figure S39. ^{13}C-NMR (101 MHz, $CDCl_3$) spectrum of compound **3e**

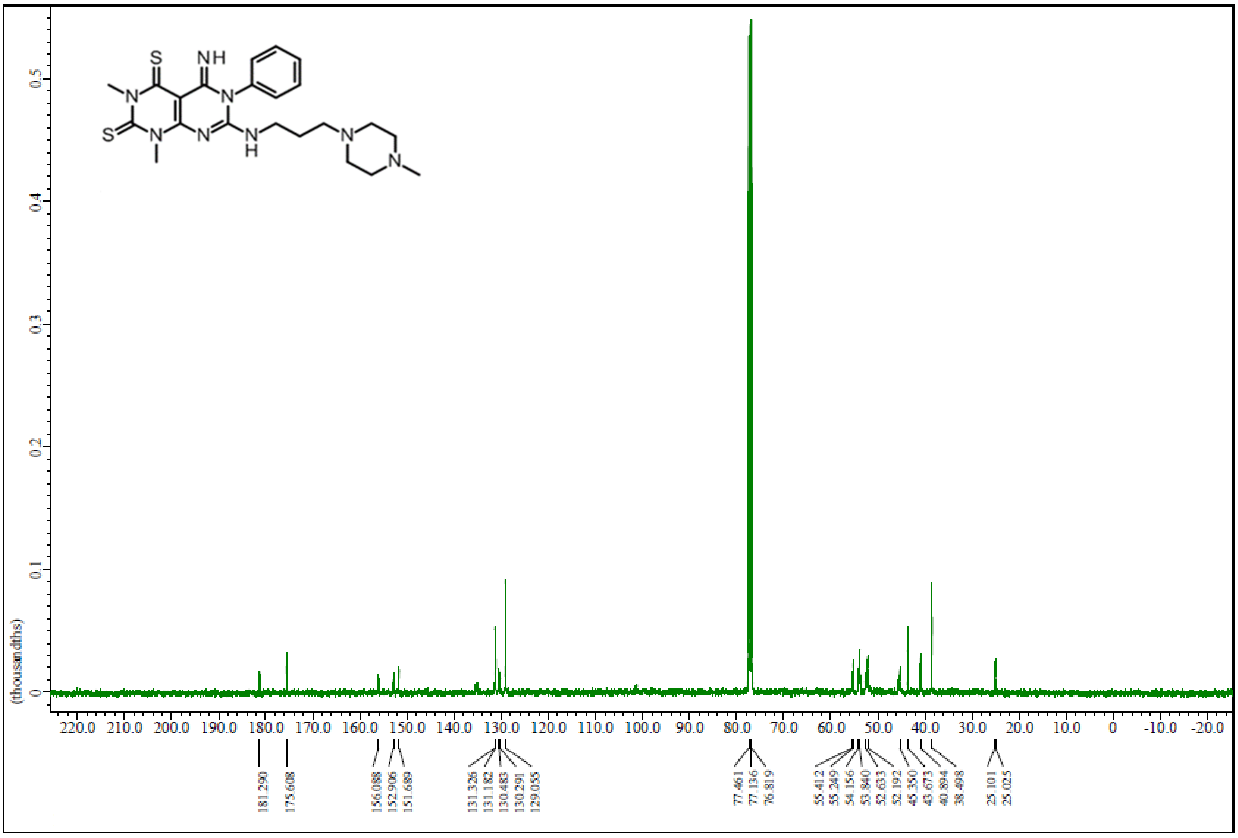

Figure S40. IR (KBr) spectrum of compound **3e**

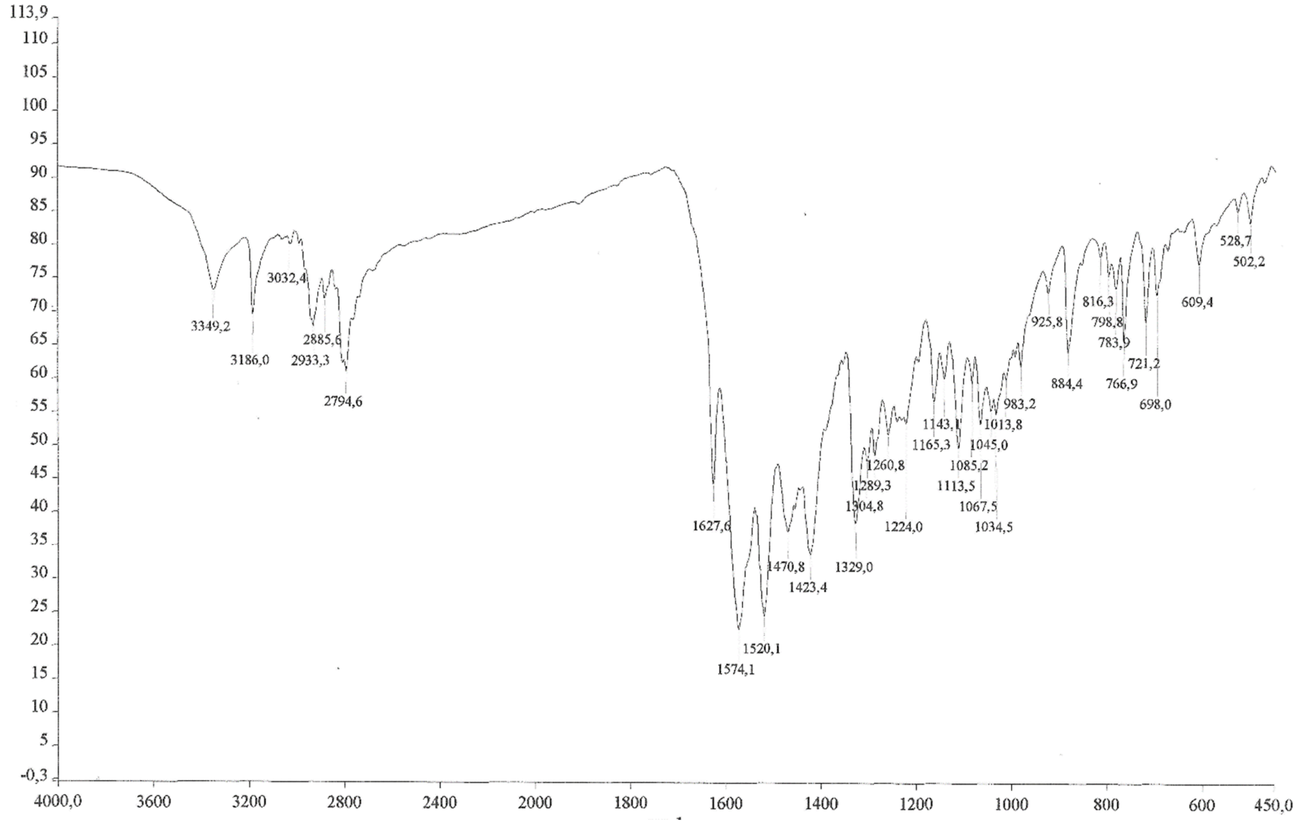

Figure S41. ^{1}H-NMR (200 MHz, $CDCl_3$) spectrum of compound **4a**

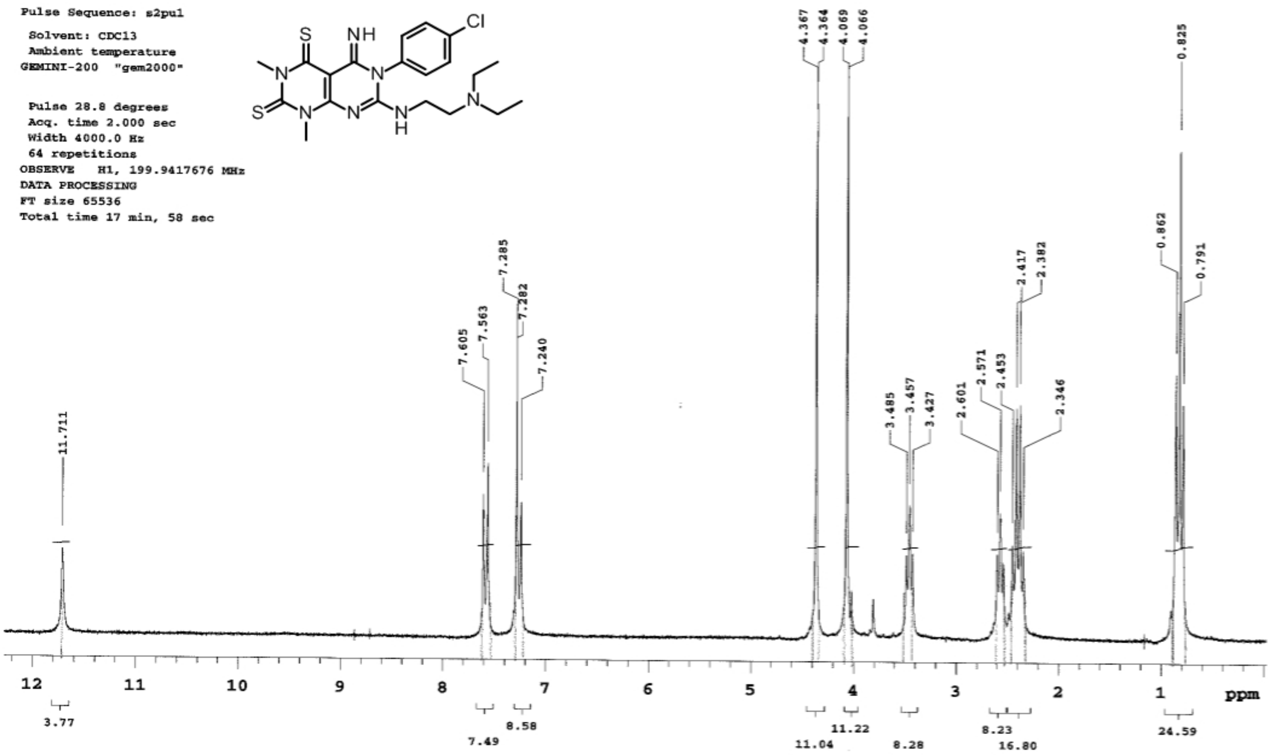

Figure S42. ^{13}C-NMR (101 MHz, $CDCl_3$) spectrum of compound **4a**

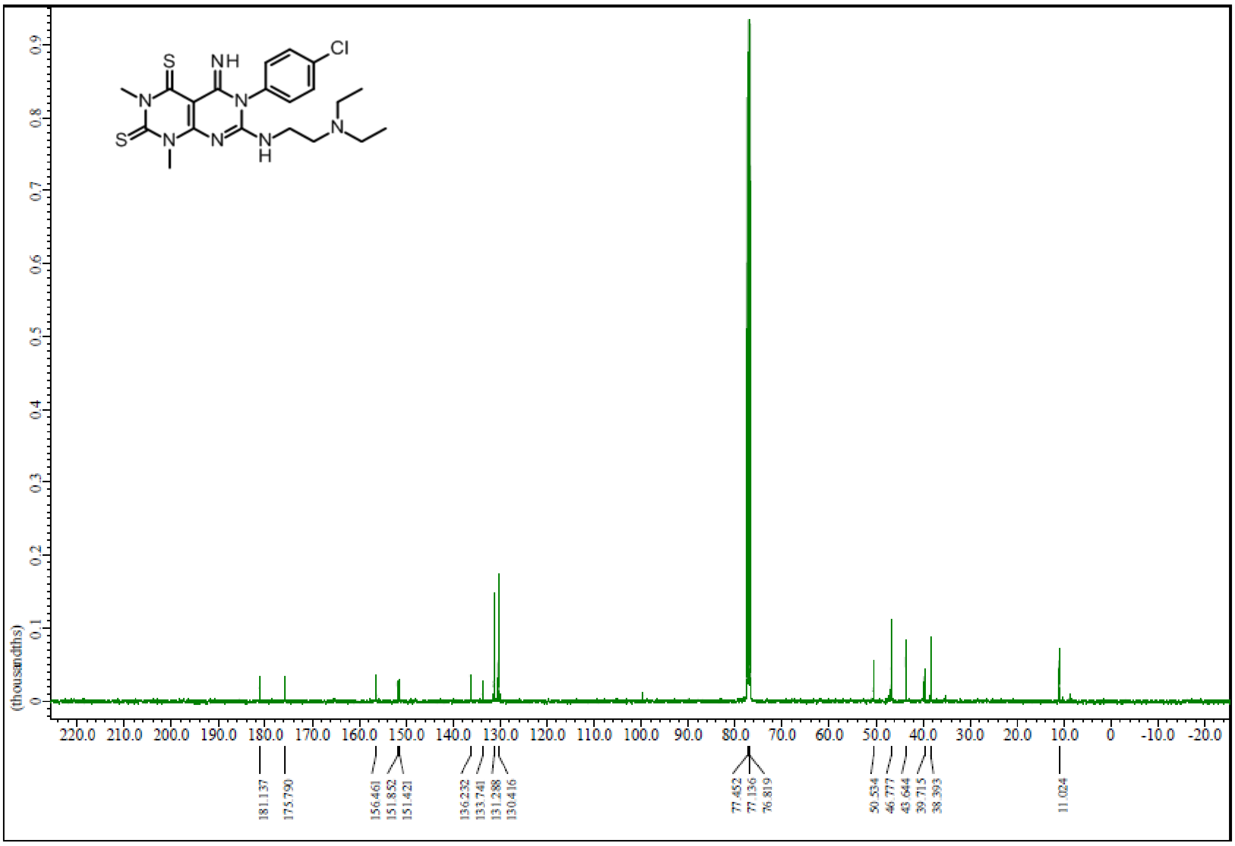

Figure S43. IR (KBr) spectrum of compound **4a**

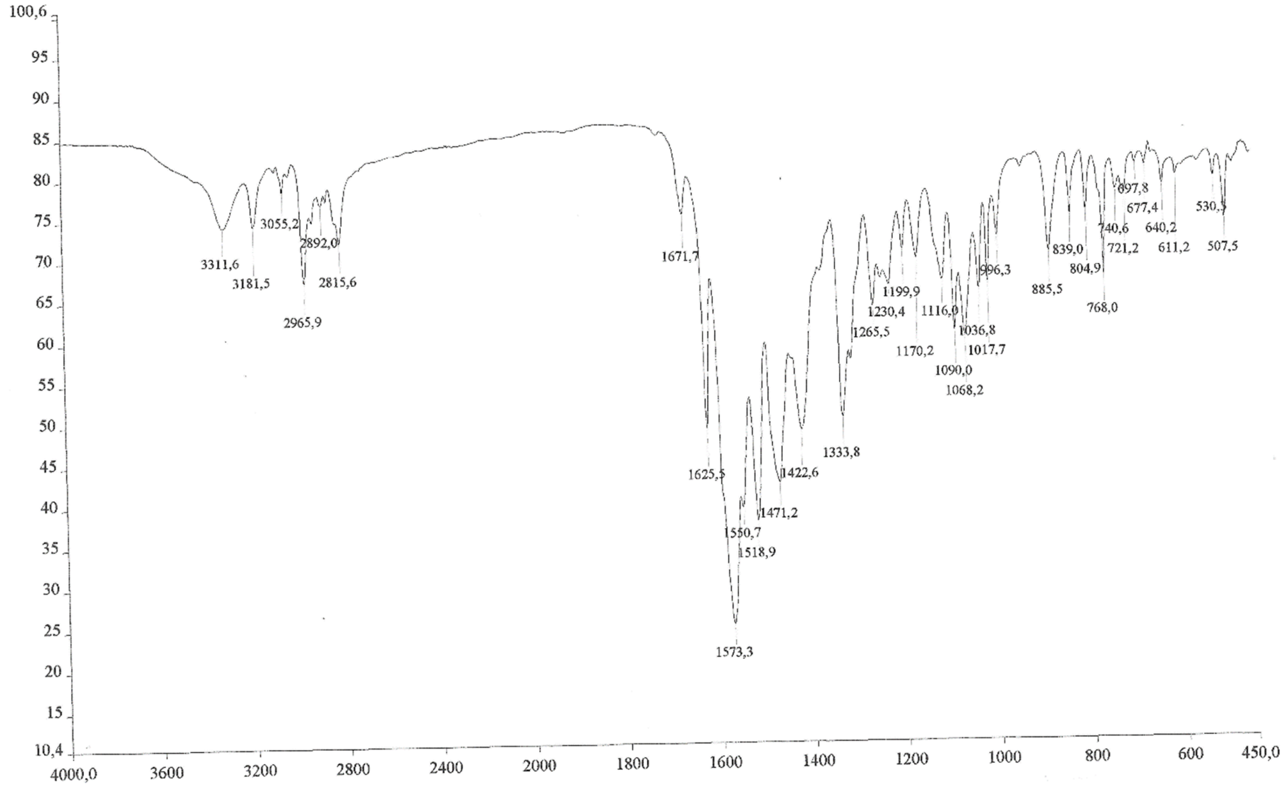

Figure S44. ^{1}H-NMR (200 MHz, $CDCl_3$) spectrum of compound **4b**

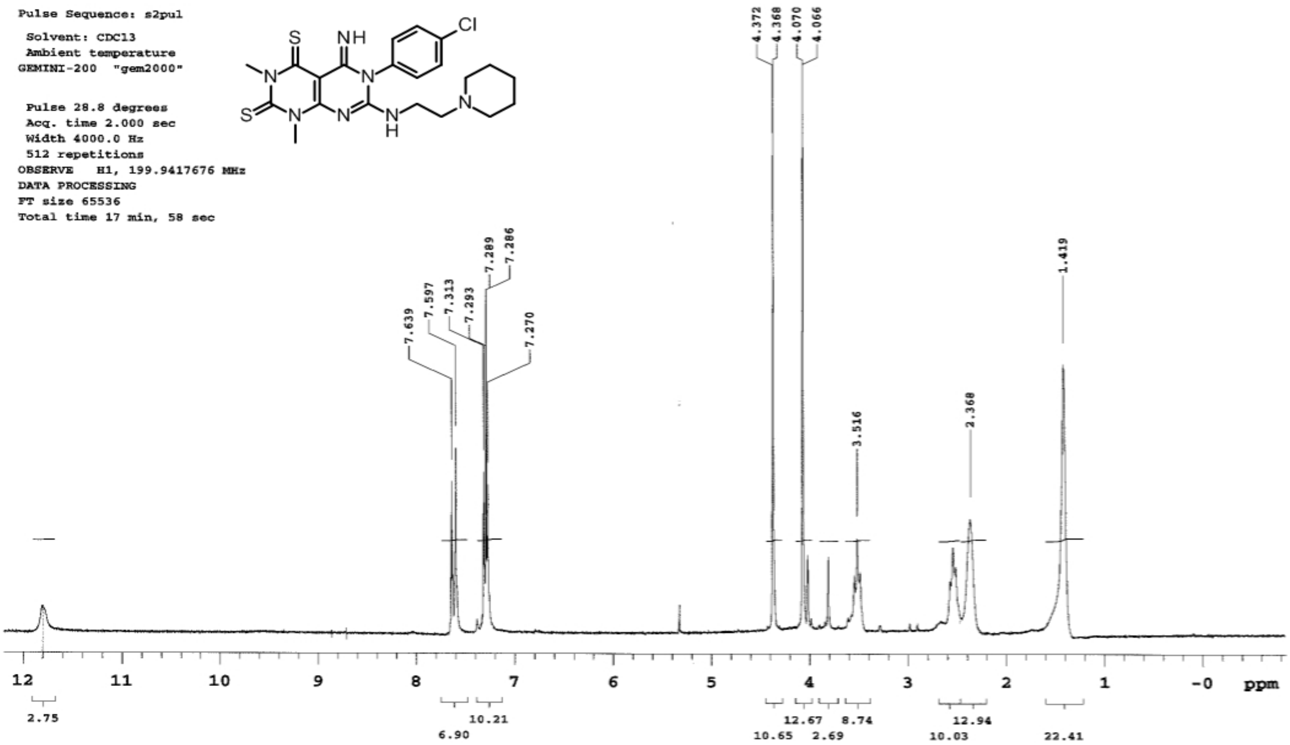

Figure S45. ^{13}C-NMR (101 MHz, $CDCl_3$) spectrum of compound **4b**

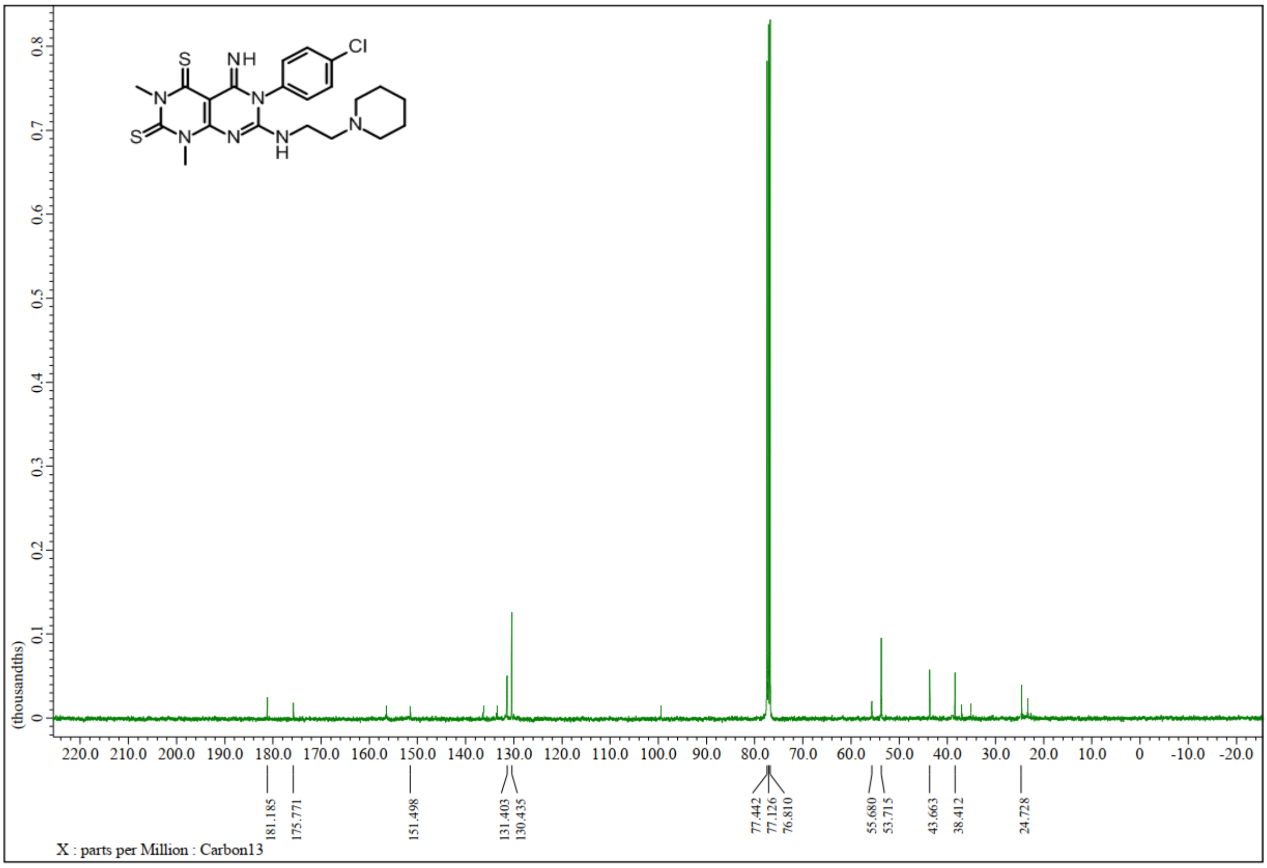

Figure S46. IR (KBr) spectrum of compound **4b**

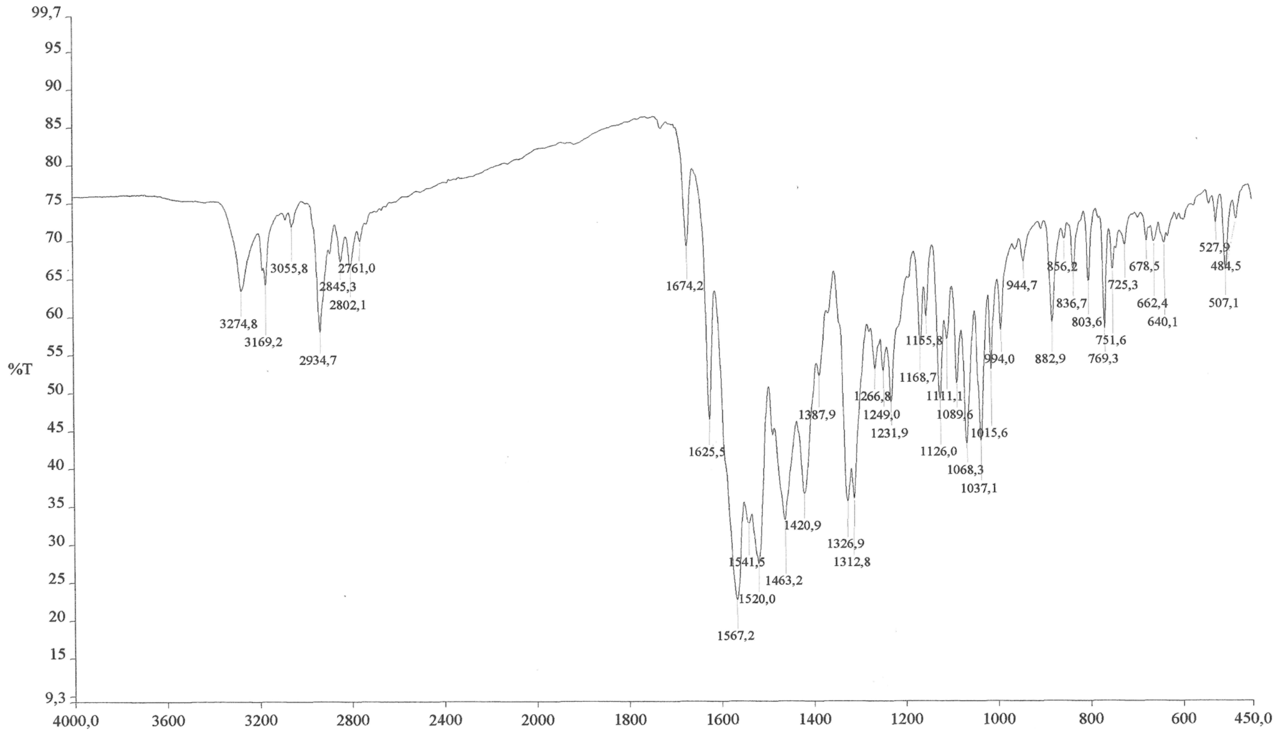

Figure S47. ^{1}H-NMR (200 MHz, $CDCl_3$) spectrum of compound **4c**

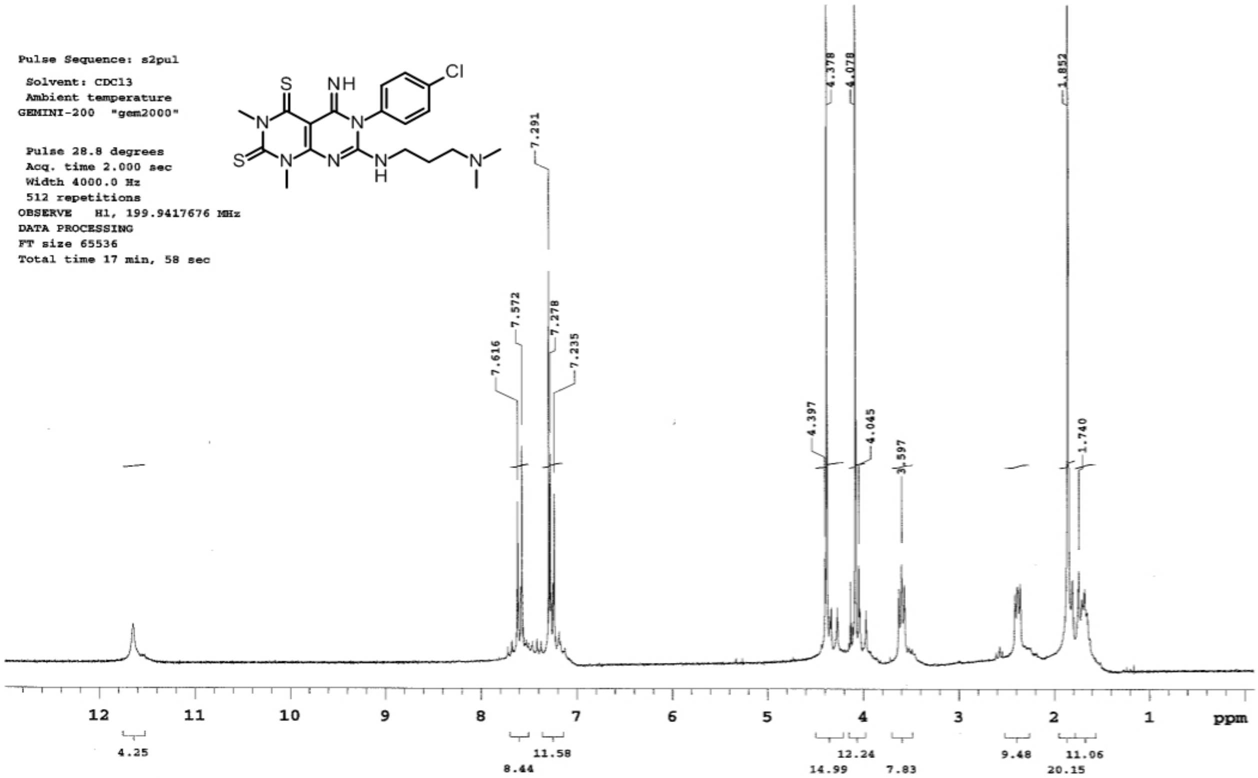

Figure S48. ^{13}C-NMR (101 MHz, $CDCl_3$) spectrum of compound **4c**

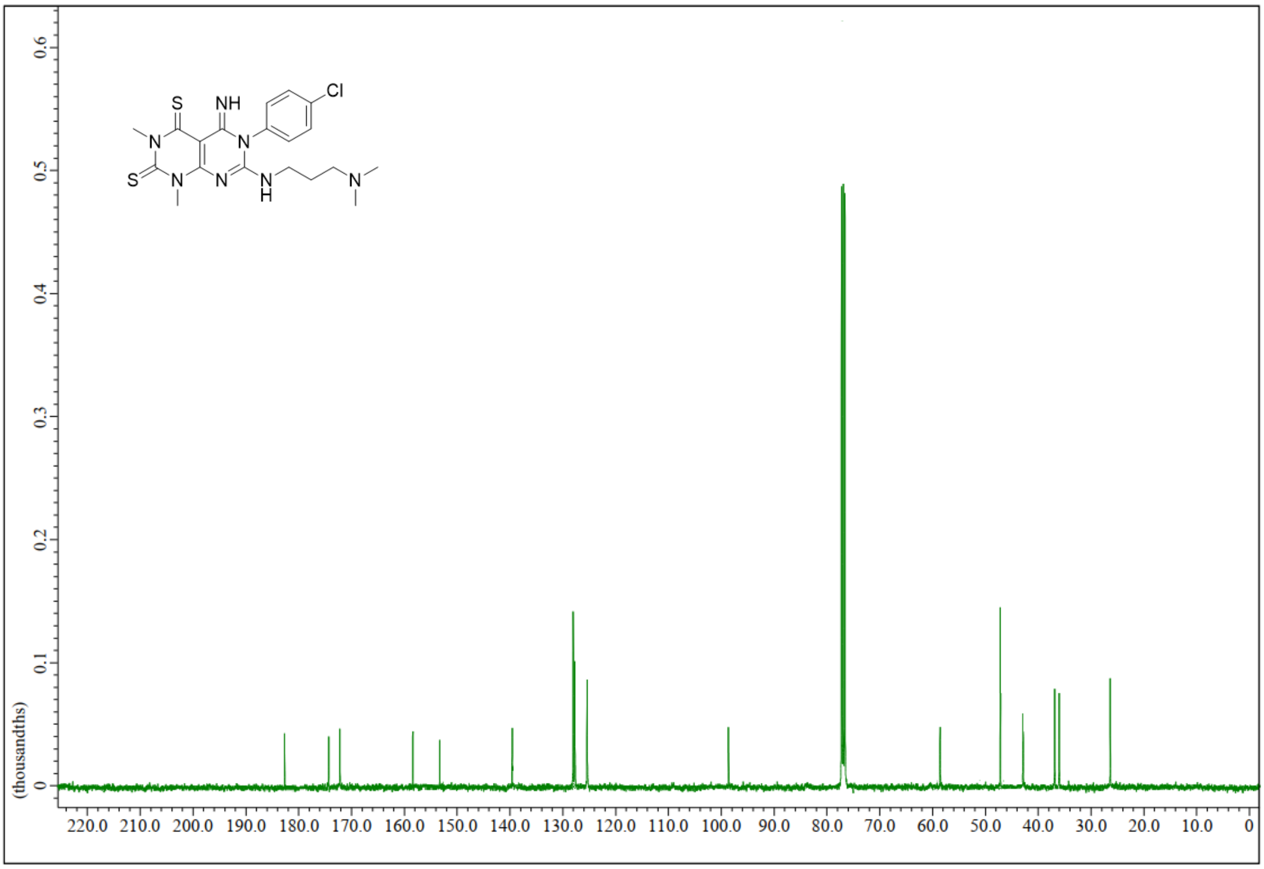

Figure S49. IR (KBr) spectrum of compound **4c**

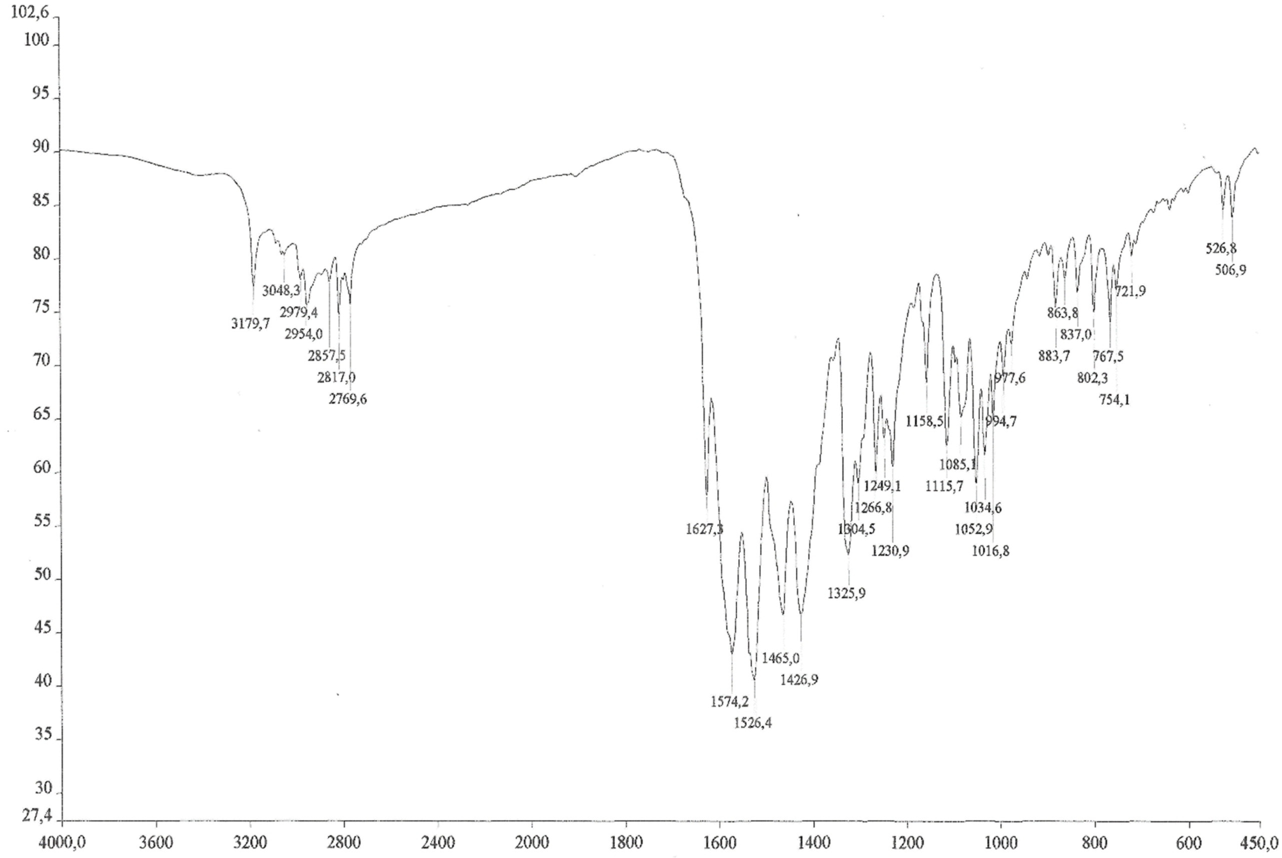

Figure S50. ^{1}H-NMR (200 MHz, CDCl$_3$) spectrum of compound **4d**

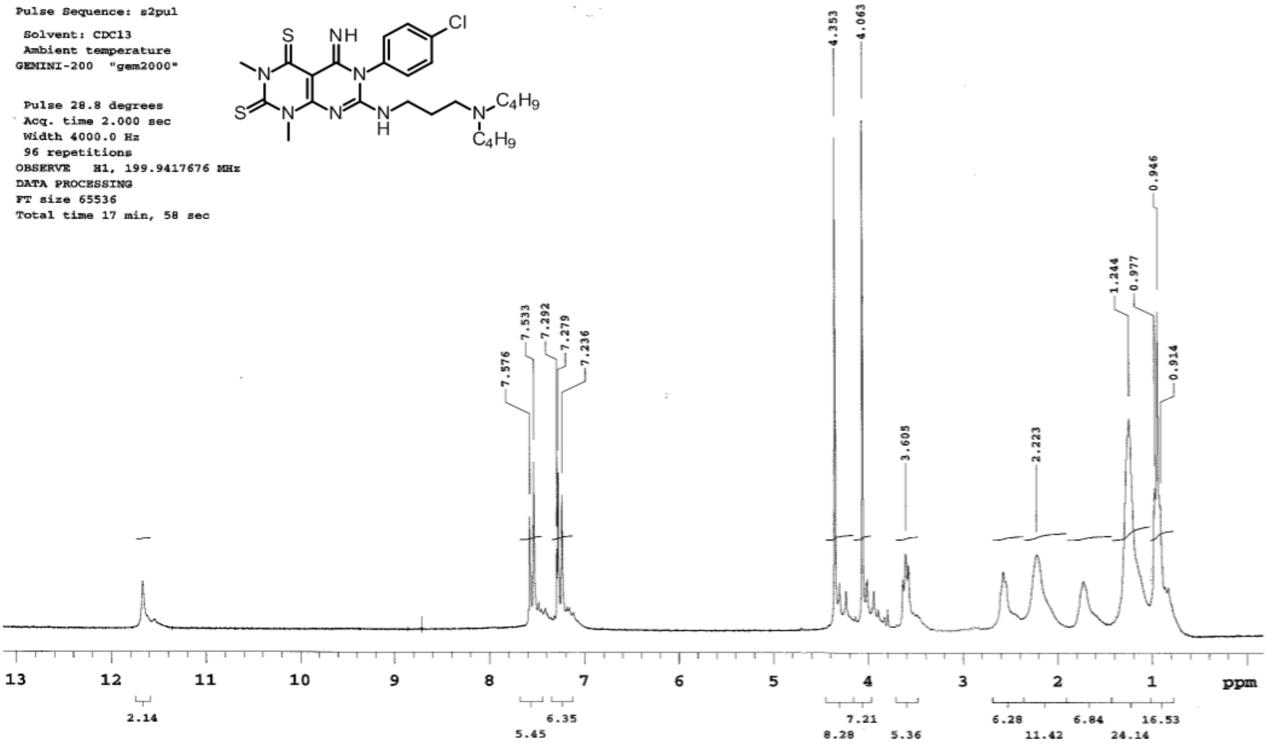

Figure S51. ^{13}C-NMR (101 MHz, CDCl$_3$) spectrum of compound **4d**

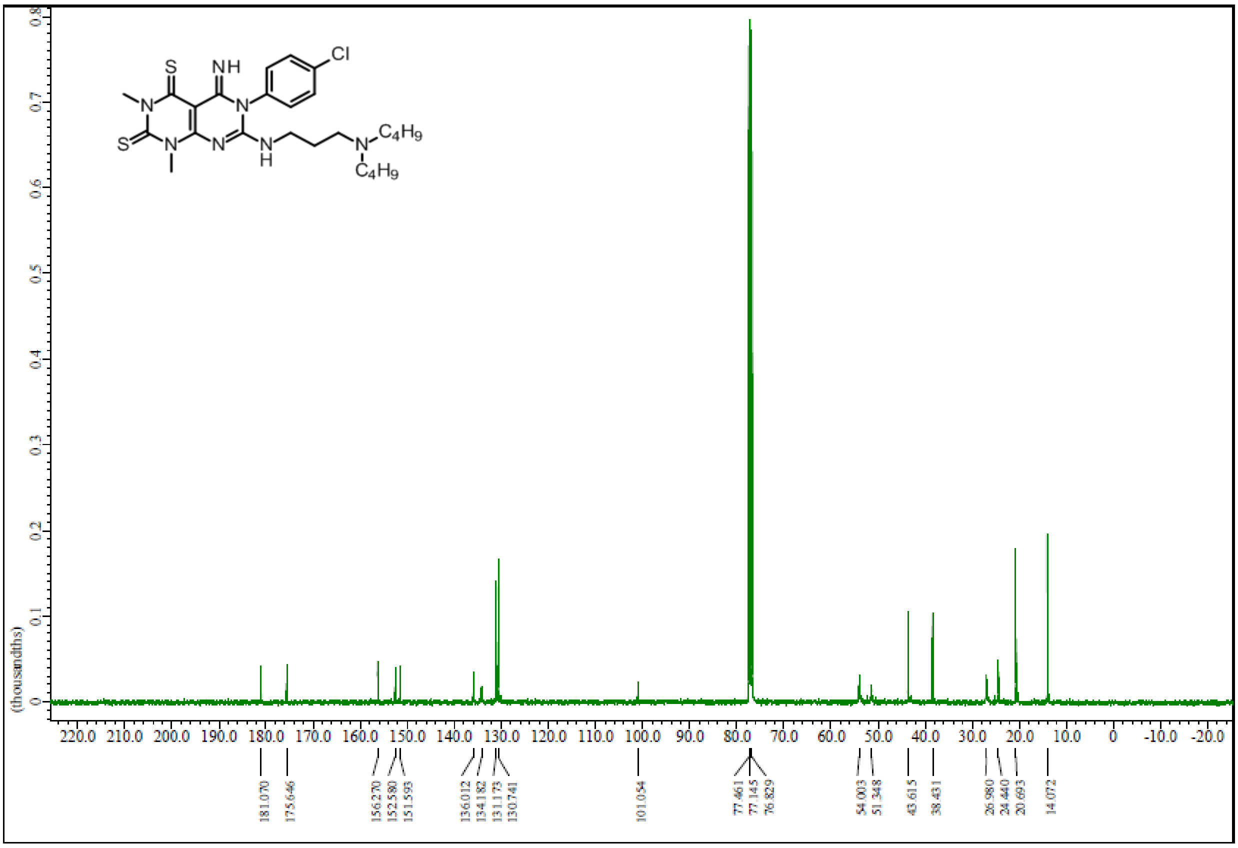

Figure S52. IR (KBr) spectrum of compound **4d**

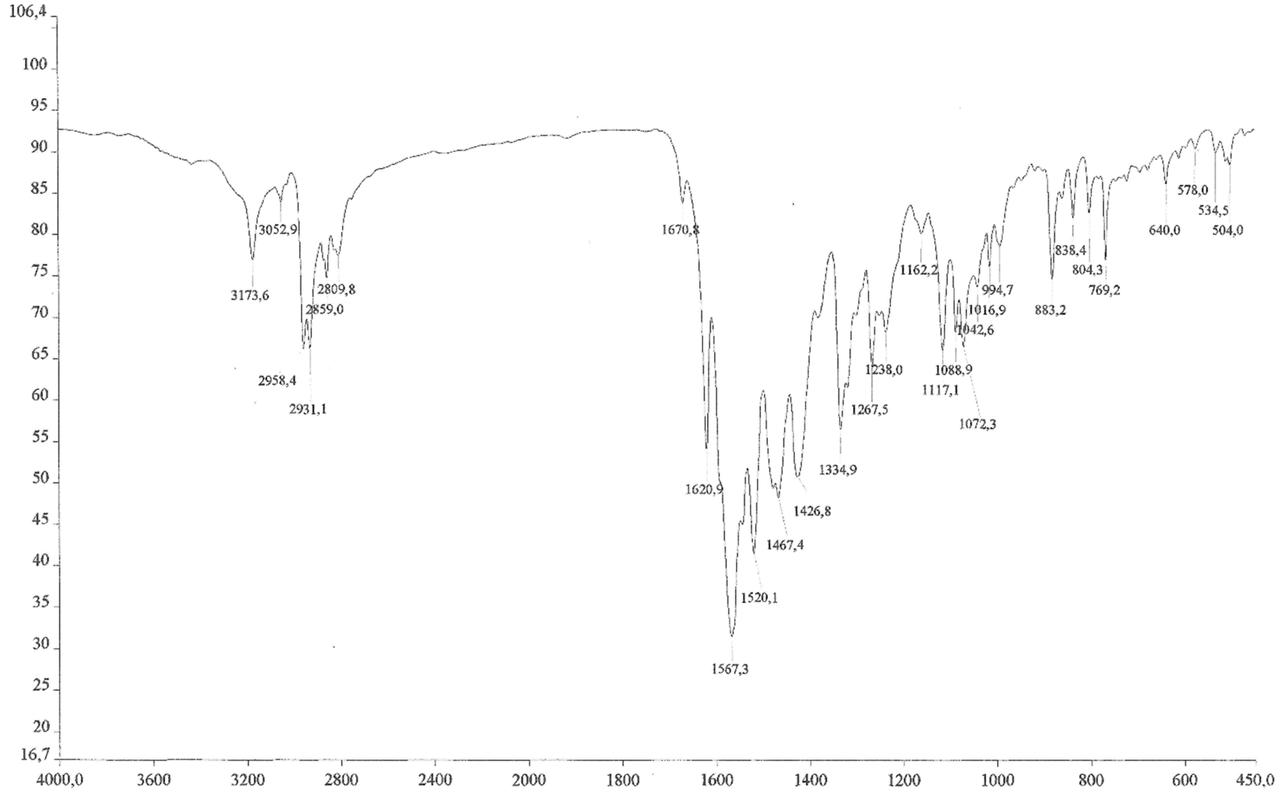

Figure S53. ^{1}H-NMR (200 MHz, $CDCl_3$) spectrum of compound **4e**

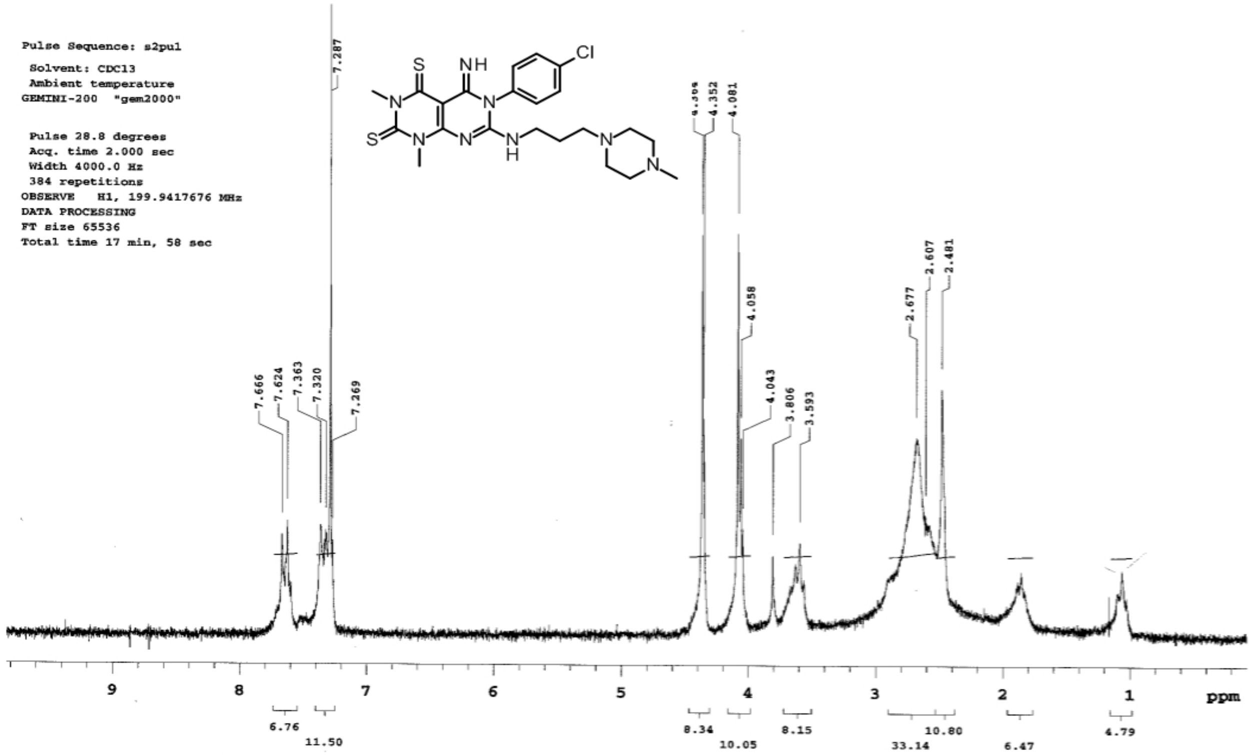

Figure S54. ^{13}C-NMR (101 MHz, $CDCl_3$) spectrum of compound **4e**

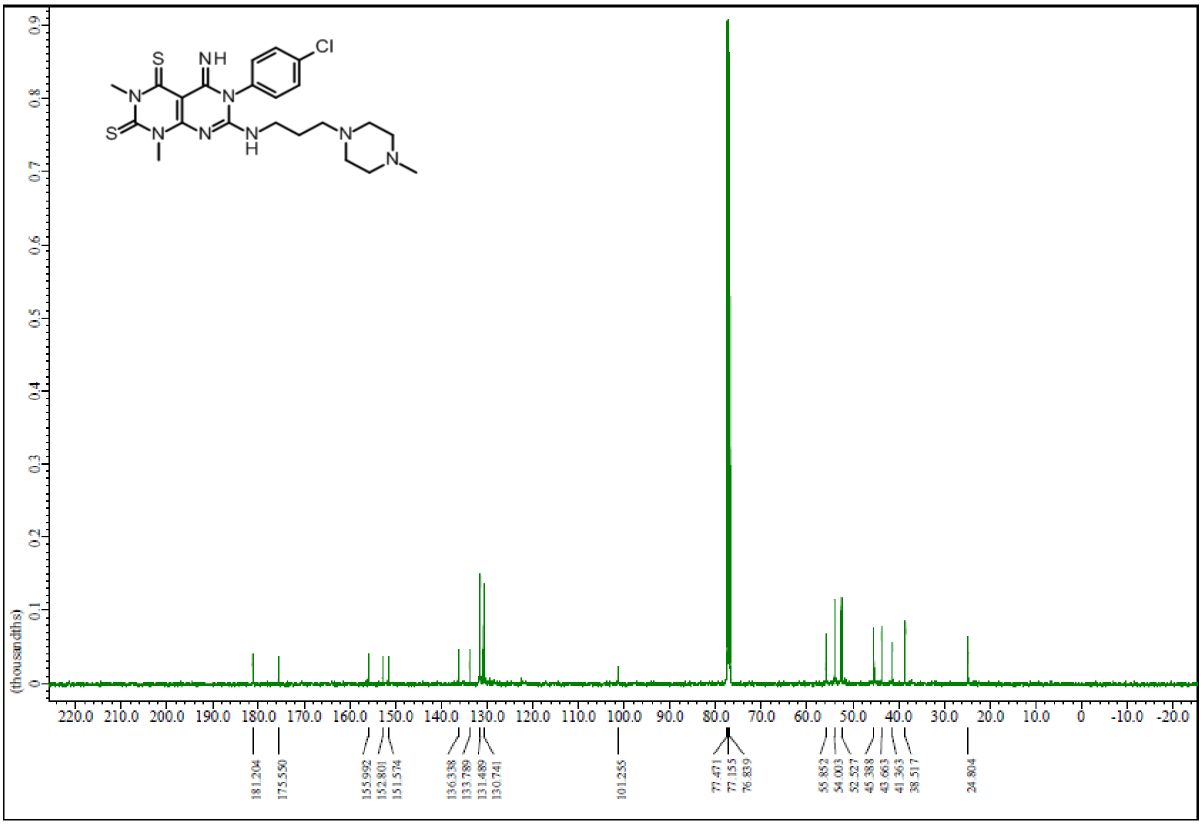

Figure S55. IR (KBr) spectrum of compound **4e**

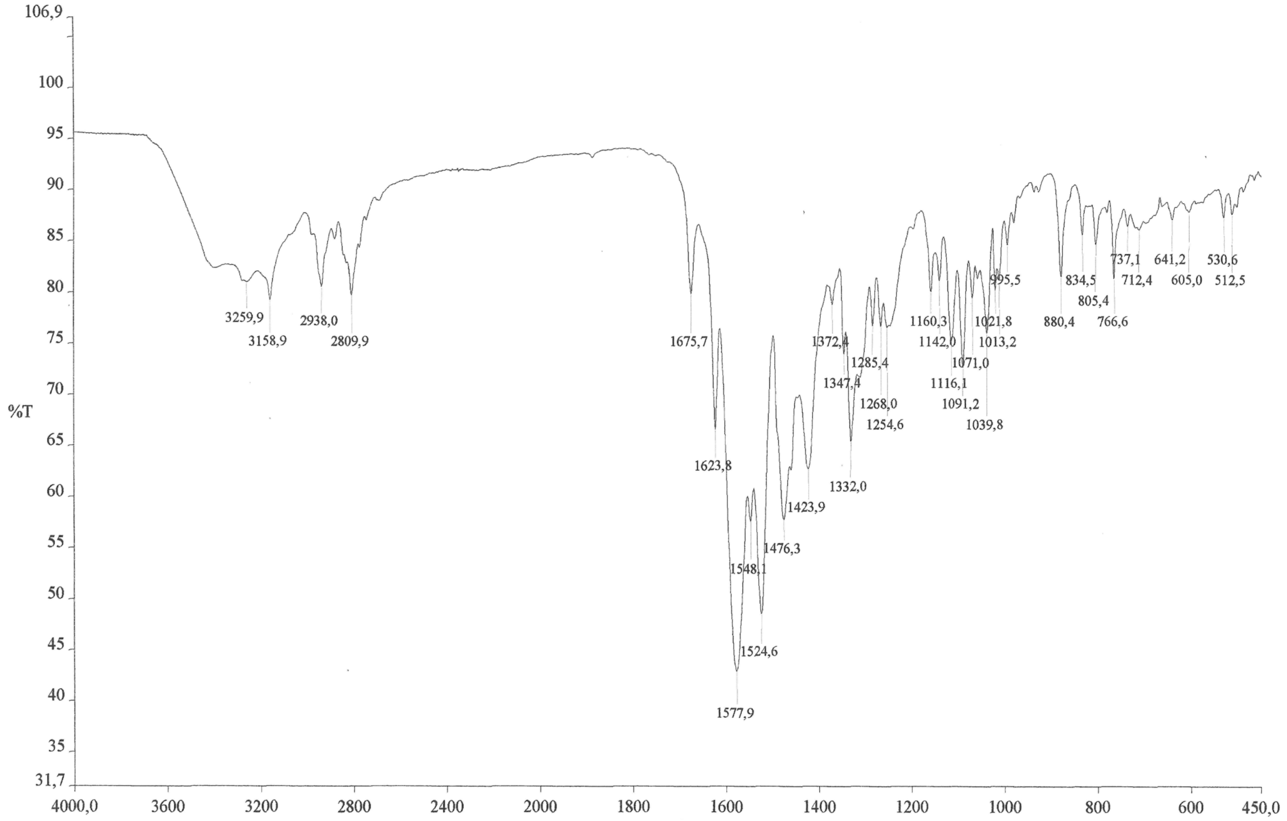

Figure S56. Mass spectrum of compound **4e**

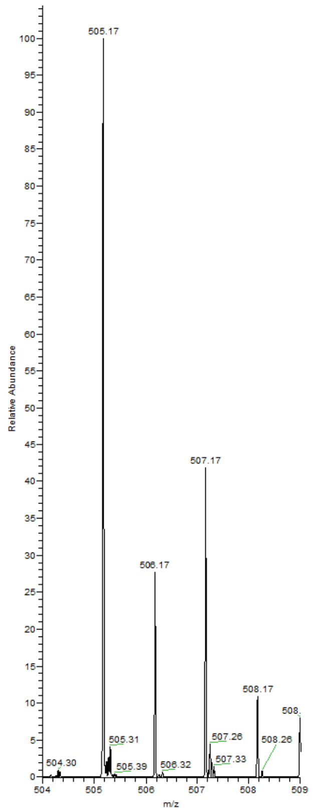

Figure S57. ^{1}H-NMR (200 MHz, $CDCl_3$) spectrum of compound **5a**

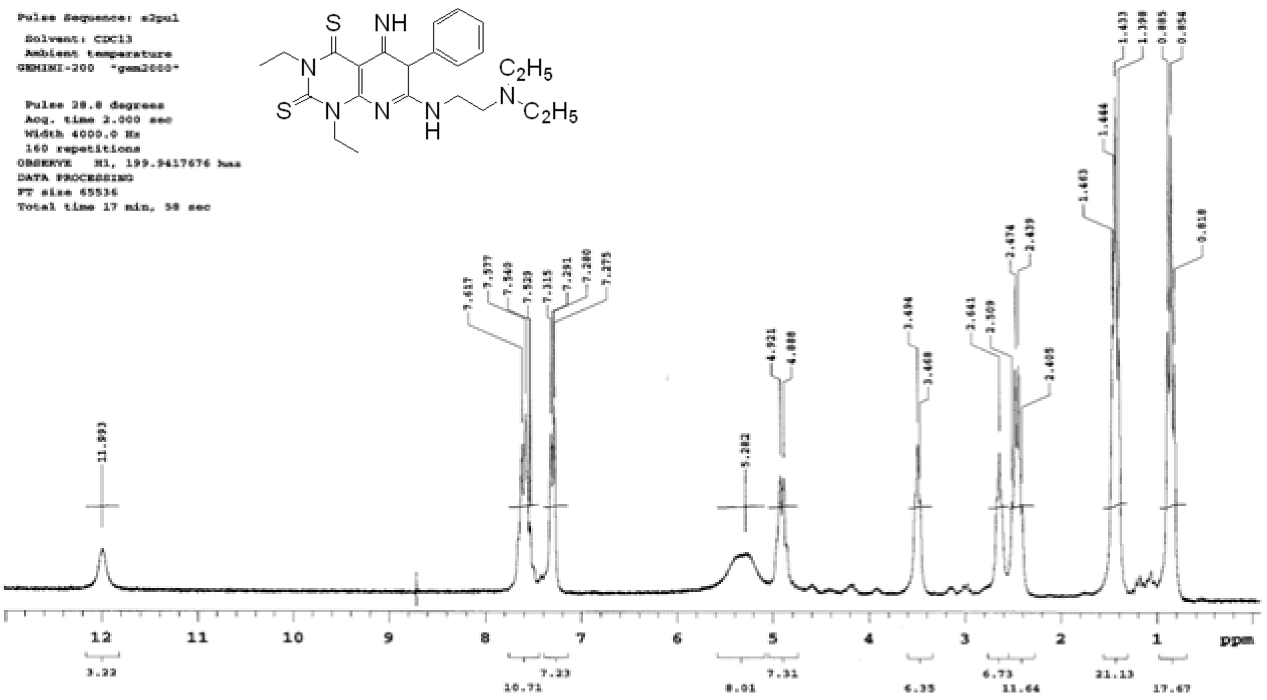

Figure S58. ^{13}C-NMR (101 MHz, $CDCl_3$) spectrum of compound **5a**

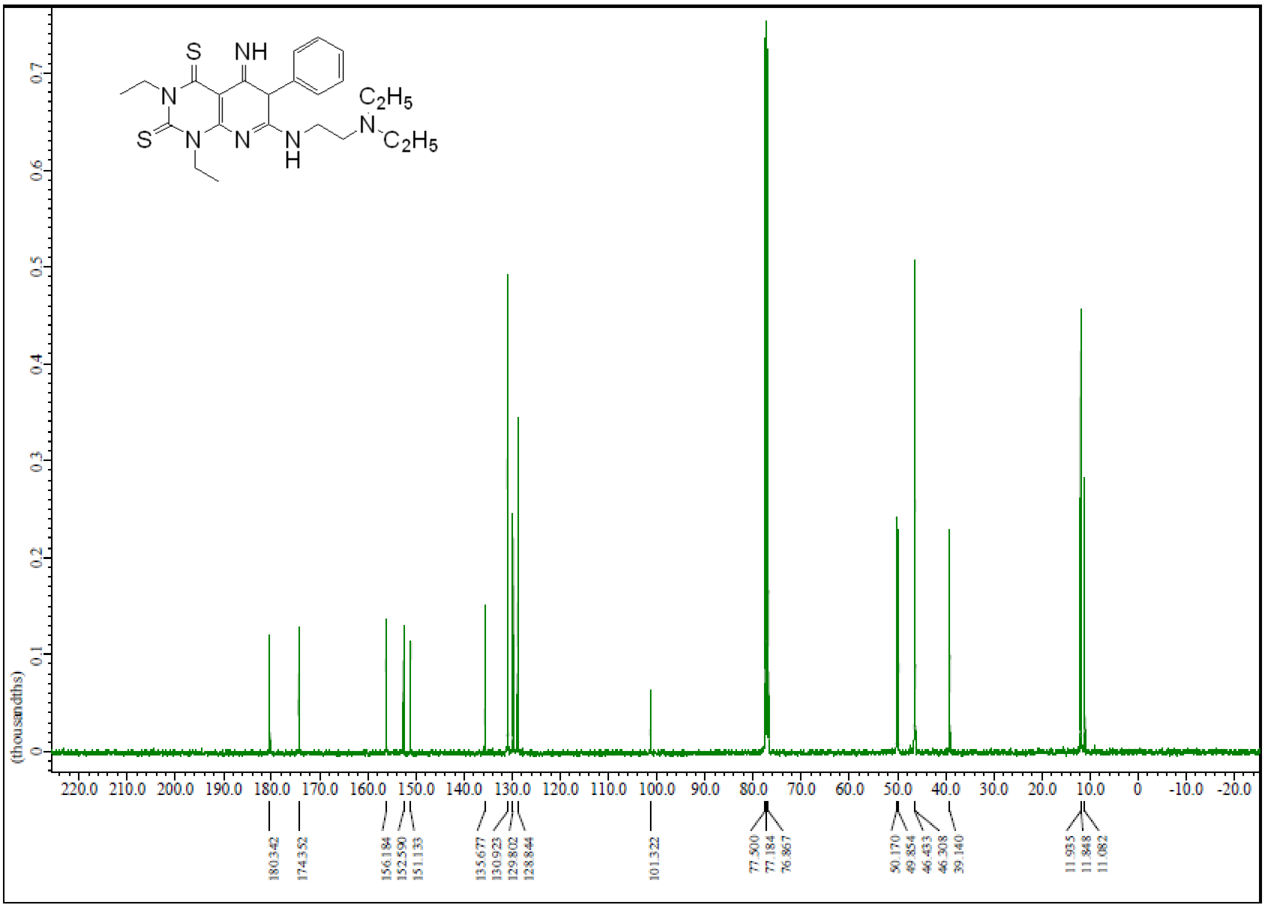

Figure S59. IR (KBr) spectrum of compound **5a**

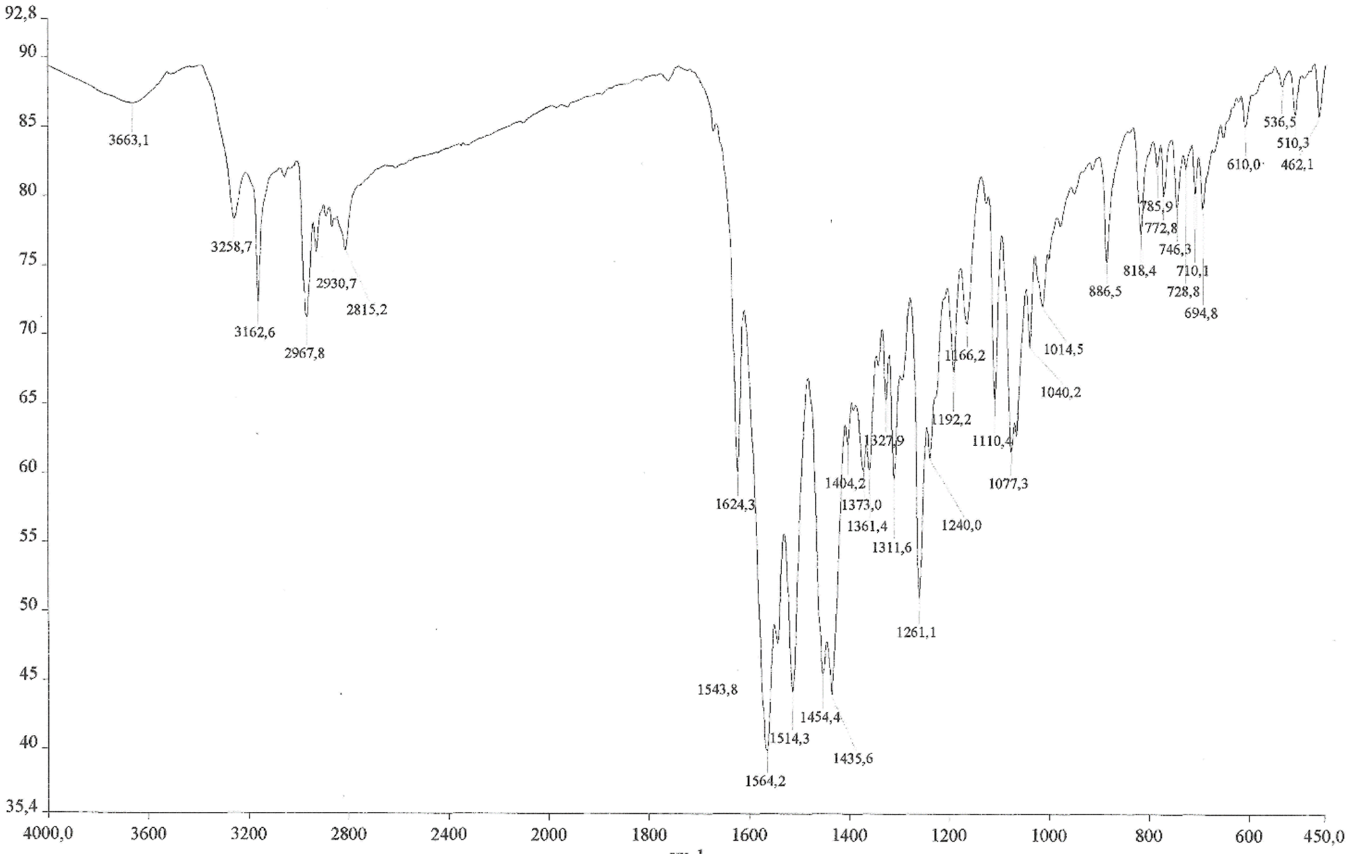

Figure S60. ^{1}H-NMR (200 MHz, $CDCl_3$) spectrum of compound **5b**

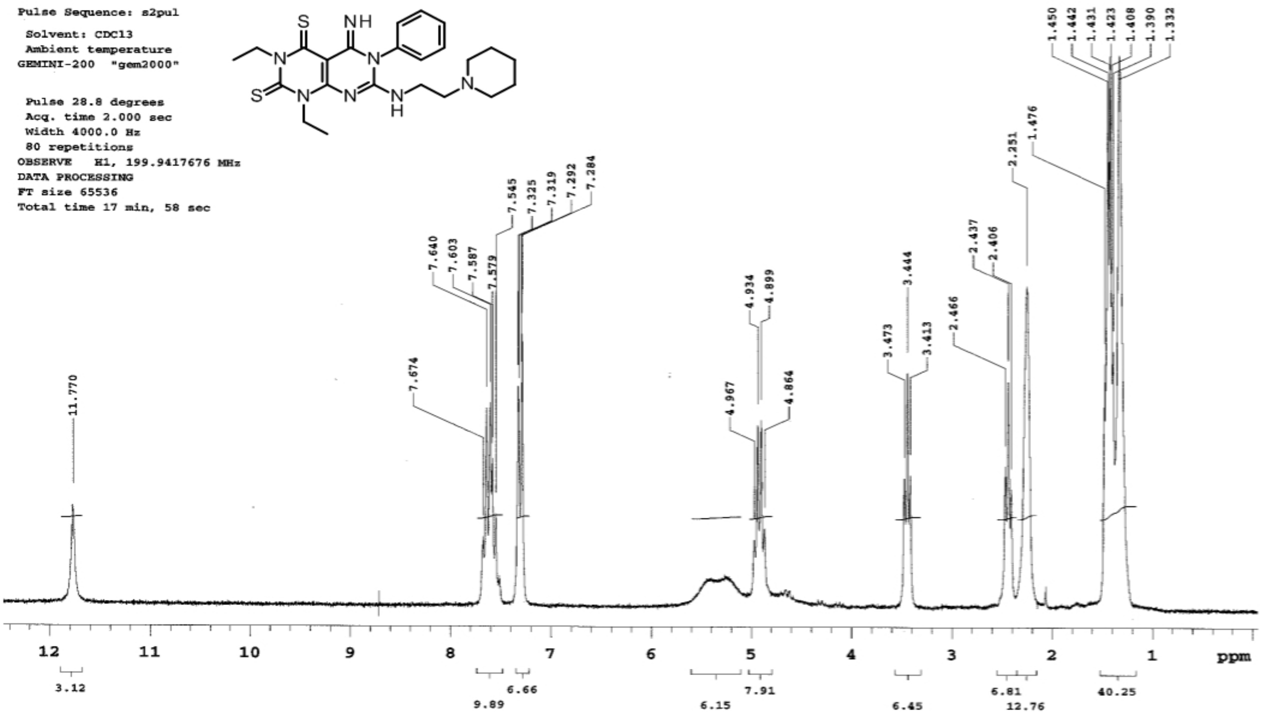

Figure S61. ^{13}C-NMR (101 MHz, $CDCl_3$) spectrum of compound **5b**

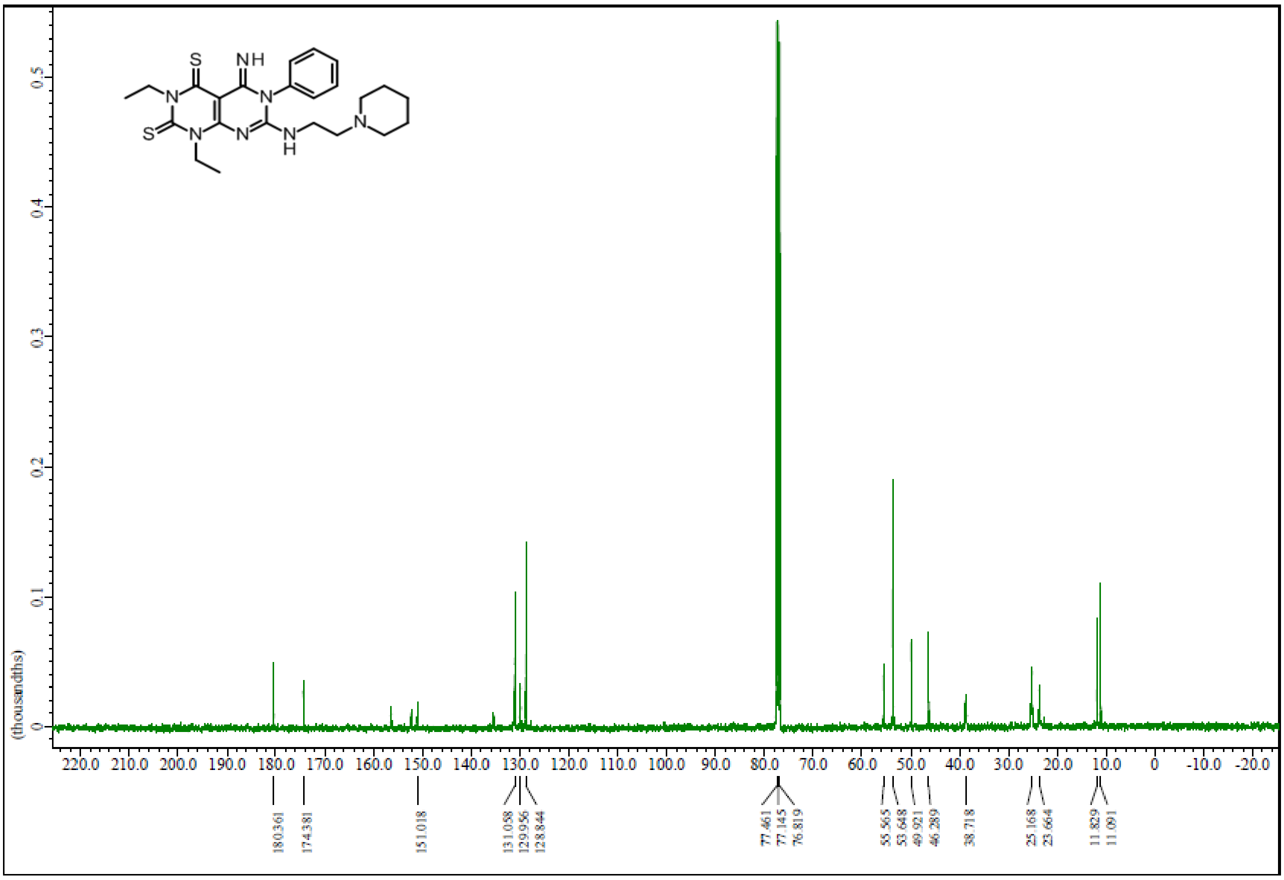

Figure S62. IR (KBr) spectrum of compound **5b**

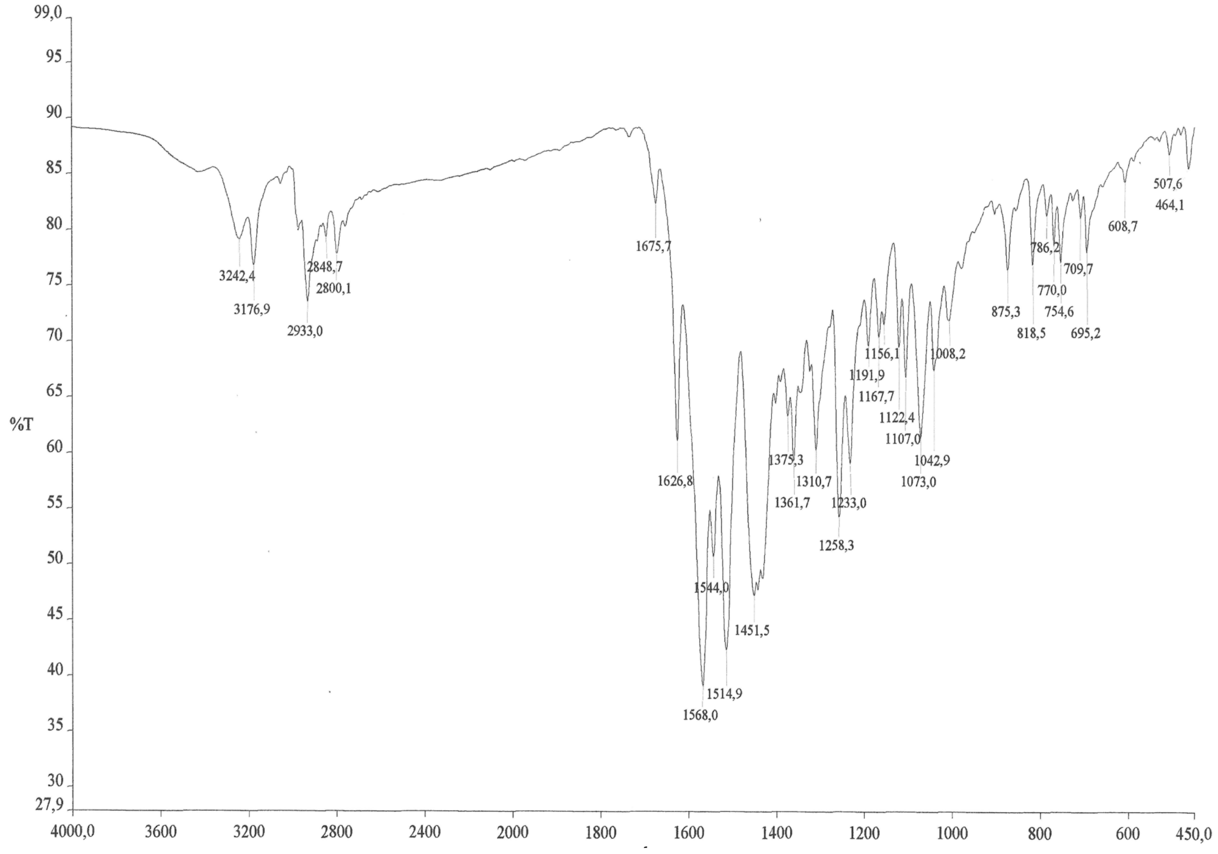

Figure S63. ^{1}H-NMR (200 MHz, $CDCl_3$) spectrum of compound **5c**

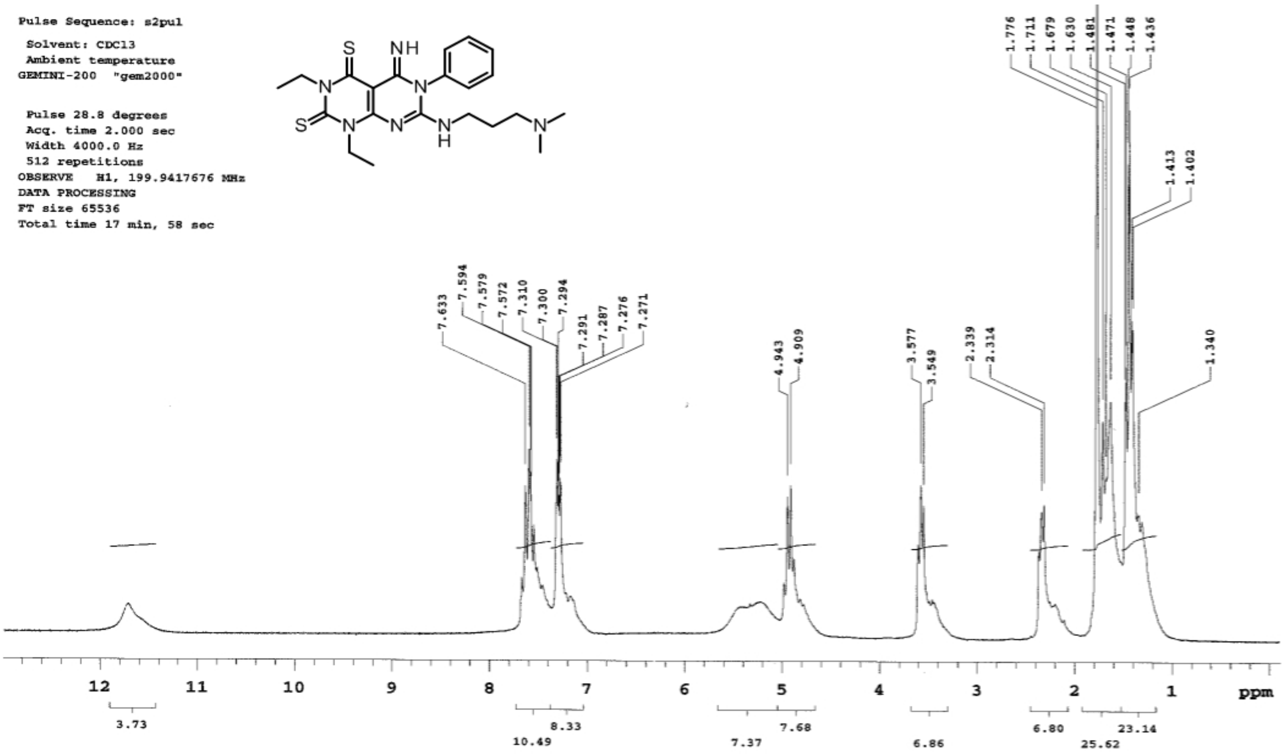

Figure S64. ^{13}C-NMR (101 MHz, $CDCl_3$) spectrum of compound **5c**

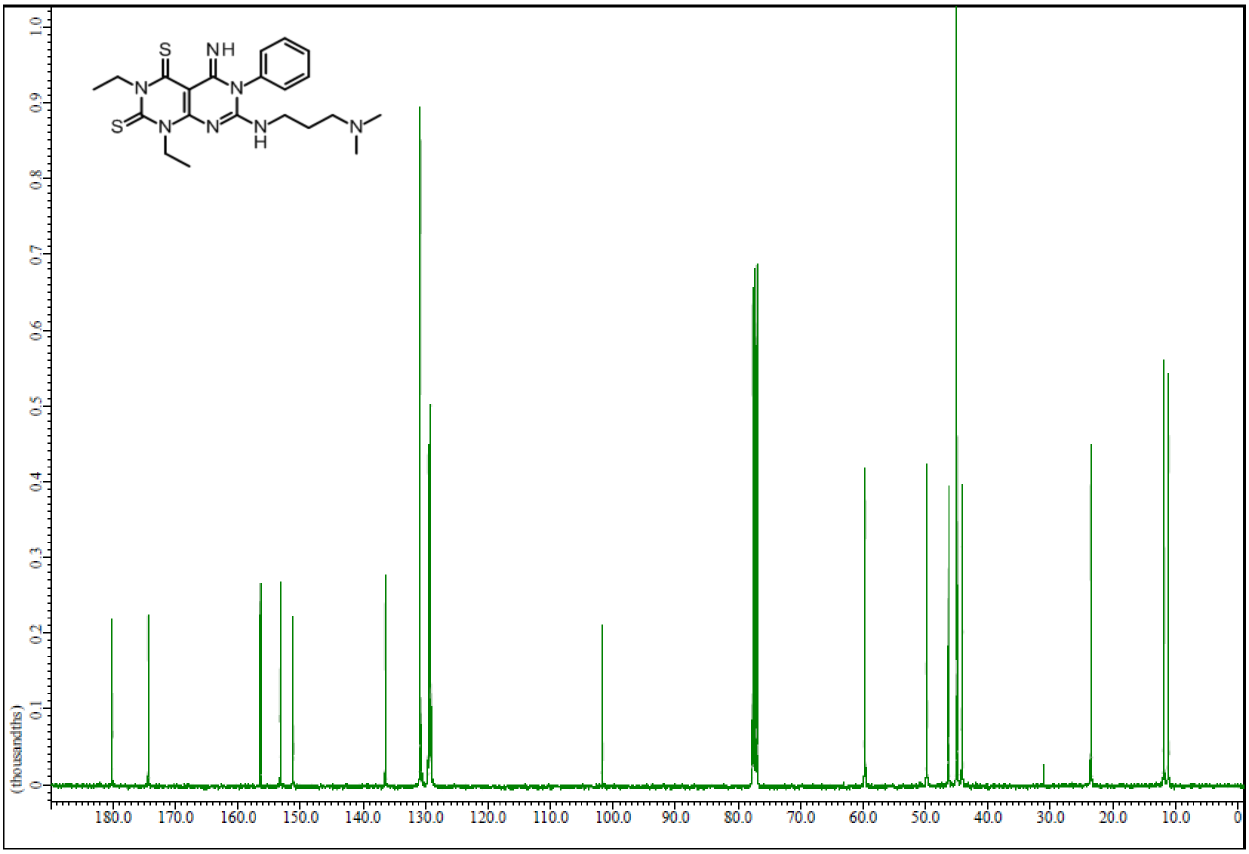

Figure S65. IR (KBr) spectrum of compound **5c**

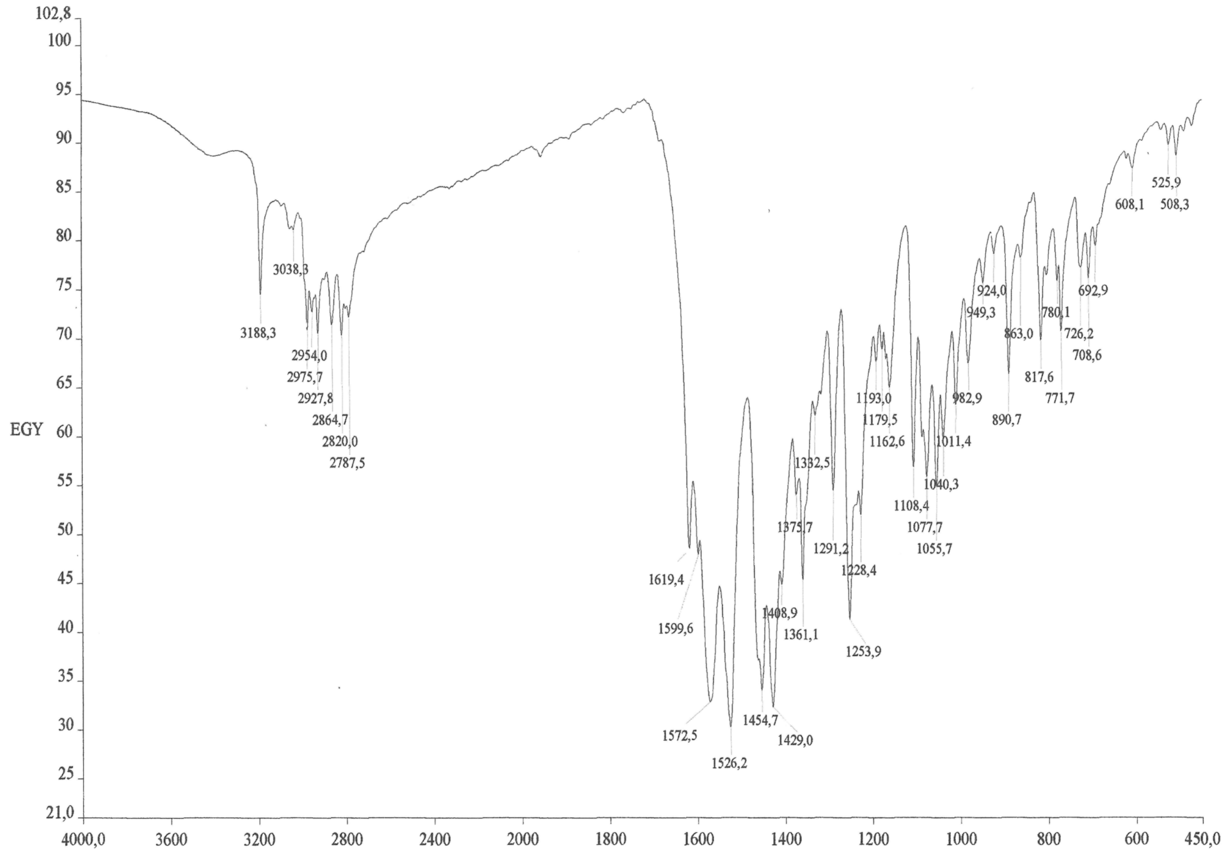

Figure S66. ^{1}H-NMR (200 MHz, $CDCl_3$) spectrum of compound **5d**

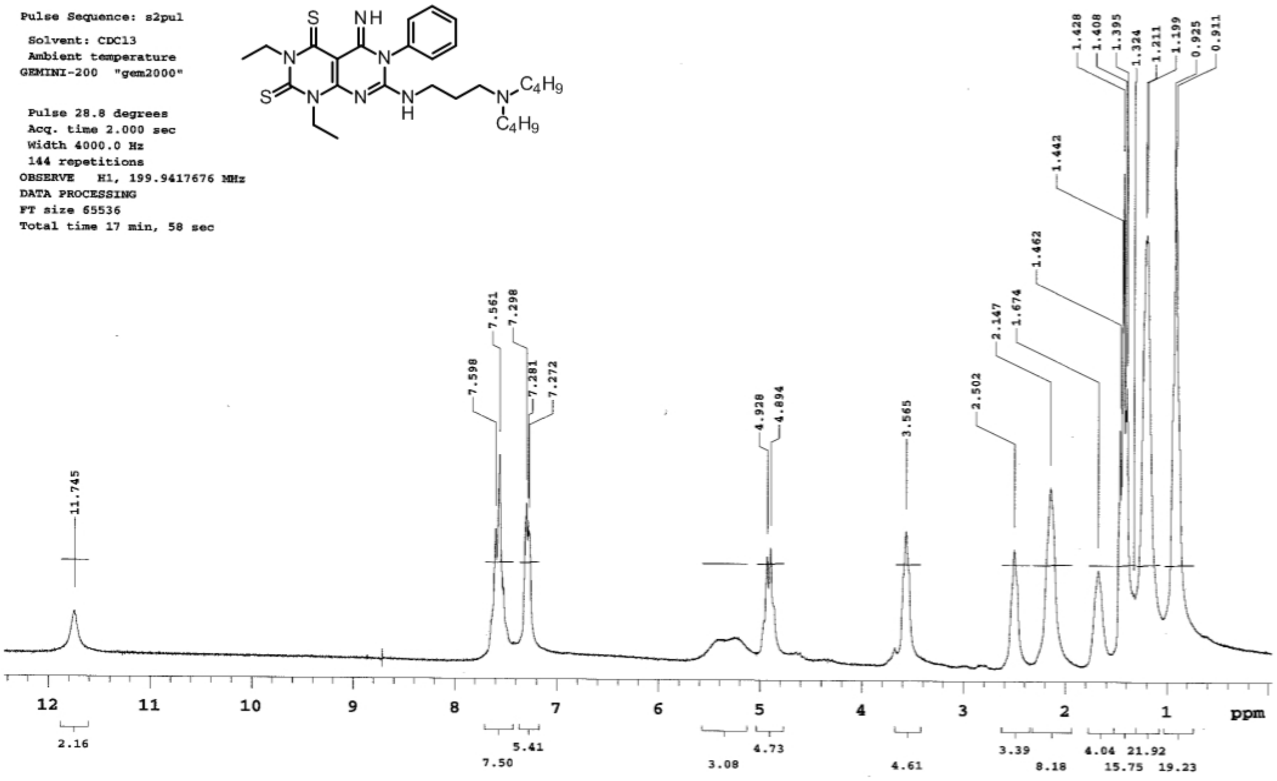

Figure S67. ^{13}C-NMR (101 MHz, $CDCl_3$) spectrum of compound **5d**

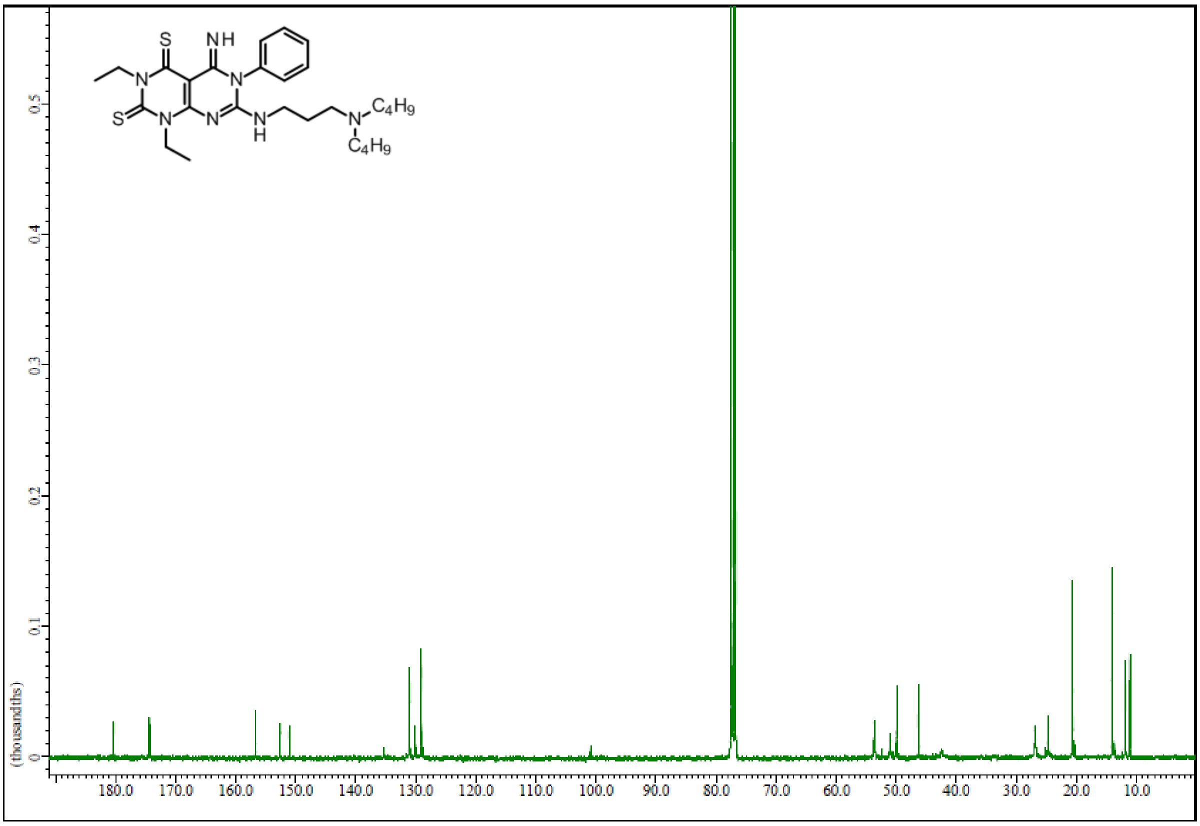

Figure S68. IR (KBr) spectrum of compound **5d**

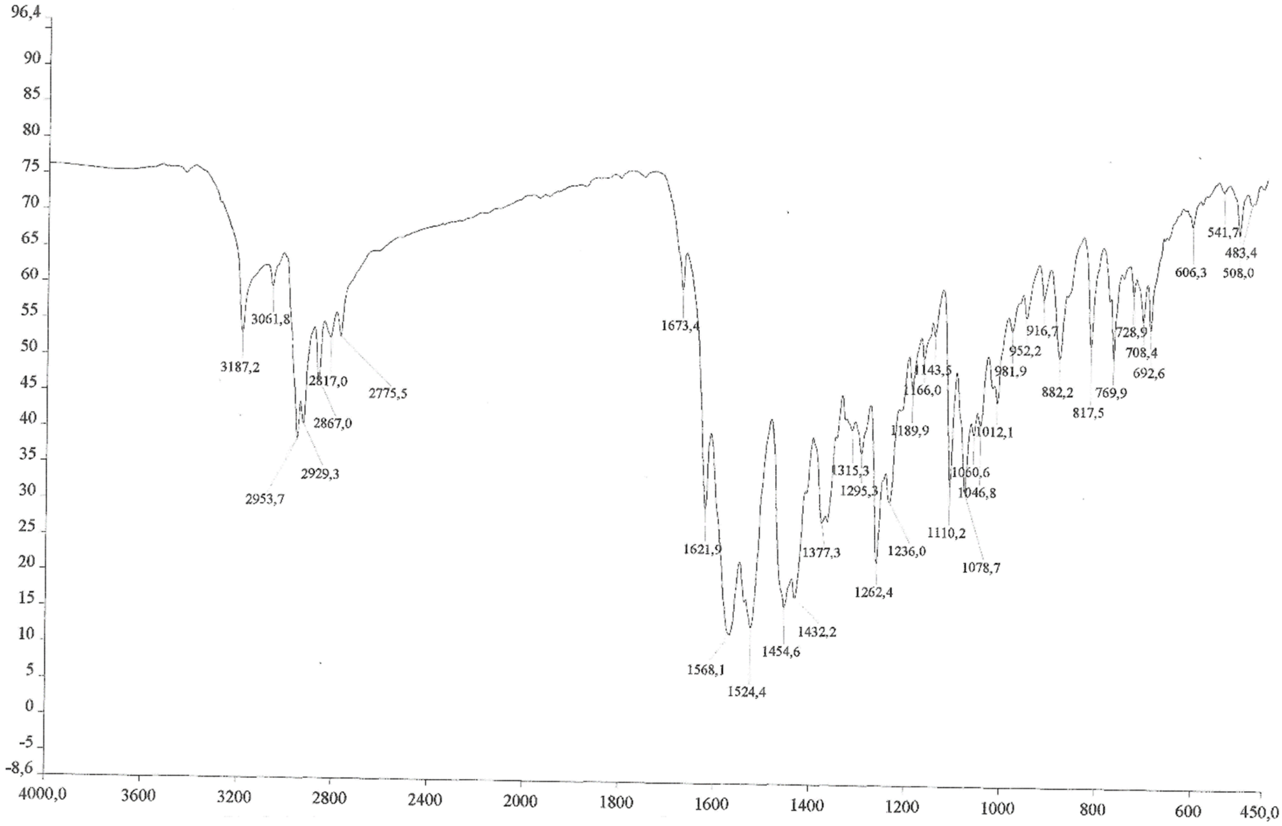

Figure S69. Mass spectrum of compound **5d**

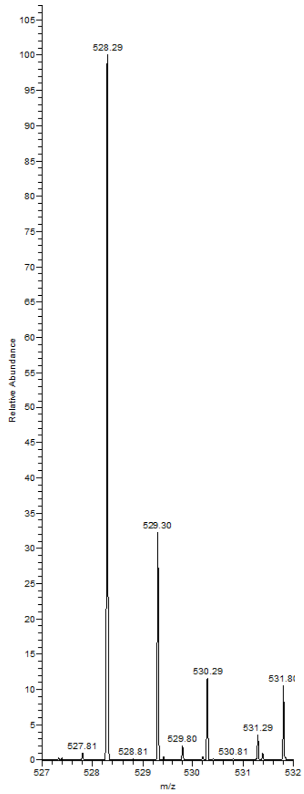

Figure S70. ^{1}H-NMR (200 MHz, $CDCl_3$) spectrum of compound **5e**

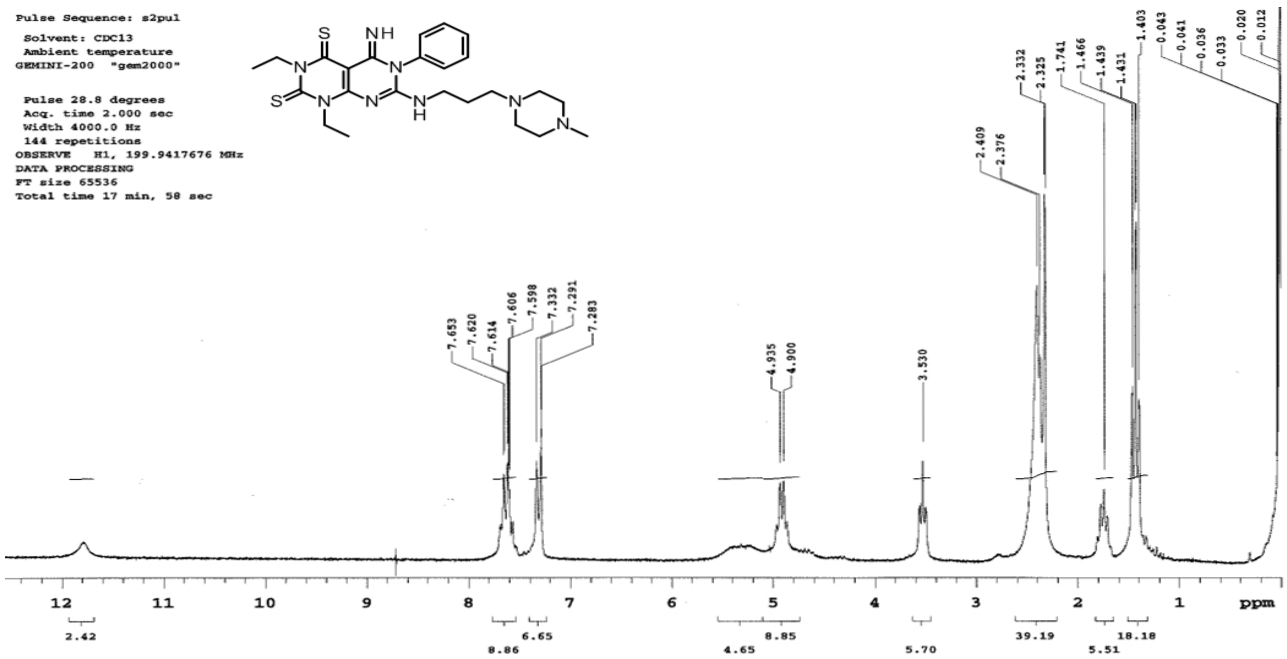

Figure S71. ^{13}C-NMR (101 MHz, $CDCl_3$) spectrum of compound **5e**

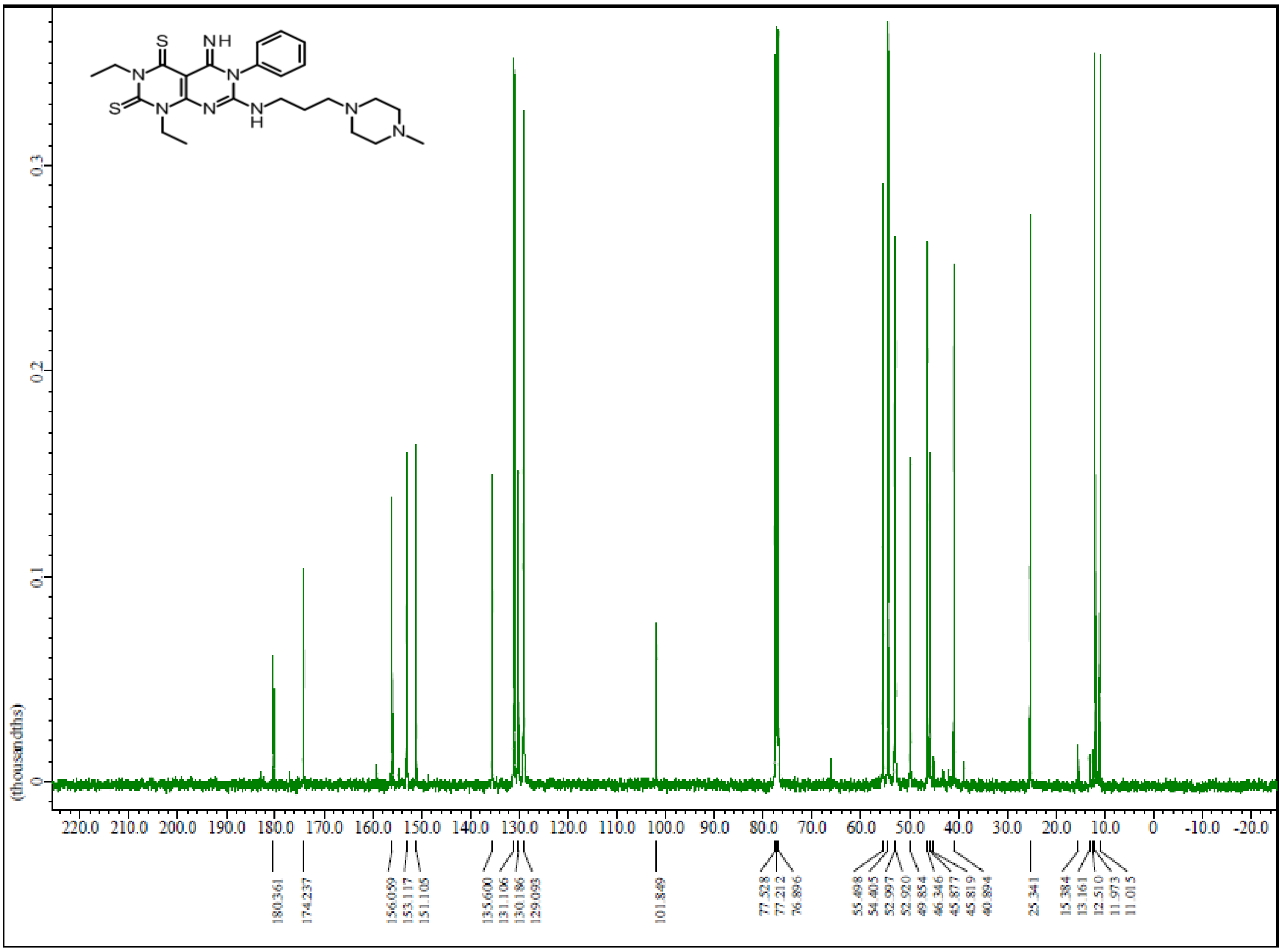

Figure S72. IR (KBr) spectrum of compound **5e**

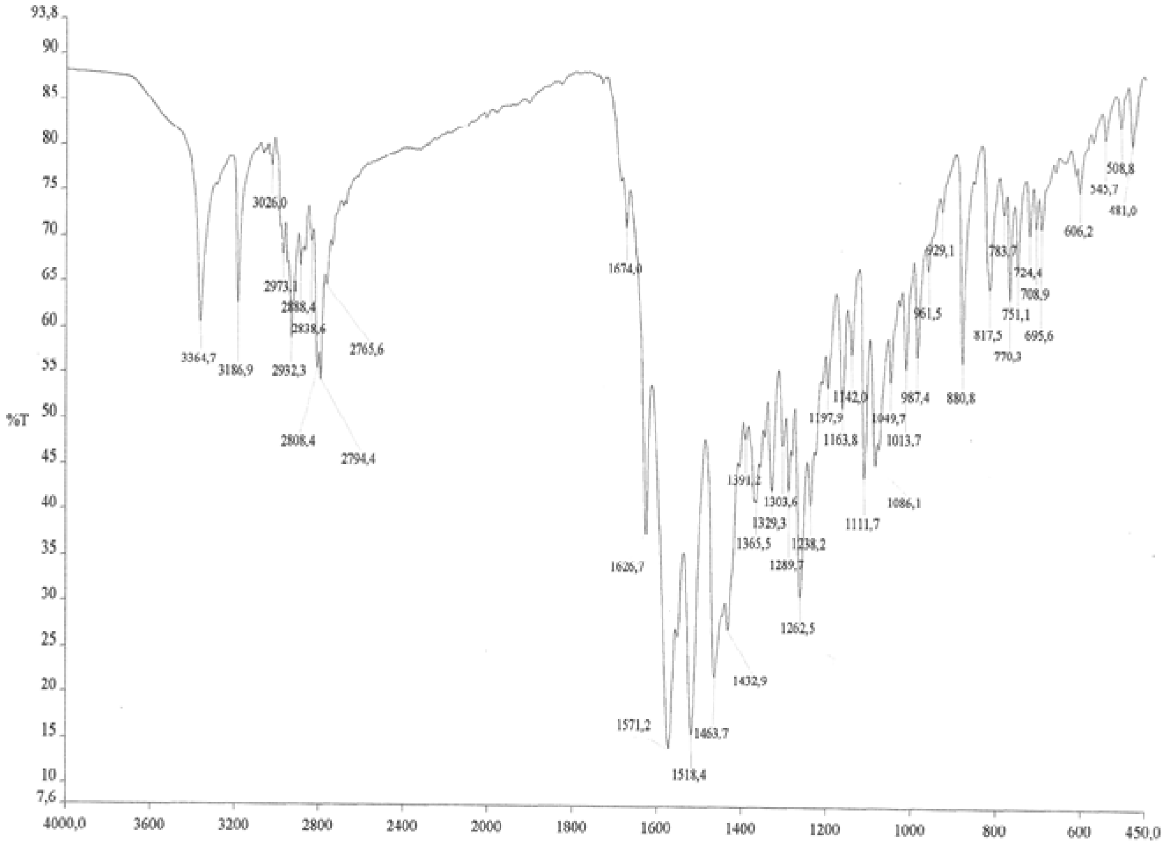

Figure S73. ^{1}H-NMR (200 MHz, $CDCl_3$) spectrum of compound **6a**

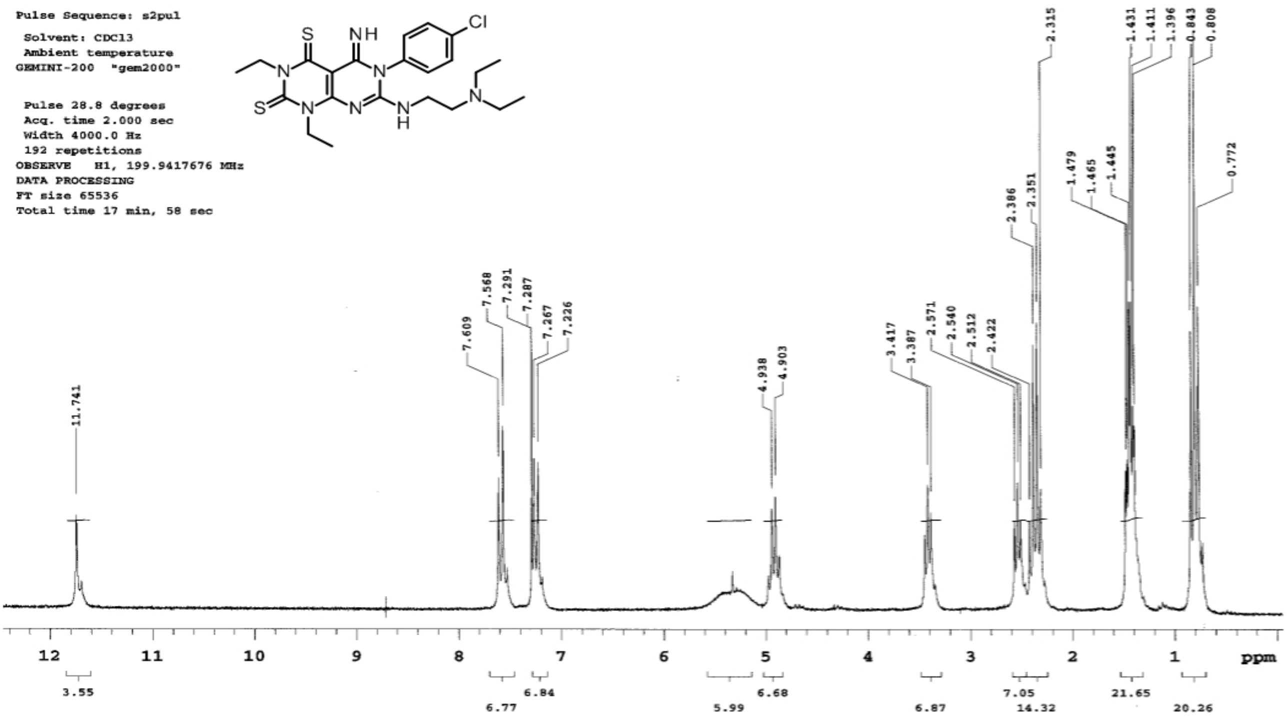

Figure S74. ^{13}C-NMR (101 MHz, $CDCl_3$) spectrum of compound **6a**

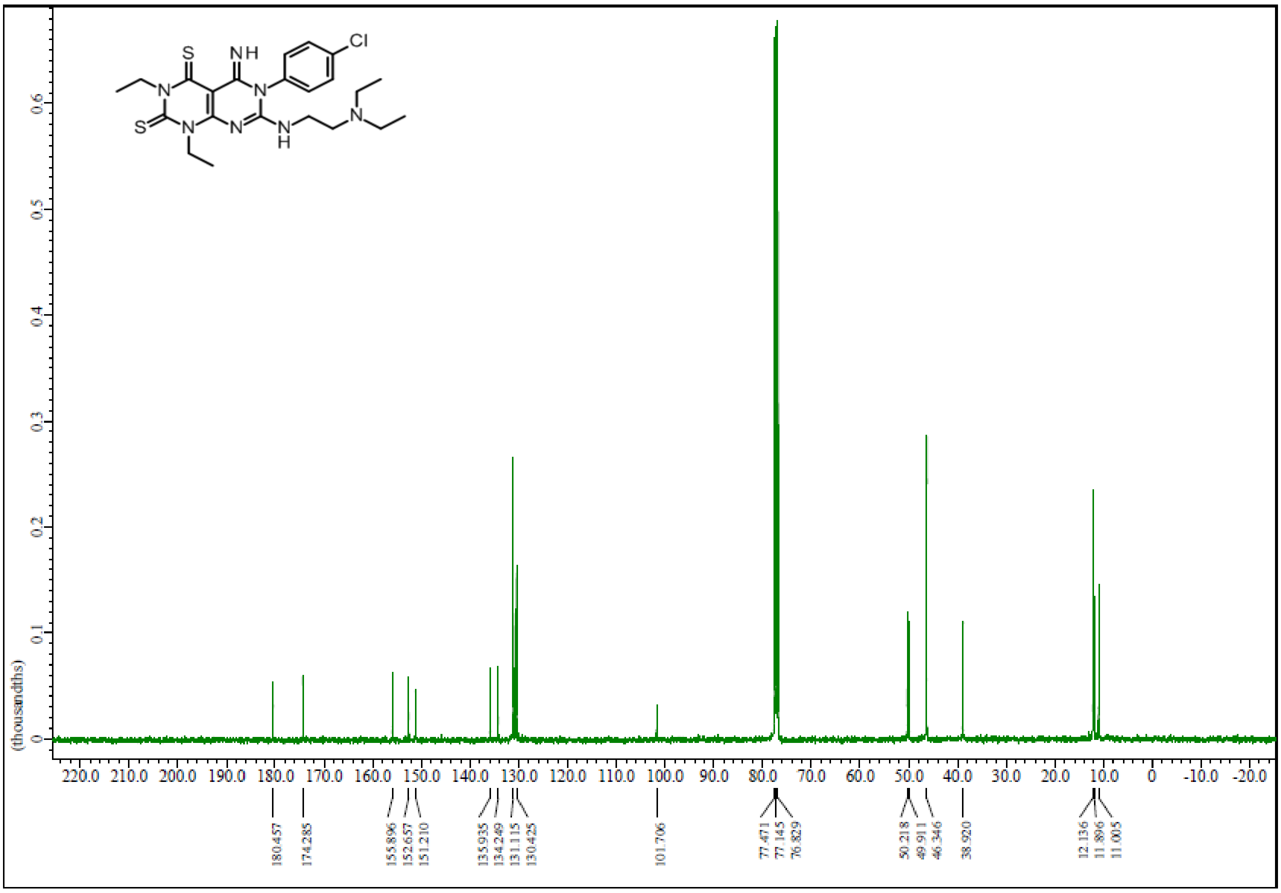

Figure S75. IR (KBr) spectrum of compound **6a**

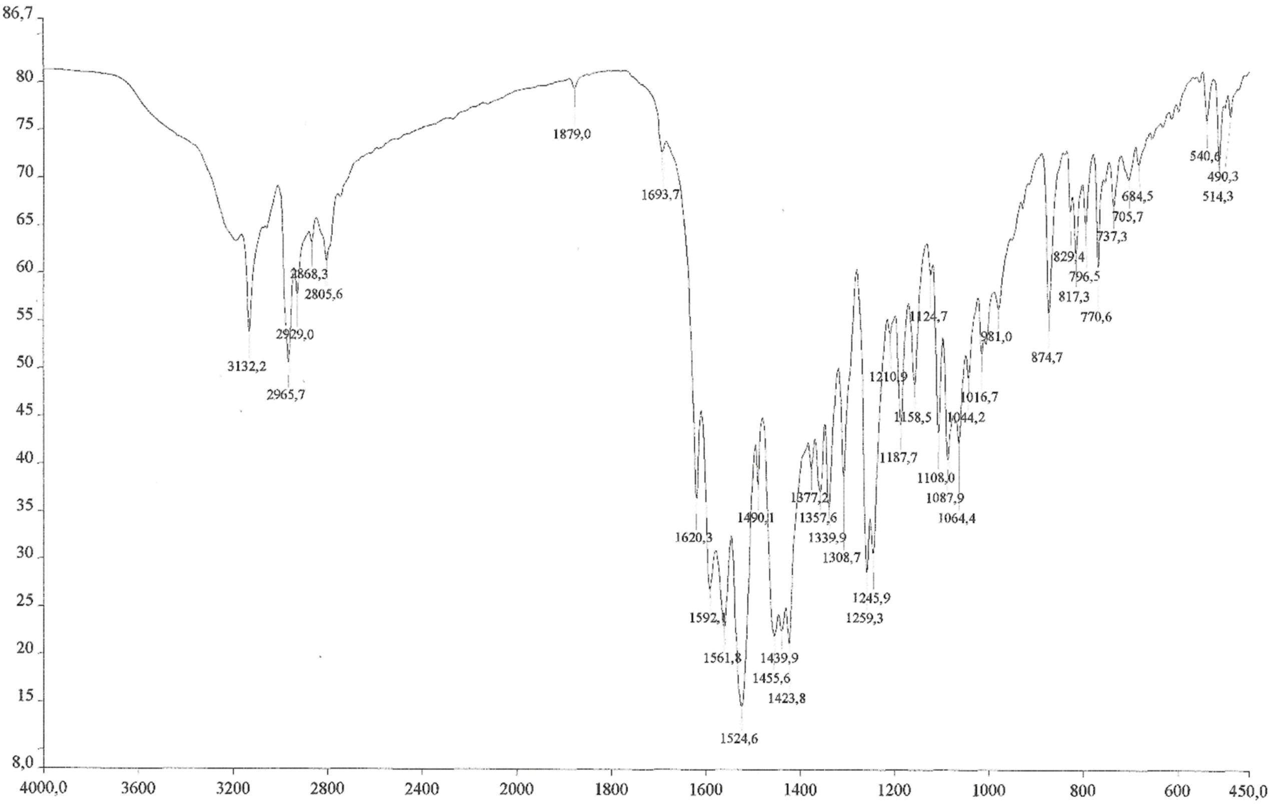

Figure S76. ^{1}H-NMR (200 MHz, $CDCl_3$) spectrum of compound **6b**

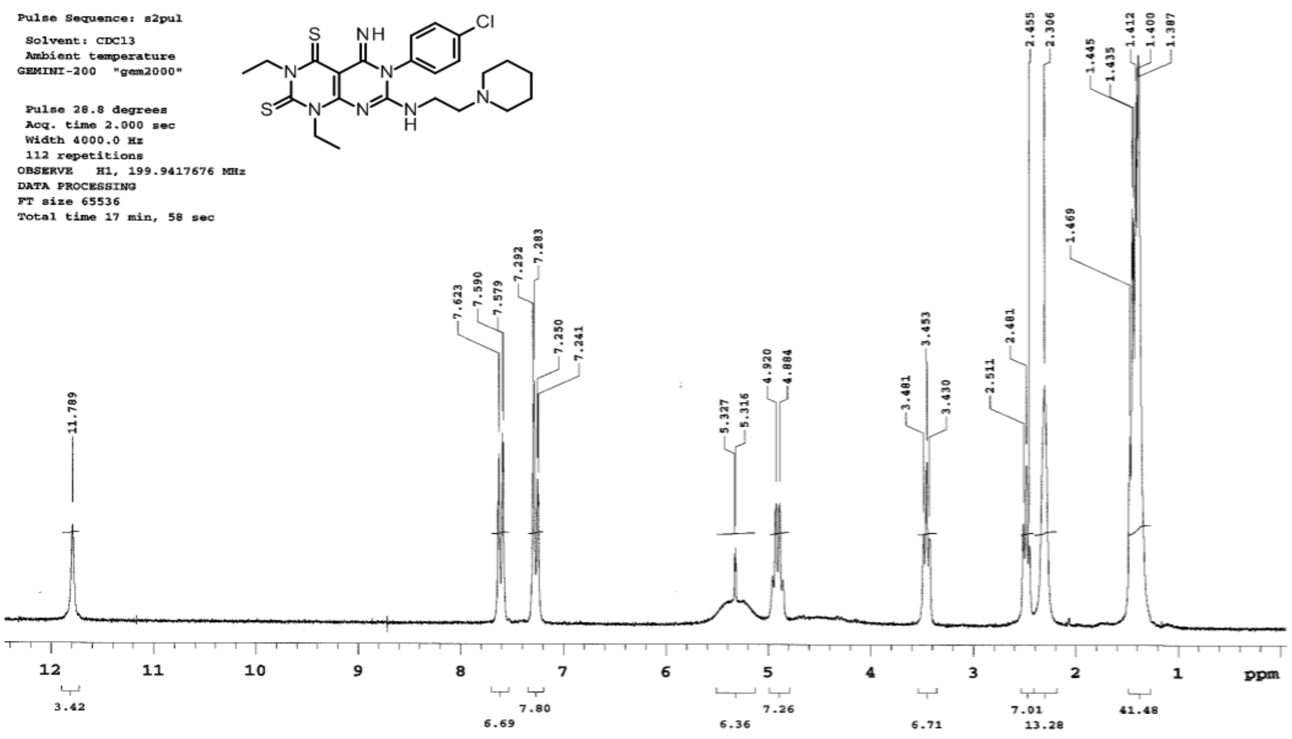

Figure S77. ^{13}C-NMR (101 MHz, $CDCl_3$) spectrum of compound **6b**

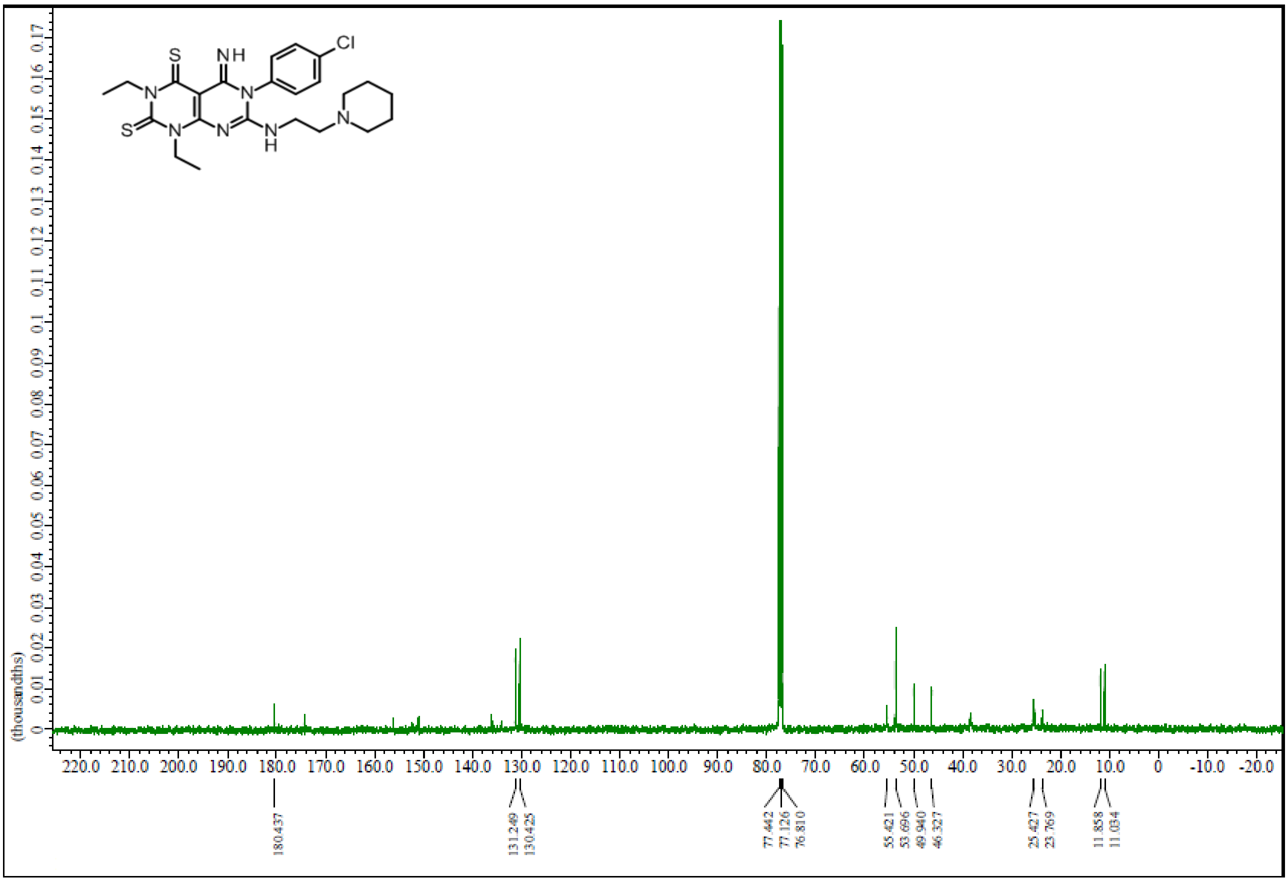

Figure S78. IR (KBr) spectrum of compound **6b**

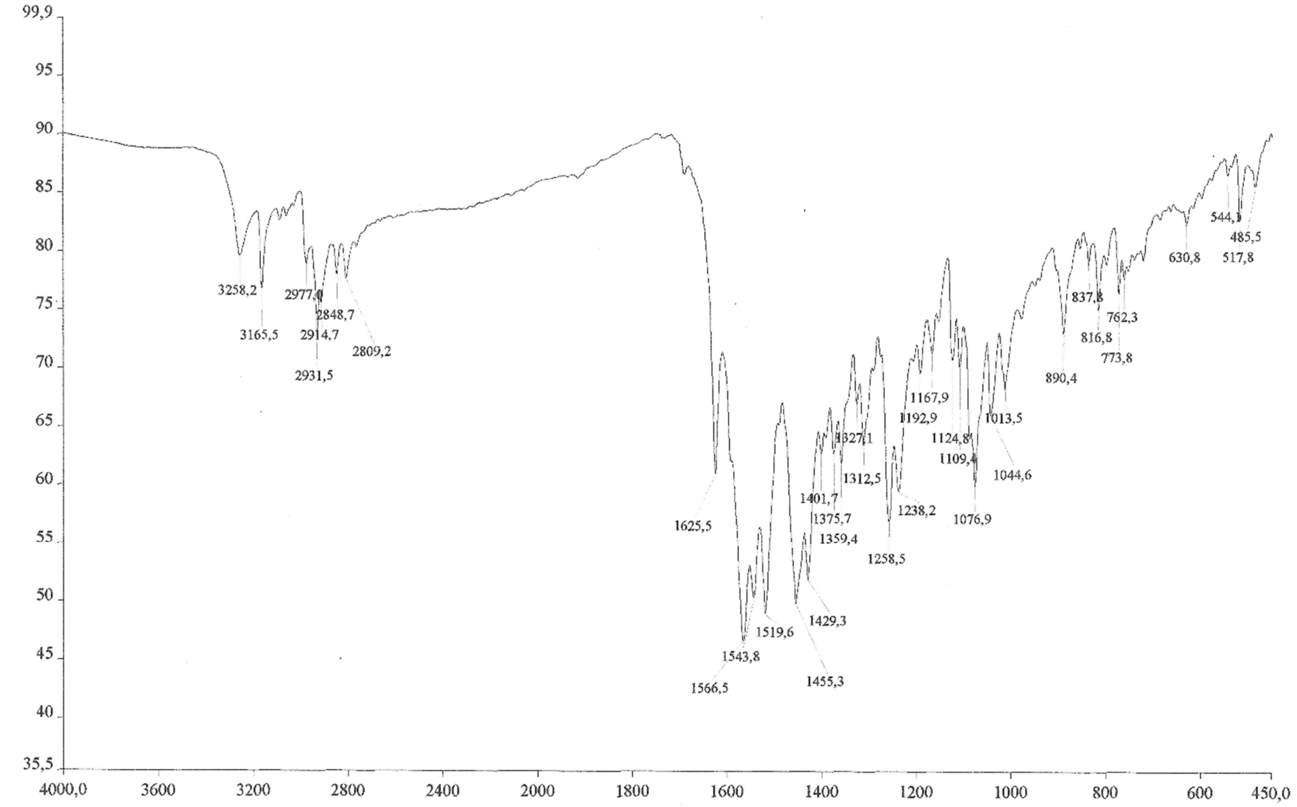

Figure S79. ^{1}H-NMR (200 MHz, $CDCl_3$) spectrum of compound **6c**

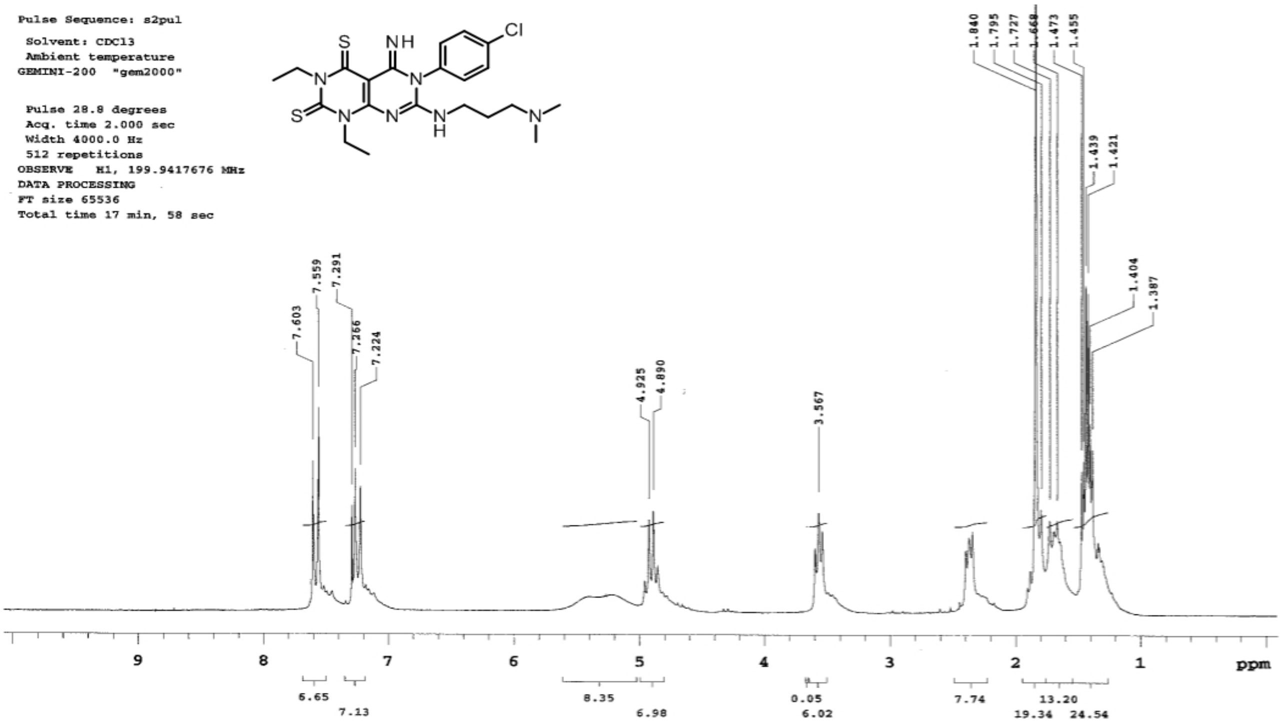

Figure S80. ^{13}C-NMR (101 MHz, $CDCl_3$) spectrum of compound **6c**

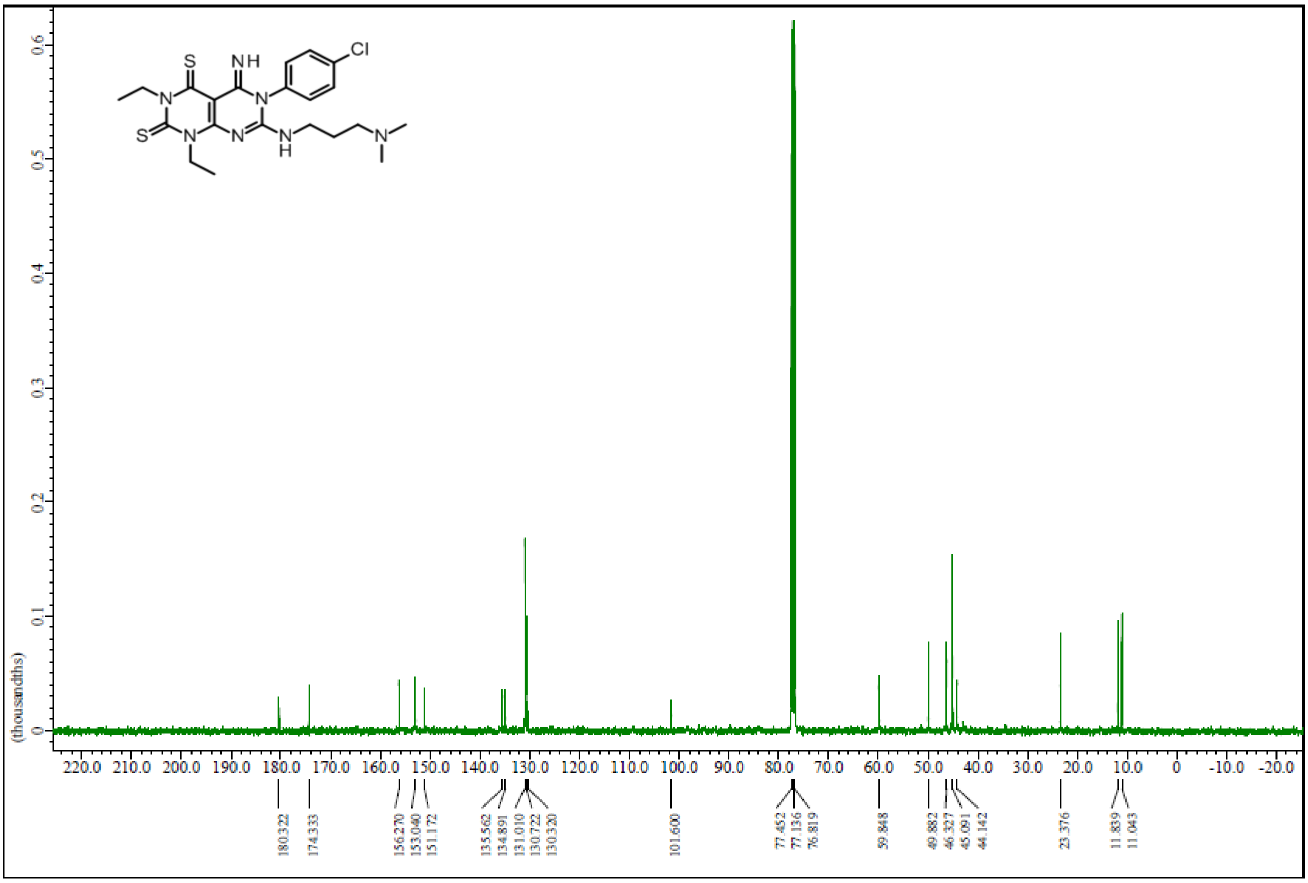

Figure S81. IR (KBr) spectrum of compound **6c**

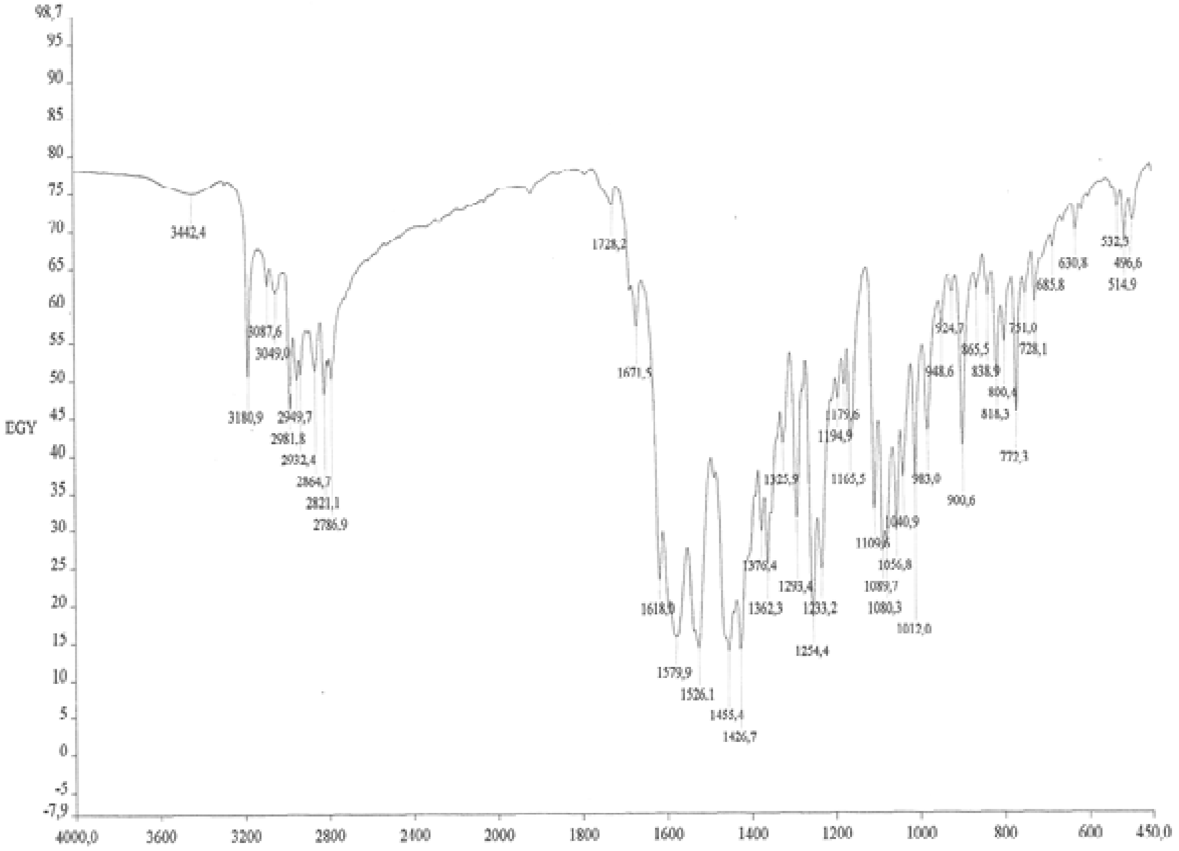

Figure S82. Mass spectrum of compound **6c**

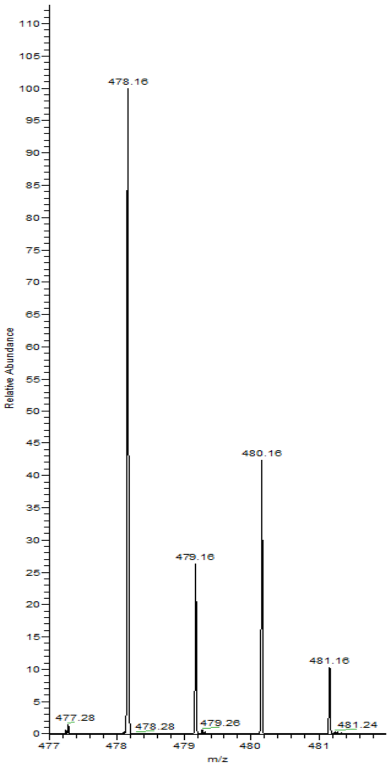

Figure S83. ^{1}H-NMR (200 MHz, $CDCl_3$) spectrum of compound **6d**

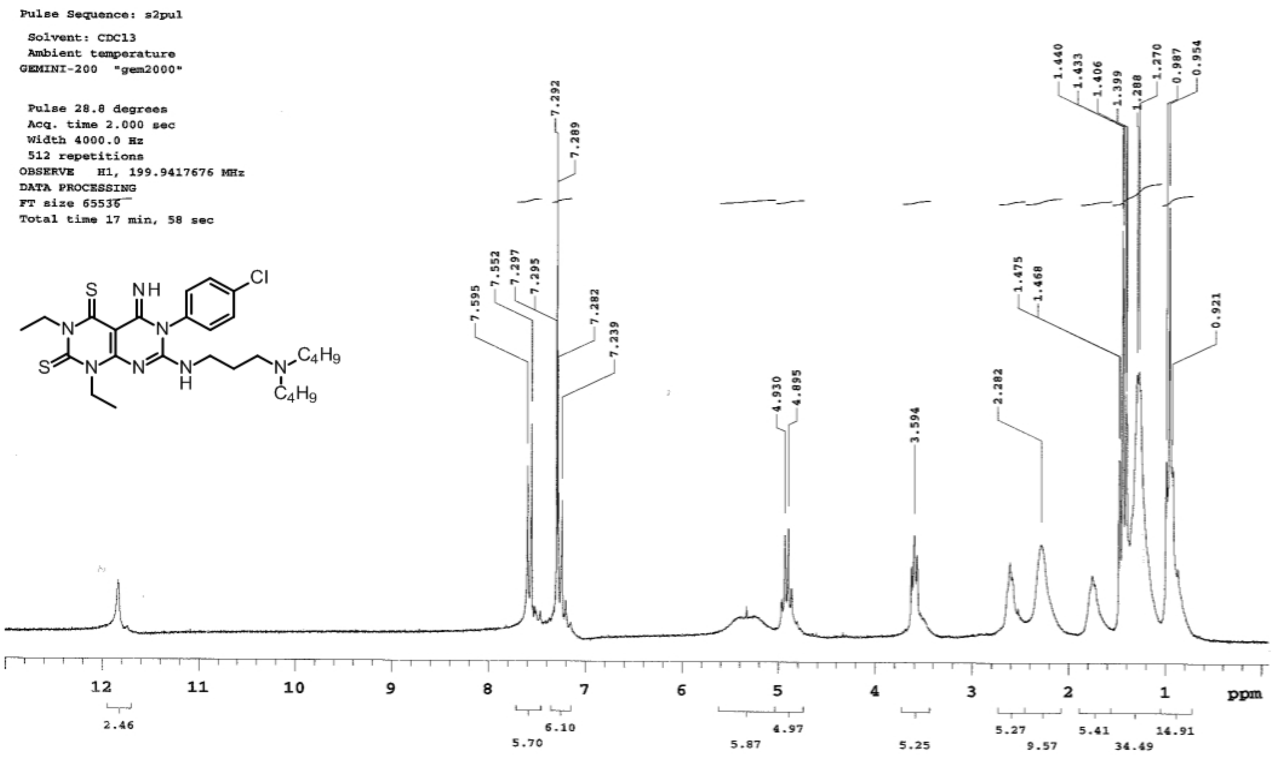

Figure S84. ^{13}C-NMR (101 MHz, $CDCl_3$) spectrum of compound **6d**

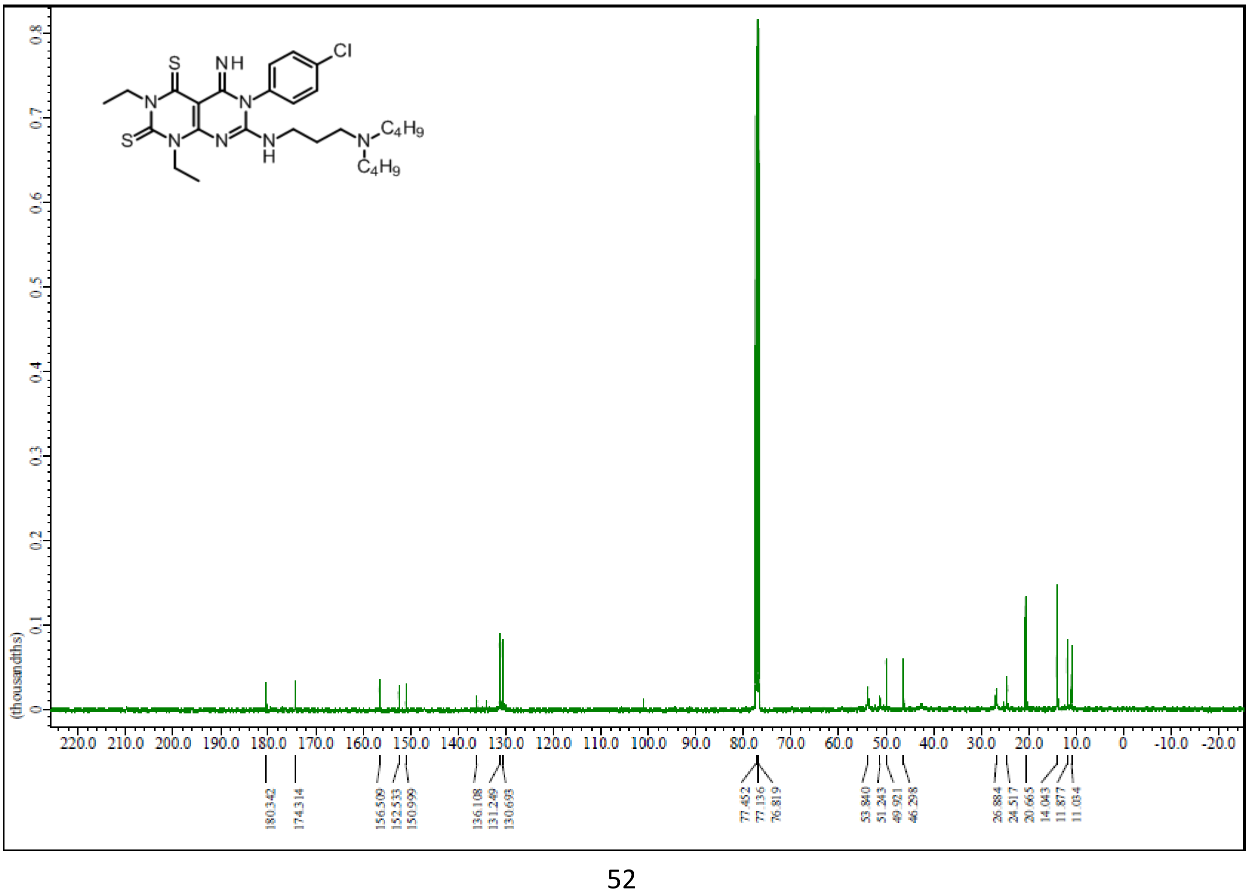

Figure S85. IR (KBr) spectrum of compound **6d**

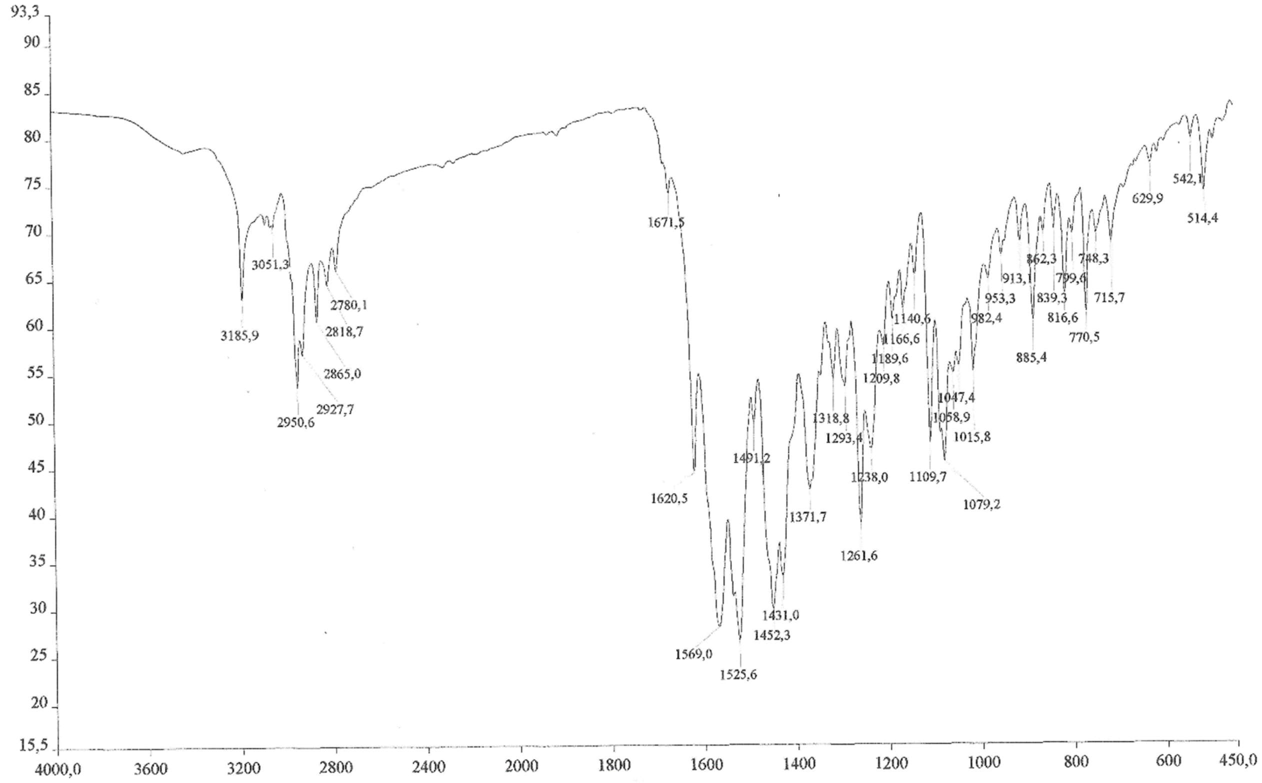

Figure S86. ^{1}H-NMR (200 MHz, $CDCl_3$) spectrum of compound **6e**

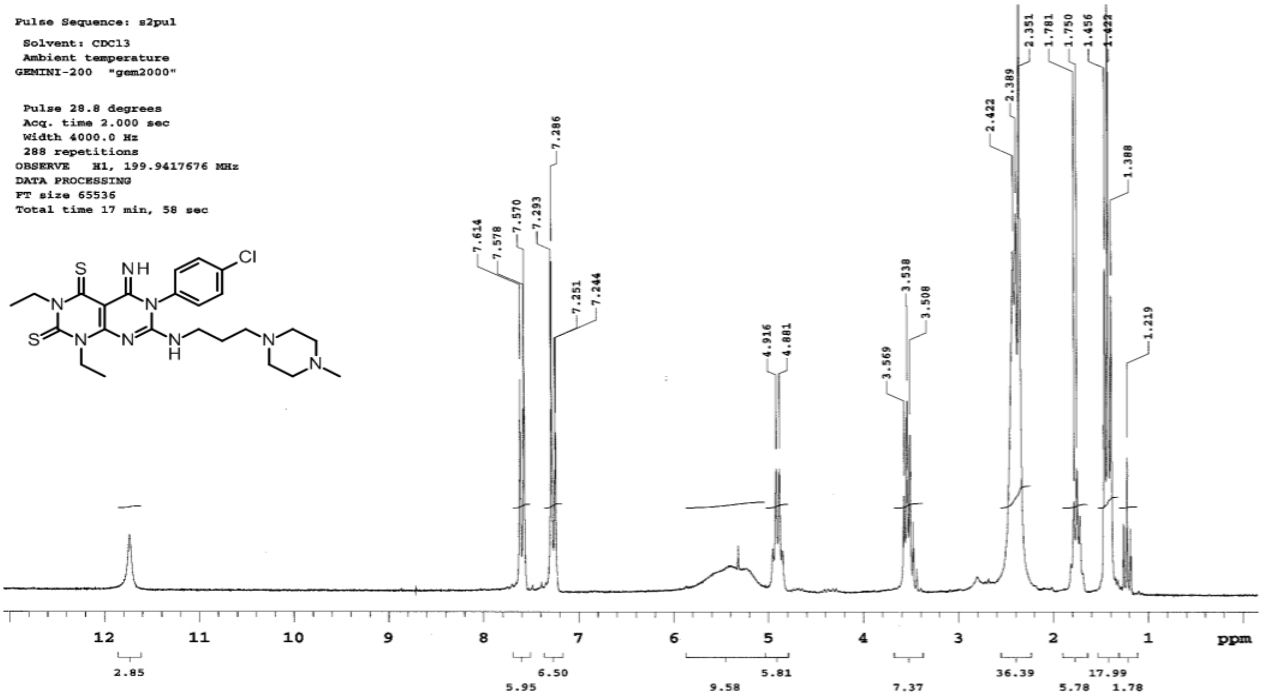

Figure S87. ^{13}C-NMR (101 MHz, $CDCl_3$) spectrum of compound **6e**

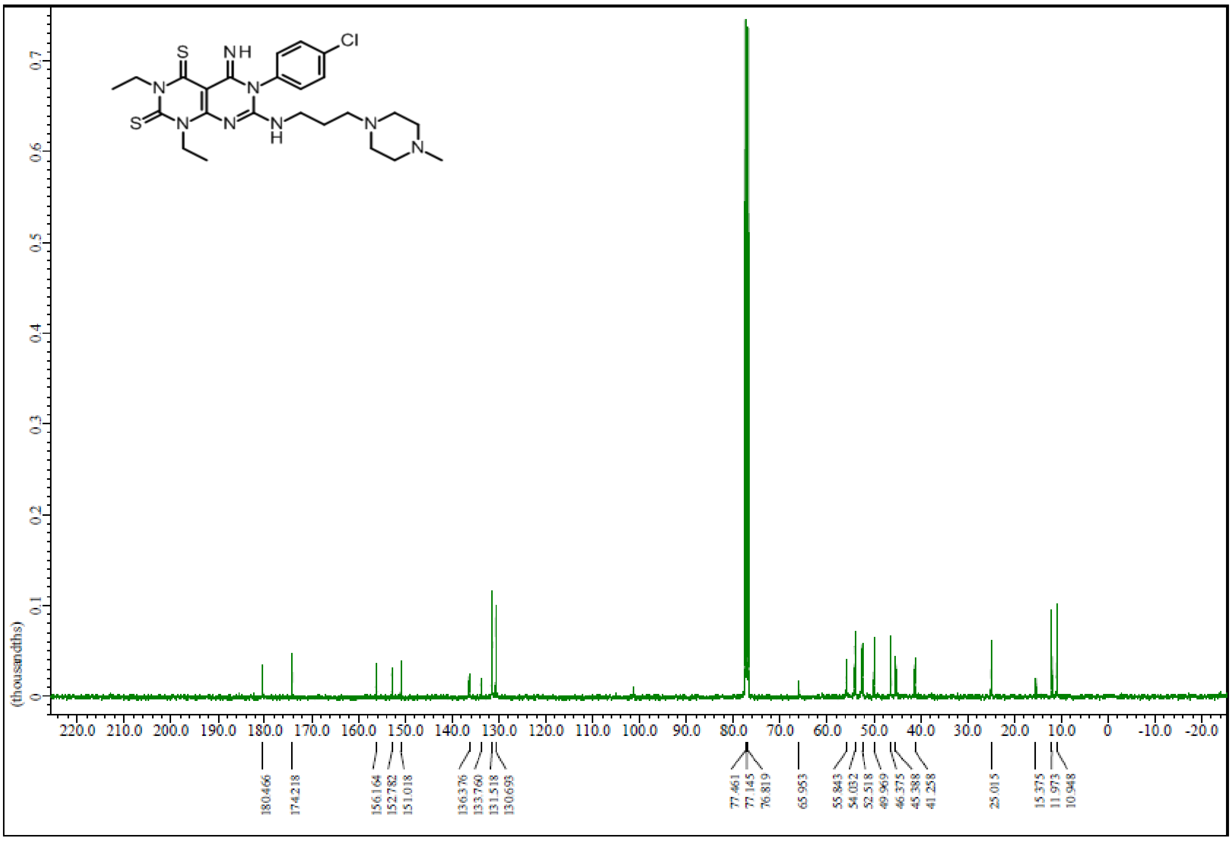

Figure S88. IR (KBr) spectrum of compound **6e**

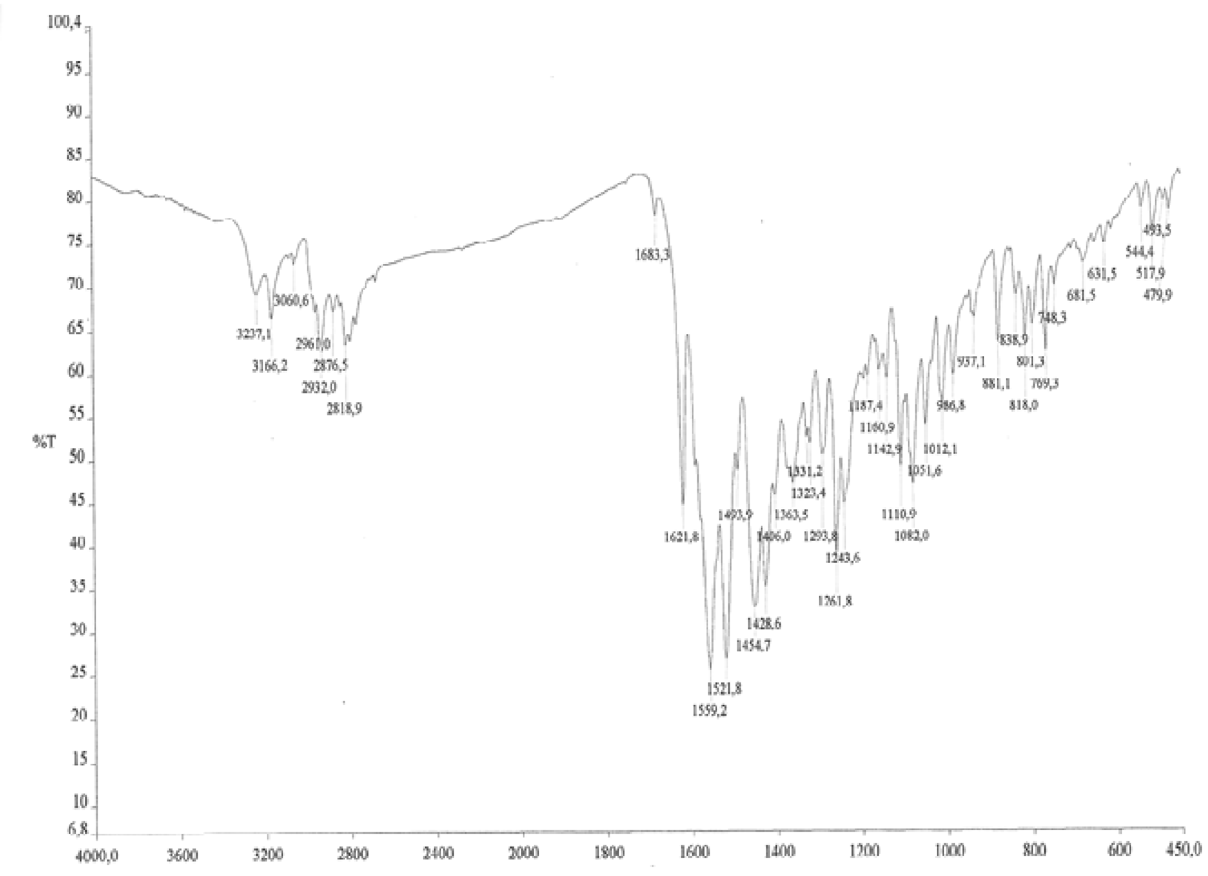

Supplement: Supplementary file 1 [file molecules-26-00557-s001.pdf]
